# An Overview of the Intrinsic Role of Citrullination in Autoimmune Disorders

**DOI:** 10.1155/2019/7592851

**Published:** 2019-11-25

**Authors:** Mohammed Alghamdi, Doaa Alasmari, Amjad Assiri, Ehab Mattar, Abdullah A. Aljaddawi, Sana G. Alattas, Elrashdy M. Redwan

**Affiliations:** ^1^Biological Sciences Department, Faculty of Science, King Abdulaziz University, P.O. Box 80203, Jeddah 21589, Saudi Arabia; ^2^Laboratory Department, University Medical Services Center, King Abdulaziz University, P.O. Box 80200, Jeddah 21589, Saudi Arabia; ^3^Therapeutic and Protective Proteins Laboratory, Protein Research Department, Genetic Engineering and Biotechnology Research Institute, City for Scientific Research and Technology Applications, New Borg EL-Arab, Alexandria 21934, Egypt

## Abstract

A protein undergoes many types of posttranslation modification. Citrullination is one of these modifications, where an arginine amino acid is converted to a citrulline amino acid. This process depends on catalytic enzymes such as peptidylarginine deiminase enzymes (PADs). This modification leads to a charge shift, which affects the protein structure, protein-protein interactions, and hydrogen bond formation, and it may cause protein denaturation. The irreversible citrullination reaction is not limited to a specific protein, cell, or tissue. It can target a wide range of proteins in the cell membrane, cytoplasm, nucleus, and mitochondria. Citrullination is a normal reaction during cell death. Apoptosis is normally accompanied with a clearance process via scavenger cells. A defect in the clearance system either in terms of efficiency or capacity may occur due to massive cell death, which may result in the accumulation and leakage of PAD enzymes and the citrullinated peptide from the necrotized cell which could be recognized by the immune system, where the immunological tolerance will be avoided and the autoimmune disorders will be subsequently triggered. The induction of autoimmune responses, autoantibody production, and cytokines involved in the major autoimmune diseases will be discussed.

## 1. Introduction

Most of the known proteins synthesized by ribosomes through the translation of mRNA are modified in a process known as posttranslational modification (PTM). PTMs are regulatory processes which have a significant role in the functional diversity, stability, and interactions of proteins with other molecules. These modification processes include physical and chemical changes of a protein or its particular amino acid. The physical modification involves protein folding facilitated by a chaperone protein, while the chemical modification has a different mechanism and different forms. The common forms of PTM are trimming or proteolysis, ubiquitination, and covalent modifications. There are different types of covalent modifications of the protein, which occur by the addition of chemical groups such as in phosphorylation, acetylation, hydroxylation, and methylation. The addition of a complex molecule is another mechanism of PTM, like glycosylation, AMPylation, and prenylation. The modification of amino acids is also another form of PTM, which involves deamidation, eliminylation, and citrullination. In this review, we will consider the citrullination process that is catalyzed by calcium-dependent enzymes known as peptidylarginine deiminase enzymes (PADs) since it is important in the induction and subsequent diagnosis of autoimmune disorders. Furthermore, the autoimmune responses attributed to citrullinated proteins will be involved. In addition, this review will shed some light on the prevalence of citrullination-related diseases in the Saudi population.

## 2. Data Collection

### 2.1. Search Strategy and Study Selection

For data collection, we conducted an electronic search for the identification of comprehensive studies and eligible data. We searched the Medline database through PubMed and Scopus databases to obtain related articles published up to November 2018. The articles used were scanned based on titles and abstracts. The following subject terms were used in the search: *Citrulline*, *Citrullination*, *Anti-CCP*, *PAD*, *Peptidylarginine deaminase*, *antimutated*, *antimodified vimentin*, *autoimmune disorders*, *Saudi Arabia*, or *combined autoimmune disorders*, *Saudi Arabia*. All these keywords alone or combined with*Rheumatoid Arthritis*, *Multiple Sclerosis*, *Alzheimer's*, *Systematic Lupus Erythematosus*, and *Myelin demyelination* were used. Then, the data were classified according to different categories: epidemiology, clinical features, laboratory values, management, and reviews.

### 2.2. Data Presentation

The analytical data are presented in Tables 1–3, and clinical features are presented in Tables 4 and 5. In addition, studies that indicate the prevalence of diseases in the Saudi population are presented in Tables 6 and 7. Serological tests obtained from related studies are presented inTables 8 and 9. Eventually, a total of more than 400 studies were identified through our literature search.

## 3. Citrullination

**Figure 1:**
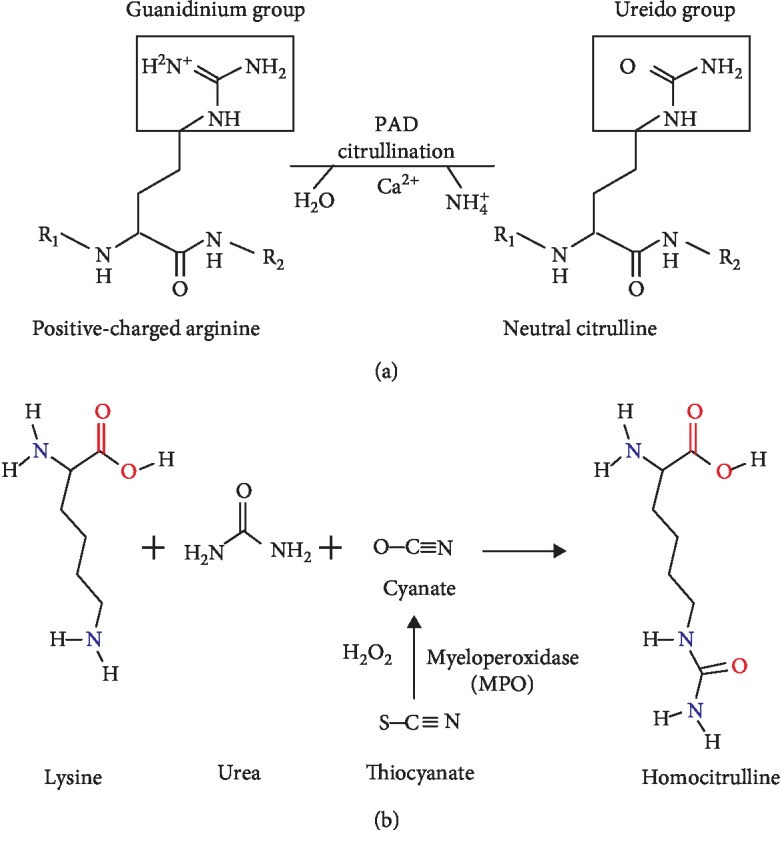
The chemical reaction of (a) citrullination and (b) carbamylation.

Citrullination is a chemical process and has a significant role in different physiological processes which are involved in many pathological diseases. Citrullination or deimination is a posttranslational modification of protein in which arginine amino acid is converted into citrulline amino acid. This process is catalyzed by peptidylarginine deiminase (PAD) enzymes, which are activated by high calcium (Ca^++^) concentration. The protein citrullination process was first detailed by Rogers and Simmonds in 1958 [1] as the process in which peptidylarginine is converted to peptidylcitrulline. Citrulline is a nonstandard amino acid that is nongenetically encoded, but it is produced at the posttranslation level only [2]. During this hydrolytic reaction, the target protein mobility in SDS-PAGE will shift [3, 4], yielding a noncharged citrulline amino acid and neutral urea through the hydrolysis of the strongly basic positively charged side chain of arginine by water (Figure 1). This charge shift affects protein structure, protein-protein interactions, and hydrogen bond formation, and it may cause protein denaturation [5, 6]. Citrullination can involve a wide range of proteins, integrated cell membrane, cytoplasm, nucleus, and/or mitochondrial proteins [7].

### 3.1. Chemical Basis of Citrullination

Chemically, citrullination or deimination is a process catalyzed by specific enzymes called PADs and it modifies the guanidinium group of the arginine amino acid and the ureido group of the citrulline amino acid. This conversion is accompanied by the loss of the arginine positive charge and production of ammonium. Furthermore, this modification is an irreversible process, i.e., there is no decitrullination reaction [7]. The reaction was first described by Fearon [8] in 1939. In this reaction, hydrolysis of the nitrogen atoms of the arginine side chain results in the formation of citrulline and ammonia as a side product. Citrullination occurs in alkaline solutions at room temperature [9]. Due to the guanidine group, arginine has a +1 charge at physiological pH, whereas citrulline has no charge (neutral) (Figure 1). Thus, the overall charge of the protein is decreased by citrullination [3, 10]. During this reaction, losing the positive charge results in protein conformational changes which may modify the binding and unfolding properties of the protein, subsequently affecting its function and half-life [11]. The conversion of arginine to citrulline also leads to changing the acidity of the amino acid side chain, changing the isoelectric point (*pI*) of arginine from 11.41 to 5.91 for citrulline [12], the formation of hydrogen bonds, and the interaction between amino acid residues, which substantially affect the peptide or another one. Thus, the protein is formed with a conformational, functional alteration and new protein half-life [13].

Citrullination or deimination is a normal physiological reaction that occurs during cell death. Therefore, the immune system normally is not in conflict with citrullinated proteins. During apoptosis, the change in the physical properties of the dying cell is followed by the clearance and ingestion of these cells by phagocytic cells. A defect in the clearance system either in terms of efficiency or capacity may occur due to massive cell death, and this results in the accumulation and leakage of PAD enzymes and citrullinated peptide from the necrotizing cell which may be encountered by the immune system [14].

## 4. PAD Enzymes

PAD enzymes were described in 1977 as one enzyme family of PTM [15], and now it also known as cysteine hydrolases [16]. In humans, PADs are a family of calcium-dependent enzymes composed of five isozymes (1, 2, 3, 4, and 6) [17], which have 50% sequence similarity [18]. PADs are found in abundant cells and tissues across the body, including the uterus and epidermis (PAD1); brain, skeletal muscle, secretory glands, inflammatory cells, and several cancer cell lines (PAD2); keratinocytes and hair follicles (PAD3); granulocytes and cancer cells (PAD4); and embryos and oocytes (PAD6). Interestingly, all PADs are known to be found in the cell cytoplasm except PAD4, which is the only isozyme that has been found in the nucleus where it plays a role in histone deimination [2, 17], even though recent studies found that PAD2 may also be found in the nucleus [19, 20]. In addition to the cytoplasm and nucleus, new reports suggest that the PAD isozymes also exist in granules (PAD4) [21] and mitochondria (PAD2) [20].

In prokaryotes, *Porphyromonas gingivalis* is a major periodontal pathogen involved in destructive periodontal disease and it is a unique prokaryote expressing a PAD enzyme [22, 23]. *P. gingivalis* synthesizes and releases PAD (PPAD) in membrane vesicles [23]. Compared to human PAD, PPAD is a calcium-independent enzyme [23, 24]. The application of a specific PAD2/PAD4 inhibitor to block extracellular PAD activity is very effective in the treatment of citrullination disorders. Such therapy could prevent the production of citrullinated autoantigens and immune complexes [14].

### 4.1. PAD Substrates and Activation

The calcium (Ca^+2^) ion is responsible for the activation of certain enzymes in the living cell, including the peptidylarginine deiminase (PADs) enzymes. During apoptosis, the Ca^+2^ concentration increased by 100-1000-fold from the normal. The Ca^+2^ concentration in the activated cells that were used for PAD activity is around 10 *μ*M, which is higher than the physiological level in nonactivated cells (100 nM). The high calcium concentration has been suggested to occur locally in cells or in extreme conditions like apoptosis or necrosis [25]. Ca^+2^ attaches to specific binding sites of PADs. For example, PAD4 has five calcium-binding sites, two of them located in the C-terminal domain and the remaining three located in N-terminal subdomain 2 of the enzyme [16, 18]. Likewise, the kinetics of calcium ion binding to PAD2 showed that three molecules of Ca^+2^ can bind one molecule of PAD. Thus, it is proposed that PAD enzymes have three low-affinity calcium-binding domains [2]. It was found that the calcium sensitivity of PAD3 differs from one isotype to another, depending on the nature of the substrate [26]. Since PADs are calcium-dependent hydrolases, they cannot act at low physiological calcium levels, being activated by the calcium homeostasis distortions leading to the increase in the cellular calcium levels. Recently, available information on the structural properties of human PADs and the mechanisms of their calcium-induced activation was systematically analyzed together with the prevalence of functionally important intrinsically disordered regions in these proteins [27]. PAD enzymes are represented in many leukocytes, richly in neutrophils and monocytes, where at certain positions of autologous peptides they cause conversion of arginines to citrullines [28]. Within the protein, not all target arginines are citrullinated equally; about 80-90% of the arginines which are positioned after aspartic acid are citrullinated, whereas arginines close to glutamic acid are hardly citrullinated (0%-5%) and those which are located near the N-terminus are poorly citrullinated too (Table 1) [3].

### 4.2. PAD Isotypes: Distribution and Normal Physiology

Reports identified three types of PAD: PAD I (epidermal type), PAD II? (muscle type), and PAD III (hair follicle type); each of them are different in their tissue distribution, activities towards synthetic substrates, and antigenic properties [29]. In humans, there are five PAD isotypes: PAD1, PAD2, PAD3, PAD4, and PAD6. At the protein level, these PADs are highly conserved, have more than 50% sequence similarity [30, 31], and have a positive role in normal physiological functions, such as skin keratinization and homeostasis, neuron insulation, and gene regulation and expression [27]. PAD enzymes are distributed in a wide range of cells and tissues, and each type has a tissue-specific manner of expression (Tables 2 and 3) [31]. Citrullination is an enzymatic conversion that normally occurs in many biological processes such as epidermal differentiation [32], hair follicle formation [33], and maturation of the myelin sheath during central nervous system development [34]. The earliest reports suggested that the presence of citrullinated proteins is specific to the synovium in rheumatoid arthritis (RA) patients [35], while new reports revealed that citrullinated proteins are also present in the synovium of nonrheumatoid arthritis inflammations [36, 37].

**Figure 2:**
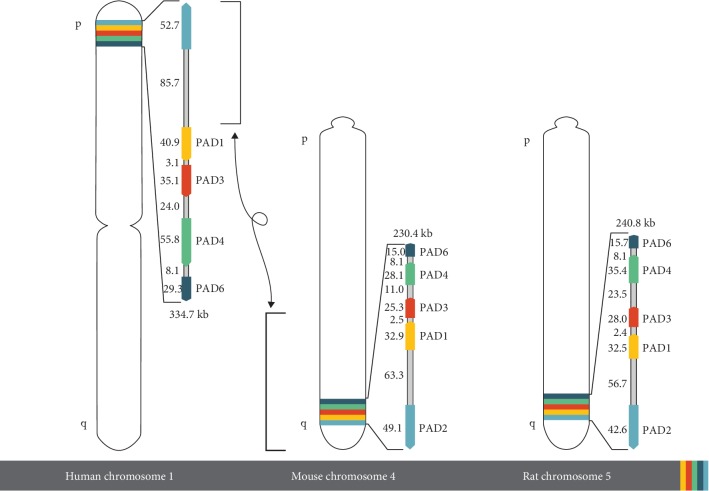
PAD gene cluster organization. Ideograms showing the location and orientation of the PAD gene clusters of human chromosome 1, mouse chromosome 4, and rat chromosome 5.

The genes encoding each of the mammalian PAD isotypes are named PADI and are clustered on a single locus on chromosome 1. PADI genes appear to have the same exon/intron structure and have high sequence similarity in exons. All PADI genes in humans are clustered on the short arm of chromosome 1 close to the telomere (1p36.1), and are included approximately within a 334.7 kb region (Figure 2) [38]. The genes of PAD1, 3, and 5 types are very close and are compacted within a 160 kb region. Furthermore, they have the same transcription orientation [39]. PADI2 is the largest gene, which shows an inverse direction to the other four PADIs, while the PADI6 gene is the smallest one in humans [40].

#### 4.2.1. Peptidylarginine Deiminase Type I (PAD1)

PAD1 enzymes are mainly expressed in the epidermis and uterus [41, 42]. GenBank blast search clarifies the sequence tag entries with the human PAD type I cDNA sequence. This search shows several expressed sequence tags from tissues of the human (uterus, pancreas, and colon), rat (eye), and mouse (thymus, uterus, skin, and vaginal epithelium), demonstrating that PAD1 is not skin specific, but it is more widely expressed. The human PAD1 gene has 16 relatively short exons occupying 63 ± 851 bp, which interfere with 15 introns ranging from 104 bp to more than 16.9 kb [39]. PAD1 is encoded by a 3.8 kb mRNA containing a 3′-UTR of about 1600–1700 nt [2]. PAD-induced citrullination has been illustrated in different physiological processes and pathological conditions. Physiologically, PAD1 are involved in the keratinization of the skin, as well as implicated in psoriasis [13]. In the epidermis, during keratinocyte differentiation, keratin proteins particularly K1 and K10 and the keratin-associated protein filaggrin are citrullinated [43, 44] by PAD1, which is important for the regulation of cornification of the epidermis and preserving the barrier function of superficial keratinized epidermal cell layers [45]. The cornification of the epidermis is stimulated by the reduction in the flexibility of the keratin cytoskeleton during citrullination [46]. However, in normal skin hemostasis, the keratin protein (K1) is citrullinated and degraded at low levels to reduce cornification [16]. Filaggrin is a filament protein aggregate resulting from the citrullination of profilaggrin; this filaggrin cross-links the keratin filaments to form solid structures [47]. During citrullination of the cytokeratin, the lost charge is believed to cause disassembly and degradation of the cytokeratin-filaggrin complex [45]. Subsequently, filaggrin is more degraded to produce amino acids that constitute the Natural Moisturizing Factor (NMF) which has an important role in skin moisturizing [48].

Although PAD1 is expressed throughout the epidermis mainly in keratinocytes, other PADs were detected in skin layers such as PAD2 in the suprabasal layers and PAD3 in differentiated keratinocytes and hair follicles [38]. The deficiency of citrullination in the epidermis results in extreme cornification and inflammatory response. Psoriasis is the only disorder caused by PAD1 deficiency, and the factor(s) casing the enzyme catalytic activity deficiency is unknown [2, 30, 49, 50].

#### 4.2.2. Peptidylarginine Deiminase Type II (PAD2)

PAD2 is the most broadly expressed isotype of PAD [2]. It is presented in a multiple organs, such as the brain, female genital tissues, skeletal muscle, and cells of the hematopoietic lineage [51, 52], as well as in the human mammary gland [53]. The human PAD2 gene has 16 relatively short exons occupying up to 520 bp, which interfere with 15 introns that range from 729 bp to more than 14.2 kb. Interestingly, it has been noted that the human PADI genes I, III, and IV also contain 16 exons [54]. The expression of PAD2 was found to be regulated at both transcriptional and translational level [47, 55]. The well-known protein substrates for PAD2 are myelin basic protein (MBP), glial fibrillary acidic protein (GFAP), vimentin, actin, and histones [16]. PAD2 is important for the stability of the myelin sheath and the plasticity of the brain cells [13]. PAD2 in the CNS is known to citrullinate MBP and GFAP, and it is mainly expressed by astrocytes, oligodendrocytes, and microglial cells [3]. Native MBP forms very tight and compact myelin sheaths and contains several arginine amino acid targets [56]. The plasticity of the CNS at a young age is facilitated by the citrullination of MBP, and the ratio of citrullinated MBP (MBP-Cit) and total MBP (MBP-total) changes rapidly after birth [3]. In children less than 2 years, all MBP is citrullinated. Citrullination activity decreased with age. Above 4 years, the ratio of MBP-Cit/MBP-total is reduced to 18%; this ratio remains constant in adults. Therefore, the alterations of MBP-Cit/MBP-total correlate with the brain plasticity in young children [2, 34].

Vimentin is an intermediate filament network that is citrullinated during apoptosis [57]. Vimentin is a cytoskeletal protein that is 466-amino-acids long, and it contains about 9.2% arginine at its non-*α*-helical head domain which represents an intermediate filament. Vimentin is known as a substrate for PAD2, where it citrullinates the non-*α*-helical head domain arginine [58]. For example, PAD2 play an important role in apoptosis by inducing vimentin citrullination, specifically in macrophages [59]. PAD2 expression was found to be upregulated during monocyte differentiation into macrophages [60]. In macrophages, the elevated concentration of calcium induces PAD2 to citrullinate vimentin causing the breakdown of the intermediate filament network potentially to control the apoptotic process [55]. PAD2 and PAD4 are highly implicated in the pathology of inflammation and neurodegenerative [55, 61] and cancer diseases [62]. Overactivity of PAD2 is implicated in multiple sclerosis [13]. Many findings indicated that the beginning and progression of neurodegenerative human diseases such as Alzheimer's disease (AD) and multiple sclerosis (MS) are affected by the dysregulation of PAD2 activity. In addition, the primary open angle glaucoma optic nerve damage was proposed to be influenced by PAD2 and MBP citrullination [63].

#### 4.2.3. Peptidylarginine Deiminase Type III (PAD3)

PAD3 is localized in the skin epidermis and is highly linked to the hair follicle and epithelium targeting the intermediate filament-associated protein trichohyalin that appears in the inner root sheath of a hair follicle [64]. PAD3 gene is located on chromosome 1 (p36.1) distal to the PAD1 gene. The PAD3 enzyme is encoded by a 3.1 kb mRNA [65]. PAD type III cDNAs have been cloned from sheep hair follicles and from rat and mouse epidermis. The obtained PAD3 from all these sources was found to have a similar calculated molecular mass composed of 664 amino acids [65]. By using reverse transcription PCR and the technique of the rapid amplification of cDNA ends, a full-length cDNA of PAD3, which is about 3142 bp, was cloned from cultured human keratinocytes. This cDNA has an open reading frame consisting of 1995 bp and encoding 664 amino acids too [66]. Trichohyalin (THH) present in the medulla and inner root sheath of hair follicles is the main target for PAD3. The citrullination of trichohyalin by PAD3 causes the strengthening of the inner root sheath and induces hair growth [33, 66]. After synthesis, trichohyalin forms insoluble vacuoles that are stabilized by ionic interactions between *α*-helixes [67]. Recent studies indicate that PAD3 catalytic activity is not limited to skin physiology, but its activity is required for stimulating a programmed cell death (apoptosis) in human neuronal stem cells. In addition, the apoptosis-inducing factor- (AIF-) mediated apoptosis and cytoskeletal organization are regulated by PAD3 citrullination [68]. Due to its bundling with cytokeratin filaments, trichohyalin (THH) is considered as an important structural protein and is known to be a keratin filament matrix protein [33, 69]. At high Ca^+2^ concentrations during differentiation of the THH, it is citrullinated and becomes more structurally open due to the loss of its highly organized *α*-helix confirmation [3].

#### 4.2.4. Peptidylarginine Deiminase Type IV (PAD4)

The fourth enzyme, PAD4, is widely distributed in hematopoietic stem cells and immune cells, such as granulocytes, monocytes, macrophages, and natural killer cells. It is also found in the tumor cells originating from the lung, esophagus, breast, and ovary [70, 71]. PAD type 4 can be found in the CNS and is only contained in leukocytes invading the brain [72]. PAD4 is the only type of PAD that can be found in the nucleus [73]. On chromosome 1 (1p36.13), the entire PADI 4 human gene spans about 55,810 kb coding for 663 amino acids and consisting of 16 exons that interfere with 15 introns. Different techniques such as reverse transcription polymerization chain reaction (RT-PCR) and rapid amplification of cDNA ends (RACE) were used to obtain the full length of cDNAs of mouse PAD type IV; the cloned cDNA appeared to have 2287 nucleotide bases, with an open reading frame (ORF) consisting of 2001 bases yielding 74.6 kDa protein [65]. Structurally, PAD4 is a protein that exists as a head-to-tail dimer [18, 74]. Each monomer consists of two N-terminal immunoglobulin- (Ig-) like domains and one C-terminal catalytic domain. One of the N-terminal domains has nine *β*-sheets and is formed by the Ig subdomain (1); the other domain contains 10 *β*-sheets and four short *α*-helices and is formed by the Ig subdomain (2). The C-terminal catalytic domain is arranged as an *α*/*β* propeller fold that is a feature of the deiminase superfamily [18, 75]. The C-terminal catalytic domain is a very preserved area of the PAD4 polypeptide [2]. PAD4 has five calcium-binding sites named Ca1, Ca2, Ca3, Ca4, and Ca5 [18, 74, 76]. Ca1 and Ca2 is sited in the C-terminal catalytic domain resulting in major conformational changes that shift the positions of several residues to be competent for catalysis. Calcium binding also produces large structural alterations in the N-terminus of the protein [18]. PAD4 was found to be involved in gene regulation [77]. The chromatin structure and function have been shown to be regulated by PAD4 through its ability to citrullinate the intracellular proteins, particularly nuclear histones H2A, H3, and H4 [14, 73, 78, 79] as well as nucleophosmin/B23 [77].

PAD4 play a crucial role in cell apoptosis and in the formation of neutrophil extracellular traps (NETs) [80, 81]. PAD4 induces citrullination of the histone tail that results in chromatin decondensation [11]. Tumor protein (p53) is known to regulate the expression of PAD4 [82]. During apoptosis, PAD4 is activated in response to the high intranuclear Ca^+2^ level and this results in nonspecific citrullination of histones and the lowering of their positive charges [73]. It has been proposed that the citrullination of histones affects nucleosome stability, causing nucleosomes to open up and making DNA more exposed for cleaving and fragmenting by nucleases [3]. When neutrophils fight against invading bacteria, they heavily use the citrullination catalyzed by PAD4 to form neutrophil extracellular traps (NETs) and alter the functions of chemokines [83]. Pathologically, PAD4 is distinct from other PADs as it is involved in many autoimmune disorders such as rheumatoid arthritis (RA), multiple sclerosis (MS), systemic lupus erythematosus (SLE), ulcerative colitis (UC), and other processes of inflammation, such as sepsis and thrombosis [17].

#### 4.2.5. Peptidylarginine Deiminase Type VI (PAD6)

PADI type VI is expressed in many tissues such as eggs, ovaries, testis, small intestine, spleen, lung, liver, and skeletal muscle cells, and it has been detected in early embryos. It is essential for the development of the embryo after the second cell stage [41, 42]. Many studies have documented that PAD6 is a maternal gene indeed expressed in preimplantation embryos and oocytes [84]. Using the international human genome NCBI database, the PADI6 gene is localized on 1p36.13, spans 28.8 kb, and consists of 16 exons. In a large-scale cDNA sequence study, the human PADI type VI gene was found to be encoded by 2397 bp of isolated cDNA, and its open reading frame consists of 52 to 2136 bp and encodes 694 amino acids [42]. The specific role of PAD6 is yet unclear. The deiminating functions of the PAD6 enzyme are indicated through its high sequence homology with the other PADs. Protamine is an arginine-rich nuclear protein found in the sperm cells, and this protein could be citrullinated by PAD6 and result in sperm chromatin decondensation [85]. However, during early development; PAD6 could induce the reorganization of the egg cytoplasmic sheath through the citrullination of cytoplasmic components. PAD6 has been proposed to be a target for contraceptive drugs [86].

### 4.3. Abnormality in PAD Function

The PAD activity level and balance are very important in the citrullination process and in their physiological and pathological effects. When PAD activities become uncontrolled, this will lead to abnormal citrullination and, as a consequence, it will lead to disorders. The specific reason underlying PAD dysregulation is not well defined, but there are many factors which may explain abnormal citrullination such as high levels of calcium that may affect PAD target specificity and their activity, unchecked translation of protein arginine deiminases that could cause an increase in citrullination [87], abnormal tumor necrosis factor alpha (TNF-*α*) signaling that is characteristic of ulcerative colitis (UC) and rheumatoid arthritis (RA), and TNF-*α* that can induce the translocation of PAD4 [88]. PADs can citrullinate tumor necrosis factor alpha [89], and autocitrullination of PAD4 may be the producer of faulty levels of citrullinated proteins [90]. However, even though it does not disturb enzyme specificity and activity, it does influence the association with other proteins which is responsible for the posttranslational modification of histones [87]. Besides, the sequence of amino acids and confirmation in the vicinity of arginine residues can also affect sensitivity to citrullination [6, 16].

## 5. Pathological Conditions of Citrullination

The citrullination process is implicated in many human diseases and inflammations that induce autoimmunity responses against citrullinated proteins. Therefore, anticitrullinated protein antibodies (ACPA) are targeting these citrullinated proteins/peptides at specific tissues. Citrullination could involve many proteins, for example, filaggrin, keratin 1, vimentin, myelin basic protein (MBP), glial fibrillary acidic protein (GFAP), fibrin, fibrinogen, *α*-enolase, and collagen II; it can create and expose nonself epitopes that induce autoantibody production [91]. A citrullination process catalyzed by a PAD enzyme plays a crucial role in many inflammatory disorders. These disorders include rheumatoid arthritis (RA), psoriasis, systemic lupus erythromitosis (SLE), and cancers. This process is also associated with neurodegenerative diseases such as Alzheimer's disease (AD) and multiple sclerosis (MS). The citrullination process has been found to be widely presented in inflammatory tissues. It was proposed that this process is considered as an inflammation-dependent rather than a disease-dependent process proving that inflammation is a common ground for multifactorial diseases involving various chronic inflammatory rheumatic diseases [92]. Furthermore, it has been clarified that citrullination is not a specific disease-related case, but it is an inflammation-dependent process existing in diverse inflamed tissues [92], for example, MS, RA, AD, psoriasis, Parkinson's disease, psoriatic arthritis, juvenile idiopathic arthritis, osteoarthritis, spondyloarthropathy, autoimmune hepatitis, Lewy body dementia, and multiple system atrophy [11].

### 5.1. Multiple Sclerosis

Multiple sclerosis (MS) is one of the most widespread neurologic diseases affecting young adults. According to the Multiple Sclerosis International Federation, the worldwide prevalence of the number of people with MS has increased from 2.1 million in 2008 to 2.3 million in 2013, with a woman : man ratio of 2 : 1 [93]. MS is a chronic and progressive demyelinating disease that minimizes nerve cell communication. It is causing numerous neurologic dysfunctions mainly involving the loss of motor function and vision impairment. The relation between abnormal protein citrullination and MS was identified in both human MS patients and animal MS models (i.e., autoimmune encephalomyelitis (EAE)). Myelin basic protein (MBP) is the primary content of the myelin sheath and the main target for PAD-catalyzed citrullination. MBP is highly deiminated up to 3-fold higher than normal [94]. In normal conditions, the amount of citrullinated MBP is around 20%. This percentage increased up to 45% in the chronic stage and will reach up to 90% in the fulminating form of MS (Marburg's variant) [95]. Importantly, autoimmune disorders are influenced by gender, hence the X chromosome in females plays an important role in the superior immune response and might be involved in the breakdown of self-tolerance; therefore, autoimmune disorders such as multiple sclerosis and rheumatoid arthritis are more dominant in women [96].

#### 5.1.1. Myelin Sheath Formation

**Figure 3:**
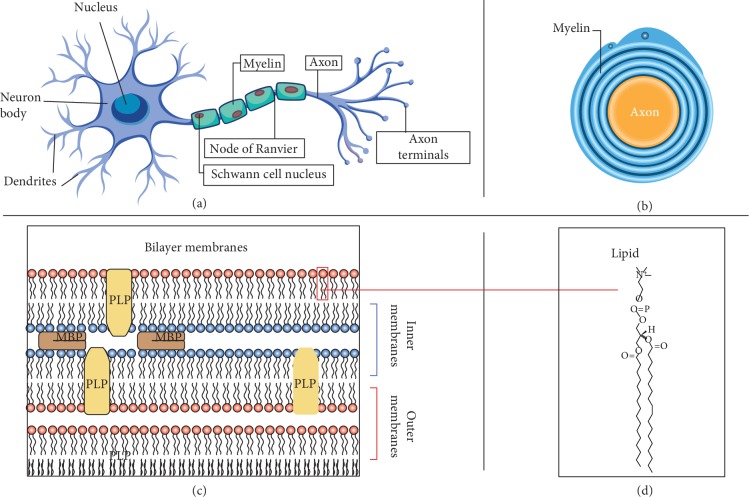
(a) Myelinated nerve fiber is shown with salutatory conduction of action potential. (b) Transverse section of myelinated axon at the internode. (c) Bilayer membranes and with integrated MBP and PLP. (d) Phosphatidylethanolamine (PE).

Myelin sheath formation in the central and peripheral nervous systems is attributed to different glial cell types that form myelin in a different manner. In the peripheral nervous system (PNS), Schwann cells are responsible for forming myelin as each cell forms a single myelin sheath (Figure 3). On the other hand, oligodendrocytes are accountable for making myelin in the central nervous system (CNS), where each oligodendrocyte can make multiple segments of myelin. Despite that there are several morphological and molecular differences between nerve fibers in the PNS and CNS, the basic myelin sheath configuration and the electrophysiological properties are basically the same [97].

The sufficient conduction of nerve impulses between neural cells requires an electrical insulation of the neurons through the myelination of neural cell axons, which is provided in the CNS by oligodendrocytes and by Schwann cells in the peripheral nervous system. On the other hand, demyelination is a process that causes indigence or depletion of nerve signals, which subsequently results in multiple clinical symptoms (e.g., visual loss, extraocular movement diseases, paresthesia, loss of vision and sensation, weakness, dysarthria, spasticity, ataxia, and bladder dysfunction). The myelin sheath is multibilayer structure that surrounds the axons and contains lipid-protein compounds with a ratio of 3 : 1. There are two major proteins detected in this complex, myelin basic protein (MBP) and proteolipid protein (PLP or lipophilin), and they account for 85% of the protein molecules [2]. Compared to other cell membranes, the myelin membrane is highly rich in lipids, containing 75–80% anionic and neutral lipids and around 20–25% proteins [98]. In CNS and PNS, the major lipid categories of myelin are cholesterol, phospholipids, and galactolipids, which support the compact assembly of the membrane [99, 100]. In CNS and PNS, myelin proteins linked tightly with the lipid membrane [101–103]. In the CNS, the major myelin proteins MBP and PLP account for about 30% and 50% of the total protein fraction, respectively [104, 105]. On the other hand, in the PNS, the myelin protein MBP accounts for 5–18% of the total protein fraction, while the rest is composed of P0 glycoprotein, peripheral myelin P2, and the peripheral myelin protein-22 (PMP-22). MBP is considered an important factor contributing to the construction and integration of the CNS myelin and the preservation of myelin stability [106–108]. The classic 18.5-kDa isoform is the most abundant variant of the human MBP protein. MBP appear flat within myelin propounding the opposing bilayers [109]. The high positive charge of MBP interacts with the anionic cytoplasmic membrane surfaces that link the two bilayers at their cytoplasmic sides in a closely reversed manner. Another significant role of MBP is to make a barrier that filters and prevents the cross-passage of the proteins with a high molecular size into the membrane sheath [110, 111].

#### 5.1.2. PADs' Role in MBP Citrullination of Multiple Sclerosis (MS)

**Figure 4:**
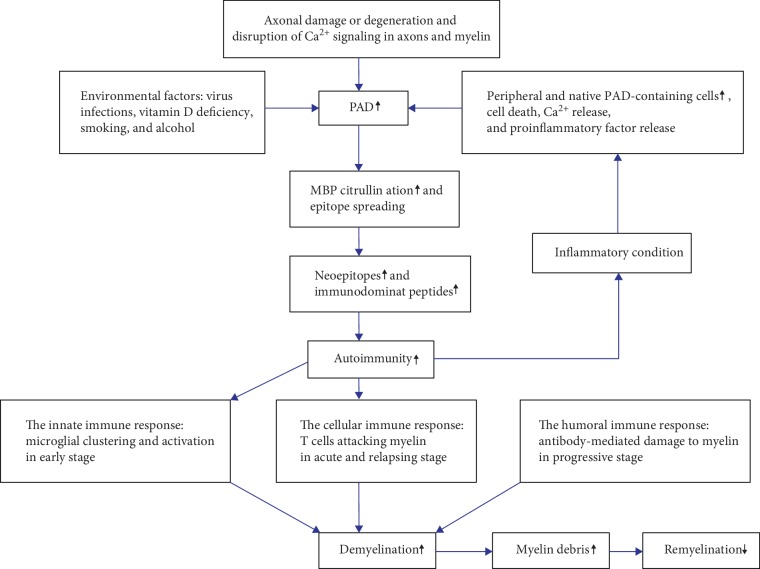
The role of MBP citrullination involved in MS pathogenesis.

In MS, the citrullination of MBP limits its ability to properly associate with lipids [3], which in turn leads to demyelination through the destabilization of the sheath assembly [112]. The amounts of PAD2, PAD4, and citrullinated proteins detected in myelin isolated from patients with MS were elevated when compared to those found in myelin from normal cases. These data indicate that the significant change in the pathogenesis of MS which is associated with the citrullinated proteins results from increased PAD2 and PAD4 activities [113]. The amino acid sequence of human MBP is formed by 170 residues, consisting of 12 lysine, 19 arginine, 2 glutamic acid, and 9 asparagine residues, with a total net charge of +20 at physiological pH and a high isoelectric point (*pl* > 10) [114–116]. In human healthy adults, 6 of the 19 arginine residues of the MPB molecule (MBP-Cit6) are citrullinated which represent about 18% of the total MBP, compared to 45% in patients with chronic MS. In contrast, the citrullinated MBP of patients with Marburg MS has 18 citrulline residues (MBP-Cit18), which represent 90% [95, 117]. Experimentally, PAD2 and PAD4 were detected by electron microscopy in the myelin sheath. From the recombinant forms of PAD2 and PAD4 enzymes used to citrullinate MBP, the results revealed that 18 of the 19 arginine residues intensively displayed in MBP were deiminated by PAD2, while 14 of 19 residues were deiminated by PAD4 [113]. PAD2 is normally distributed in the CNS, while PAD4 is brought to the CNS by macrophage infiltration during disease progression [118]. MS is an inflammatory disorder of the CNS, and it is mainly associated with PAD2 activity associated with MBP citrullination resulting in myelin sheath demyelination and reducing the nerve signal transduction (Figure 4) [34, 119].

Many pathological cases such as hypoxia and excitotoxicity could result in elevated intracellular calcium and subsequently in the activation of the PAD2 enzyme leading to the citrullination or deimination of MBP in the nerve cells [72, 120, 121]. The hypercitrullination of MBP decreases its positive charge, thus resulting in a reduction of its interactions with phospholipids and disruption of the normal structures of the multibilayer myelin sheath. Moreover, the hypercitrullinated MBP can be unfolded, open, and more liable to degradation by protease proteins, such as cathepsin D [52, 122, 123]. Thus, the fragment produced by this protease, Phe44-Phe89 peptide, acts as an immunodominant peptide which could trigger immune response [124]. Immune response involves the infiltration of lymphocytes and other immune cells to the nervous tissue, hence causing inflammation, oxidative stress, and neuron death or myelin sheath damage [3].

#### 5.1.3. Clinical Stages of Multiple Sclerosis (MS)

Depending on clinical symptoms, radiology, and laboratory investigations, such as MRI, CSF, and neurological examination, multiple sclerosis was categorized into four clinical courses: clinically isolated syndrome (CIS), relapsing-remitting multiple sclerosis (RRMS), secondary progressive multiple sclerosis (SPMS), and primary progressive multiple sclerosis (PPMS) (Table 4) [125].

Clinically isolated syndrome (CIS), which was not involved in the first clinical diagnosis [126], is now identified as a preliminary clinical feature of the disease that presents the characteristics of the inflammatory demyelinating disease [127, 128]. CIS and RRMS may either be active or inactive. RRMS is identified by separate episodes of acute neurological deficits and/or deterioration of a specific neurological function (i.e., relapse), followed by partial or complete recovery or remission [126]. RRMS is the most prevalent pattern of MS, representing around 85% of the total cases. It shows an evident relation with sex as its women : men distribution ratio ranged between 2 : 1 and 3 : 1, progressing in young adults above 20 and below 30 years [129]. 10%-15% of the MS cases miss the relapsing stage and achieve primary progressive multiple sclerosis (PPMS) [126]. Progressive MS is an age-related phase, and it is highly associated with elderly patients whose brains are affected as age increases and the patients develop a constant disability [130]. This progression includes two stages; primary progressive multiple sclerosis (PPMS) and secondary progressive multiple sclerosis (SPMS), both of them can be either active or inactive and can be at least clinically evaluated and assisted annually[131]. Recent studies estimate that 25% and 75% of the patients will develop secondary progressive multiple sclerosis (SPMS) within 5 and 15 years of the first diagnosis, respectively [132].

#### 5.1.4. Microglia Drive the Innate Immunity in Early Multiple Sclerosis (MS)

Microglia and macrophages are recognized as main innate immune cells present in MS lesions where they either directly cause neuroinflammatory tissue damage or act together with T and B lymphocytes [133]. Microglial cells are similar to macrophages and constitute about 10-15% of the cells populating the CNS, where they represent the resident innate immune cells of the CNS. In contrast to macrophages which are bone-marrow-derived, microglia originate from the yolk sac and at a certain time during embryogenesis, they populate the CNS before the blood-brain barrier (BBB) is formed and seal the CNS from the periphery [134, 135]. Microglia and macrophages share specific cell surface markers (e.g., CD11b and CD14) and have an identical phagocytic function that enable them to engulf substances like myelin from the surrounding environment [136]. Microglia became functionally and morphologically activated after activation [137]. During their resting state, the microglial fine processes are highly motile and continually screen/clean their surrounding microenvironment [136]. Activated microglia based on injury or infections have been observed in tissue from MS patients, in both white and gray matter [138]. In the absence of lymphocytes and myelin phagocytosis, microglial activation has been detected in early MS lesions [139, 140], indicating that its primary involvement is in innate immunity. Furthermore, the activation of prevalent microglia in the cortical lesions has been detected in the progressive MS phase [141]. The microglial nodules observed in the normal-appearing white matter (NAWM) may represent the earliest stage in the MS lesions [142]. On microscopic inspection, these lesions revealed some reduction in myelin density together with microglial activation. It was originally suggested that such lesions may represent the earliest stages in the formation of plaques called “preactive” lesions [143]. Moreover, the presumption that MS is controlled by an innate immune response within the CNS itself rather than immune cells crossing a penetrated BBB was supported by the presence of an intact blood-brain barrier (BBB) observed in preactive lesions [137]. In other words, the presence of activated and clustered microglia fuels the hypothesis that the first response for MS is neurodegeneration followed by the activation of CNS immune cells [144]. Therefore, microglial cell activation may represent the primary trigger of pathogenesis within the CNS, while (BBB) would be secondarily affected by activated microglia and their secretions (e.g., IL-17 and TNF-*α*) and/or directly by agents that elicit the first microglial response [145]. It is important to note that these lesions are not necessarily involved in early activation but may occur as a result of a spread and continual activation of innate immune cells [146]. The major histocompatibility antigen (MHC) II molecule is highly expressed in activated and clustered microglial cells in normal-appearing white matter (NAWM) of MS and shares in response to deiminated MBP [147, 148]. This indicates that the conversion of MBP-arginine to citrulline by PADs may assist the affinity of MBP fragments to MHC class II molecules of activated microglia [149]. In MS brains, the activated microglial cells can be injurious by developing an inflammatory response by releasing cytokines, chemokines, and free radicals, which might lead to axonal damage, demyelination, and the death of oligodendrocytes. Otherwise, activated microglia produce factors that enhance remyelination and induce neuroprotection [137]. Microglia have an alternative function within the CNS; they can protect neurons in MS patients and prevent axonal damages. Microglial cells can promote neurogenesis by releasing neurotrophic factors such as brain-derived neurotrophic factor (BDNF), insulin-like growth factor-1 (IGF-1), and neurotropic 3 (NT3). Depending on the inflammatory environment in the CNS, microglial cells can downregulate the inflammatory process by the production of anti-inflammatory cytokines such as IL-10 and transforming growth factor-*β* (TGF-*β*) [150, 151]. Microglia and macrophage cells have a critical role in the initial and continued immune responses to myelin antigens. [152]. Similar to macrophages, microglial cells are classified into M1 and M2 microglial cells. M1 microglial cells act as proinflammatory mediators and express cell surface markers such as CD40, CD74, CD86, and CCR7, whereas M2 microglial cells are anti-inflammatory cells expressing mannose receptor CD206 and CCL22 [153, 154]. However, T cell-mediated macrophage activation is essential for inflammatory demyelination in MS [155]. Recent studies suggested that the initiation and progression of MS refer to the innate immune system by stimulating the effector cells (i.e., T and B lymphocytes) [156]; these effector cells, in turn, produce many mediators which trigger and make the innate immune response persist [157].

#### 5.1.5. Adaptive Immune Response in Multiple Sclerosis (MS)

**Figure 5:**
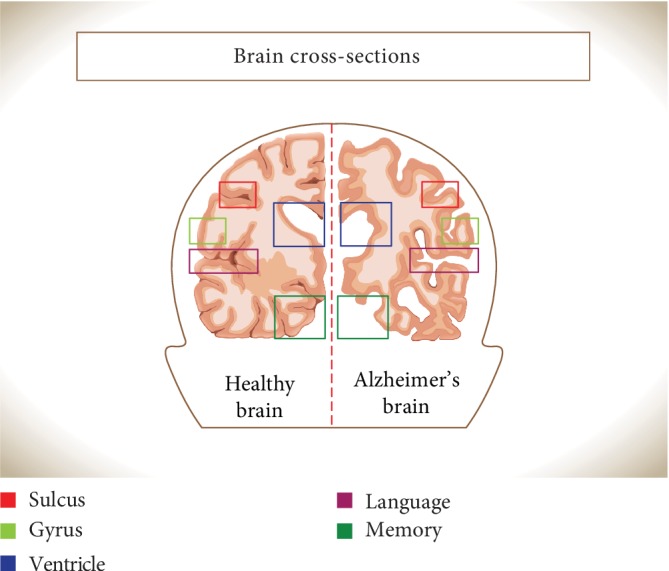
Cross-section of the human brain: normal individual brain (left) and brain from an AD patient (right). Overall shrinkage of brain tissue seen in AD brain with observed expanded sulci and shrinkage of the gyri. In addition, the ventricles seen to be enlarged.

The cleavage and surface exposure of the citrullinated MBP would cause the release of the primary immunodominant epitope of human MBP 85–99, which results in the appearance of autoantigens that trigger immune responses [158]. Due to its high affinity for the MHC class II haplotype, the MBP 85–99 epitope could be recognized by T cells [159]. The complexes of MHC class II with MBP epitopes have been detected on antigen-presenting cells (APC) in MS lesions [147]; therefore, B cells became involved in the presentation of the epitope in a complement-activated pathway [160], so that the autoantibodies and T cell clones can recognize the immunodominant epitope of MBP in MS patients [161, 162]. Recently, newly developed experiments showed that CD8^+^ T cells, which are class I MHC-restricted, could induce brain inflammation and autoimmunity. This is why both class I and II MHC immune response axes are implicated in CNS autoimmunity [163, 164]. Despite this, there is a suggestion that the autoimmunity in MS is more related to the class I MHC-restricted CD8^+^ T cells than it is to the class II-restricted CD4^+^ T cells [143], through the autoreactive Th1 cells which are (not fully) activated peripherally by some unknown mechanisms such as molecular mimicry with infectious agents, super antigens, and/or others. The secreted proinflammatory mediators, including cytokines and chemokines, cause damage to the CNS tissues, leading to multiple inflammatory lesions along the axon in the demyelination of the axon and finally to a neurological deficit [165, 166]. The cells traffic out of the CNS to the lymph nodes for maturation before heading back again to the target organ and induce more damage [166]. These activated T cells will then proliferate, expressing some receptors and adhesion molecules as well as secreting proinflammatory mediators and metalloproteinases that activate the blood-brain barrier (BBB) to interact with it and enter the brain [125]. The antigen-presenting cell, MHC class II, and costimulatory signals (CD28, B-7.1) stimulate the activation and proliferation of previously entered T cells into Th1, Th2, and Th17 cells (Figure 5) [125].

Dendritic cells (DC), astrocytes, and macrophages are the most prominent APCs in the CNS. APCs possess pathogen-recognition receptors (PRR) that can recognize the pathogen-associated molecular patterns (PAMPs) expressed by antigens or pathogens, [167]. DC is very effective in priming and differentiating naive T cells and stimulating antigen-specific effector T cells [167]. Thus, DC secretes IL-12 that induces IFN-*γ*-producing Th1 cells, which contribute to the removal of intracellular pathogens (e.g., virus and bacteria). DC also produces IL-4 and induces the differentiation of IL-4 and IL-5 producing Th2 cells, which are involved in the clearance of extracellular infectious agents (e.g., pathogens and parasites). In addition, DC secretes IL-23 and stimulates the differentiation of IL-17 producing Th17 cells, which are substantial in the immune response against extracellular bacteria and have been involved in autoimmune disorders [168–170]. Proinflammatory cytokines secreted by Th1 cells (IL-2, IFN-*γ*, and TNF-*α*) induce phagocytosis by macrophages and microglia and induce the production of inflammatory mediators (TNF-*α* and NO) and complement factor synthesis. Th2 cell cytokines (IL-4, IL-5, and IL-6) activate B cells to form antimyelin antibodies. Th2 cells also produce IL-4 and IL-10 which suppress Th1 cells [125]. Different studies proposed that inflammation is always present as active demyelination otherwise neurodegeneration occurs, and the extent of demyelination and axonal damage is related to the infiltration of the T cell and B cell even in the cases of patients with PPMS and SPMS [171]. Inflammation in RRMS and progressive multiple sclerosis (PPMS and SPMS) involves the activation of immune cells, mainly microglia and macrophages, MHC I-restricted CD8^+^ cells, and B lymphocytes [171]. Both T cells and B cells are clonally expanded in the NAWM meninges and lesions [172, 173]. The importance of B cells in progressive MS has been confirmed by determining the effect of meningeal ectopic B cell follicles in SPMS patients as well as the effective use of B cell-depleting therapy in PPMS patients [174]. Higher expression of CD80 and CD86 by activating B lymphocyte was observed in MS patients with severe neurodegeneration rather than in MS patients with less neurodegeneration [175].

In MS patients, anticitrullinated MBP peptide antibodies (APCA) were found in samples from the serum and cerebrospinal fluid (CSF) of MS patients [161, 176–179]. This suggests that the maturation of anticitrulline-specific B cells are stimulated by the presence of citrullinated MBP at the site of inflammation in MS. The amount and kinetics of anti-MBP antibody responses are accumulated over time as measured over MS phases. About 12% of the anti-MBP antibodies were estimated in acute multiple sclerosis, which increases up to 32% during disease development in relapsing-remitting and reaches 40% in chronic progressive multiple sclerosis patients [180]. Unlike Th1 and Th2, the Th17 cells have exceptional differentiation factors, including a combination of the immunoregulatory cytokine TGF-*β* as well as the proinflammatory and pleiotropic cytokine IL-6 or IL-23 [181, 182]. Th17 cells are found to have heterogeneous functions, from immune suppressive, regulatory, to inflammatory functions [183]. Th17 cells produce various cytokine groups. There are six subclasses of the IL-17 cytokine group, including IL-17A referred to as IL-17, IL-17B, IL-17C, IL-17D, IL-17E, and IL-17F [184]. IL-21 functions in the regulation of hematopoiesis; NK differentiation; B activation; and T costimulation, T follicular polarization, and survival, while IL-22 plays a role in the inhibition of stimulatory IL-4 produced by Th2 [185]. Most studied groups of cytokines are the IL-17 group. Among all the subclasses, the biological function and regulation of IL-17A and IL-17F are the most identified [186]. These two subclasses (IL-17A and IL-17F) have broad effects on many nonimmune cell types, such as endothelial and epithelial cells. Also, these interleukins could induce local chemokine production to recruit monocytes and neutrophils to the sites of inflammation [181, 187]. Moreover, IL-17F is considered as a central mediator of cellular immunity governing the expression of critical cytokines that exert proinflammatory effects [187].

#### 5.1.6. Risk Factors and Depression Severity of MS in Saudi Arabia

Several studies were carried out among the Saudi MS population considering the risk factor and regional distribution based on hospital-admitted patients as well as clinic visits for both normal individuals and MS patients [188]. The total number of reported MS cases in SA increased from 25/100,000 in 1998 [189] to 40/100,000 in 2008 [190]. While during the 2009 investigation, it showed that the incidence of MS decreased at 30 cases per 100,000 individuals [191]. About 41.1% of the cases were categorized by age 18-29 years with a high occurrence rate in female gender (71.7-75%) [188]. MS family history was found to be a risk factor associated with an increased incidence rate of MS. The risk of MS was 5.8-fold higher within a family with an MS history [188]. The medical history of measles infection was correlated to MS in Saudis [192]. It has been found that an early infection with measles may reduce the risk of MS, while a delayed infection is possibly associated with a high risk of MS. Regarding birth order, first-born children appeared to have about 1.7-fold greater risk than second-born children. Other environmental factors such as diet, sun exposure, and cigarette smoking were evaluated as risk factors increasing the risk of MS in Saudis. The assessment of healthy food consumption revealed that eating fruits and vegetables may reduce the risk and protect against MS [188]. Moreover, fast food and diet with a high level of salt and sodium are linked to the pathogenesis of MS as they stimulate Th17 cells [193]. The association between sun exposure and MS was identified by measuring the serum level of vitamin D; this shows that MS patients are vitamin D deficient and thus sun exposure can reduce the risk of MS [188]. Cigarette smoking contributes to MS, according to the latest statistics from the General Authority for Statistics; about 12.2% of the Saudi population are smokers. Smoking is highly considered as a risk factor in Saudi MS patients, particularly in the male gender. Regarding regional distribution, the central region of SA has a relative prevalence of MS patients more than other regions [189]. About 30.8-24.7% of Saudi MS patients are developing mild to moderate depression, respectively, while about 10.7% seem to have depression. It has been reported that the severity of depression is highly linked to the disability level of MS patients. A significant variation was reported in depression prevalence among Saudi male and female MS patients with high occurrence in females [194]. This matched with other studies that identified the association between depression in MS patients with age and gender [195]. Females were the most suppressed MS patients with a 2-fold increasing risk, and the severity of depression is highly prevalent in younger patients [196]. Among SA regions, MS patients living in the Northern region showed an increased severity of depression; in addition, the severity of depression is proportionally increased with a high educational level and low income [194].

### 5.2. Alzheimer's Disease

Alzheimer's disease (AD) is the most common form of dementia involving millions of people worldwide, especially elderly people. AD is a progressive neurodegenerative disease, causing a growing and irreversible loss of memory and cognitive impairment [197]. Clinically, AD patients are characterized by progressive worsening of cognition, attitudes, and regular activities. Brain atrophy and expanded cerebral ventricles are the major morphological and histological changes in AD patients (Figure 5) [198].

The hippocampus is the main region of the brain which is mostly affected by AD, where a significant citrullination process rate occurs, especially on vimentin and glial fibrillary acidic protein (GFAP) [199]. The formation of senile plaques (SP) and neurofibrillary tangles (NFT) with the presence of cognitive impairment have been defined as the main sign and symptoms of Alzheimer's disease (AD) [200–202]. Different theories were proposed to explain the causes of AD, including the acceleration of aging, degeneration of cholinergic and corticocortical pathways, infectious agents, and immune system dysfunction. Environmental factors such as aluminum exposure, malnutrition, and head injury are linked to AD. Furthermore, genetic factors involving mutations of amyloid precursor protein (APP) and presenilin (PSEN) genes and allelic changes in apolipoprotein E (Apo E) were enumerated as factors causing AD. In addition, vascular factors such as a blood-brain barrier (BBB) disruption and mitochondrial dysfunction like a metabolic disorder have been proposed to cause AD [203].

#### 5.2.1. GFAP and Vimentin Are the Main Proteins in Astrocytes

**Figure 6:**
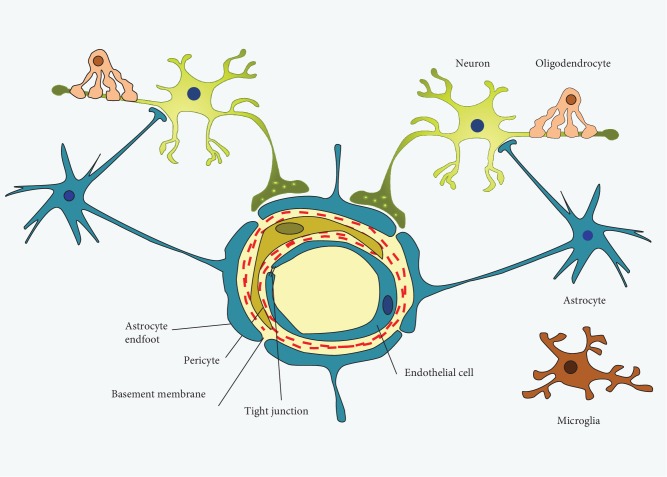
Schematic cross-section of BBB showing the cerebral capillary associated with vascular cells (pericytes and endothelial cells), glial cells (astrocytes), and neurons.

Astrocytes are normal cells in the mammalian brain coinciding with oligodendrocytes and microglia [204]. The main feature of astrocytes is the expression of the intermediate filaments vimentin and glial fibrillary acidic protein (GFAP), which are upregulated during CNS injury, where the astrocytes become reactive in a process known as astrocytosis or astrogliosis [205–207]. The BBB is formed by brain capillary and endothelial cells (ECs) which are strongly associated with astrocyte endfeet processes and pericyte cells [138]. The endfeet of astrocytes build up a connection between the endothelial bloodstream and neurons and regulate the formation and consistency of the BBB (Figure 6) [208]. Astrocytes appear as a common target of PAD2 activation in the pathological conditions as they act as a reservoir of arginine in the brain [209]. Astrocytes composed of an intermediate filament (IF) network are associated with the transduction of biomechanical and molecular signals. Intermediate filament proteins are classified into six categories based upon sequence homology [210]. Proteins such as glial fibrillary acidic protein (GFAP), vimentin, desmin, and peripherin are classified as a type III IF protein. Commonly, GFAP is the main marker for astrocytes and is highly expressed in the aged brain as well as in CNS degeneration and brain injury [211]. The hippocampi of AD patients showed an elevated level of PAD2 and accumulated citrullinated proteins, particularly structural proteins such as vimentin, GFAP, and MBP [3, 212].

#### 5.2.2. Abnormal Accumulation of Citrullinated GFAP in AD

The high citrullination of GFAP and vimentin with the abnormal accumulation of citrullinated peptides has been detected in the hippocampus of patients with Alzheimer's disease (AD). In addition, elevated PAD2 immunoreactivity was also detected in astrocytes of the hippocampus and entorhinal cortex of AD patients compared to age-related normal individuals [199, 213]. It was suggested that citrullination promotes the disassembly process of these intermediate filament proteins [3]. Normally, PAD2 is inactive during neurodegeneration or brain injury when the intracellular calcium balance is disturbed [214]. Experimentally, it was found that the levels of PAD2 in the hippocampus of rats are threefold higher than in the brain cortex. However, under hypoxic conditions and during kainic acid-evoked neurodegeneration, PAD2 is activated and many cerebral proteins undergo citrullination [199]. Interestingly, extracted hippocampal tissues from AD and normal brain revealed that the amount of PAD2 in AD tissue was distinctly higher. In addition, histochemical examination of AD hippocampus revealed the presence of citrullinated proteins obviously in the dentate gyrus and stratum radiatum of CA1 and CA2 areas, while in the normal hippocampus tissue there were no measurable citrullinated proteins [214]. It has been identified that the citrullination of GFAP and vimentin led to their disassembling and unfolding and which subsequently changed the size and shape of astrocytes [199, 215, 216].

An elegant analysis for samples collected from AD and non-AD patients clearly identified the citrullinated sites of three proteins; glial fibrillary acidic protein (GFAP), myelin-based protein (MBP), and neurogranin (NRGN). GFAP appeared to have five citrullinated arginine residues at R30, R36, R270, R406, and R416 compared to MBP that contained fourteen citrullinated arginine residues and one residue in NRGN [217]. The citrullinated GFAP is subjected to caspase 3 which is a cysteine protease that cleaves GFAP and destroys astrocytes in the brain of AD patients [218]. An important role of citrullinated GFAP, which is not conclusively confirmed, is to protect neurons from extrinsic invaders through defects in BBB. Citrullinated GFAP can bind to the deteriorated BBB as the astrocytes' endfeet are tightly attached to the endothelial cell junctions [219].

#### 5.2.3. Pathogenesis and Immune Response in AD

**Figure 7:**
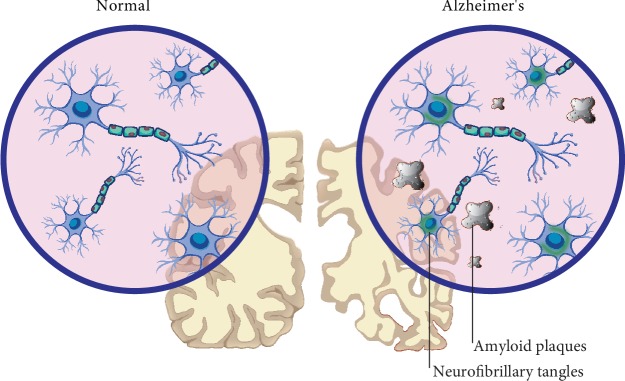
Normal brain (left) and AD brain (right) showing the extracellular (*β*-amyloid plaques) and intracellular (neurofibrillary tangles).

The major neuropathological hallmarks of AD include senile plaques (SP), which are formed by extracellular sedimentation of amyloid *β*-protein (A*β*), intracellular neurofibrillary tangles, which are composed of the tau protein, and deficient neurons and synapses (Figure 7) [220]. Different reports revealed high concentrations of PAD2 and citrullinated GFAP and vimentin in the large reactive astrocytes located around the amyloid plaques [199, 221] and in the frontal cortex at the junction between gray and white matter [216]. These citrullinated proteins are believed to affect the ability of reactive astrocytes in the degradation of extracellular amyloid plaques in AD and phagocytosis of A*β* protein [215, 222–224].

Th cells, including Th1, Th17, and Tregs cells have been indicated to drive the adaptive immune response and to be involved in the early period of AD pathological changes [225–234]. Th1 cell differentiation is promoted by IL-12 and characterized by the secretion of IFN-*γ* and expression of transcription factor T-bet. Th17 cells are highly activated in several autoimmune disorders and chronic inflammation [185, 228, 235–237]. The immune response in AD inflammation involves the differentiation of the Th17 lineage, which is induced by several cytokines, such as IL-12p40, IL-23p19, TGF-*β*, IL-1, and IL-6, and blocked by IFN-*γ* and IL-4. IL-17A and IL-17F represent the main secretions of Th17, which combine to form homodimers or heterodimers. They act with other proinflammatory cytokines to induct neutrophils and monocytes into the inflammation site [184, 238]. The differentiation of Th cells into Treg cells are induced by high levels of TGF-*β* and IL-2. These cells could inhibit deleterious proinflammatory action, e.g., demyelination, and induce myelin regeneration [239]. Thus, the neuronal loss in AD is caused by the loss of cellular components, including citrullinated proteins that trigger the autoimmune response and consequently trigger the production of autoantibodies [221]. Thus, this proves the hypothesis that the accumulation of citrullinated proteins is related to the presence of anti-CCP antibody serum [240, 241].

#### 5.2.4. Prevalence of Alzheimer's Disease in Saudi Arabia

According to the last census and statistic of the Saudi population performed in 2017, the total population is around 32.5 million; the number of elderly people with ages above 65 years is about 1,050,885, which represents about 3.2% of the total population. Among this, 57.48% were male and 42.52% were female. The number of the Saudi elderly is about 854,281 representing 4.19% of the total Saudi population. Out of this number, 48.9% were male and 51.1% were female (Table 6). A survey on the elderly obtained from the General Authorities for Statistics in Saudi Arabia illustrated that 13,343 of the Saudi elderly people are diagnosed with AD, which represent about 1.5%. By age grouping, a high ratio of 78.2% of the elderly people with AD fall in the age above 75 years compared to 2.8% for the age between 65 and 74 years (Table 7) (General Authority for Statistics in the Kingdom of Saudi Arabia (GASTAT)). In addition to aging, other factors lead to high developments of AD such as diabetes and hypertension that are increased in Saudi Arabia with more than 8.5% of the Saudi population.

The most common complications related to Alzheimer's disease are misplaced possessions, falling down, broken bones, and pulmonary infections such as pneumonia. Men were markedly observed to be associated with misplacing possessions and low background for pneumonia; these conditions were reported as the main cause for hospital admission. The attention given to these factors is very important for an AD patient to prevent such complications and to help in early therapy to minimize the level of morbidity and mortality [242].

### 5.3. Systemic Lupus Erythematosus (SLE)

Lupus erythematosus (LE) is a highly heterogeneous autoimmune disease characterized by abnormal immune cells and the production of numerous autoantibodies. LE is classified into two categories, cutaneous lupus erythematosus (CLE) and systemic lupus erythematosus (SLE). The cutaneous pattern (CLE) is characterized by skin wounds or lesions, while SLE is associated with greater systemic impairments [243]. Systemic lupus erythematosus (SLE) is predominantly found in young females especially in the childbearing age group. It involves many organs and tissues of the body, such as dermal tissues; cardiovascular organs; and connective tissue, muscle, joints, brain, and kidney. This disease is accompanied by the release of multiple autoantibodies [244]. Systemic lupus erythematosus (SLE) causes serious morbidity and early mortality, especially in women of childbearing age and minorities. The chronic inflammation and production of pathogenic autoantibodies in SLE are referred to as immune dysregulation, which result in a wide range of clinical aspects, including arthritis, skin rashes, renal failure, and central nervous system damage [245]. The most common complication of SLE that has high morbidity and mortality is lupus nephritis (LN); mortality occurs in end-stage renal disease (ESRD) [246]. The etiology of LN is complex and is attributed to environmental and genetic factors [247].

#### 5.3.1. Source of Antigens Involved in SLE

Apoptosis and NETosis have been defined as the main source of autoantigens in SLE [248]. The impaired or defective mechanism of these processes has been found to produce multiple molecules that contribute to the pathogenesis and autoimmunity of many autoimmune diseases including SLE [249]. Apoptosis is an immunologically silent cell death process with greater regulation that has a critical function in tissue homeostasis. This process is extremely organized and has a safe mechanism for tissues to rebuild on avoiding inflammation and immune response. Regarding apoptosis, the cysteine protease enzyme named caspase is activated to drive the degradation of cellular components in a very regulated and managed pathway. The defect of substantial survival signals and the binding of cell surface receptors such as Fas and TNFR could induce apoptosis. This process involves a sequence of morphological changes, such as cytoskeletal disorganization, cell contraction, DNA segmentation, and plasma membrane protrusion or blebbing [250]. Under normal conditions, the removal of the apoptotic products is the function of immune cells such as neutrophils, macrophages, and dendritic cells. The defective clearance function of these cells with an abnormal apoptosis pathway leads to the accumulation and exposure of the apoptotic debris to the immune system, thus inducing the pathogenesis of SLE. In SLE, the apoptotic blebs have been concentrated with targeted nuclear autoantigens [251, 252]. NETosis is a unique model of neutrophil cell death, and it has been identified as additional source of autoantigens in SLE [253]. During this process, neutrophils emerge from fibrous networks containing DNA fragments, citrullinated histones, and granule enzymes such as neutrophil elastase, myeloperoxidase, and cathepsin G. These components are known as neutrophil extracellular traps (NETs) and serve to catch and kill extracellular pathogens such as bacteria, viruses, fungi, and parasites [254–256]. One of the characteristic features of SLE patients is the impairment of the NETosis process that causes the formation of low-density granulocytes (LDGs). LDGs are neutrophils with an impaired phagocytic function and act as a proinflammatory cell in SLE to enhance the production of cytokines, especially interferon type I (IFN-I) [249, 257]. Apoptosis is a well-organized and controlled process, while NETosis is faster and less well ordered [258]. Insufficient removal of apoptotic cells and NETs by phagocytic cells results in the accumulation and presentation of modified proteins (histones) to the immune cells. These modified histones are detected by innate immune cell receptors such as toll-like receptors (TLR) as autoantigens or foreign substances [258].

#### 5.3.2. Citrullination and Carbamoylation Are PTMs Implicated in SLE Development

**Figure 8:**
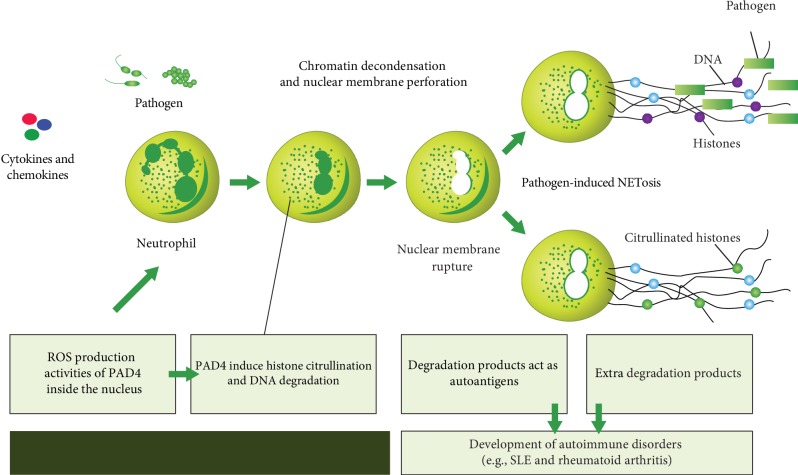
Neutrophil extracellular traps (NETs).

Citrullinated proteins were detected in inflamed tissues of SLE patients, including NET-associated histone protein [71]. A citrullinated histone is defined as a special marker for NETs, and it is important for NET formation (Figure 8). A histone is citrullinated by the catalytic activity of PAD4 during NETosis, where the calcium level is elevated [77, 80]. Certain modifications of NET histones may associate with the tolerance disruption of the NET-associated proteins. PAD4-induced citrullination of histones plays an important role in chromatin decondensation and controlling gene regulation and may contribute to tumorigenesis [71]. In addition to neutrophil extracellular traps (NETs), it has been associated with early and various inflammatory [259, 260] and autoimmune diseases such as SLE [261]. The PAD4 enzyme is predominant in neutrophils and plays an important role in the initiation of NETosis through histone citrullination. Curiously, histones were reported to be extensively disordered, with intrinsic disorder not only being abundant in these proteins but also being crucial for their various functions, starting from heterodimerization to the formation of higher order oligomers, to interactions with DNA and other proteins, and to posttranslational modifications [262]. More detailed information on the roles of PADs and citrullination in NETosis mechanisms of formation, regulation, and in vivo/in vitro induction has been discussed in several dedicated reports [27, 249, 263–270].

On the other hand, carbamoylation is a PTM that is very similar to citrullination; this protein modification is a nonenzymatic process that involves the binding of the isocyanic acid (cyanate) to the lysine residue resulting in the formation of homocitrulline or carbamylated protein (Figure 1). Cyanate is mainly generated from urea dissociation; it may also be produced from the catabolism of thiocyanate [271]. Myeloperoxidase (MPO), which is abundant in neutrophils, may also induce carbamylation in smokers by converting thiocyanate into cyanate [272]. Studies on SLE patients with articular and renal involvement have identified many patients who express autoantibodies against carbamylated protein; these autoantibodies are termed anticarbamylated (anti-CarP) autoantibodies [273, 274]. Recently, both modified proteins, i.e., citrullinated and carbamylated proteins, have been shown to play an important role in the pathogenesis of SLE, the autoantibody systems, including anti-Carp and anti-CCP antibodies which are emerging as useful biomarkers for the diagnosis of the disease [275].

#### 5.3.3. Autoantibodies Produced in SLE

SLE is a systemic autoimmune disorder characterized by the presence of pathogenic autoantibodies that attack many self-antigens. Circulating autoantibodies have been detected serologically and their levels correlated with the SLE severity (Table 8). These autoantibodies are targeting many organs such as the kidneys and skin, causing tissue inflammation and organ disorder. Two isotypes of antinuclear antibodies (ANA), IgG ANA and IgM ANA, have been described to play a significant role in SLE as well as other systemic autoimmune diseases. ANA IgG is involved in disease pathogenesis [276, 277], while ANA IgM is known to prevent autoimmunity and inhibit immune responses induced by IgG ANA; hence, in healthy individuals, circulating ANA IgM is detected and is required for the noninflammatory removal of cellular debris [278–280]. The role of IgA and IgE isotypes in SLE pathogenesis is not well defined [281]. Antinuclear antibodies are detected in more than 95% of the patients. Anti-double-stranded DNA (ds-DNA) and anti-Smith (anti-Sm) antibodies are specific to SLE patients and used for the classification criteria of SLE [277]. The Sm antigen is a small nuclear ribonucleoprotein (snRNP) associated with the RNA molecules. Anti-Sm antibodies attack the snRNP core proteins, whereas anti-ds-DNA antibodies react with the conserved nucleic acid determinant of DNA [282].

Anticitrullinated antibodies (ACPA) or anticyclic citrullinated antibodies (Anti-CCP) are autoantibodies produced against the citrullinated peptides. Some early reports classified these autoantibodies as sensitive, specific, and unique for rheumatoid arthritis (RA) [283]. While later, they were detected in other non-RA autoimmune disorders such as psoriatic arthritis, SLE, and juvenile idiopathic arthritis with majority of their presence detected in RA [284]. Anticyclic citrullinated antibodies (anti-CCP), rheumatoid factor (RF), and C-reactive protein (CRP) are considered as diagnostic markers in the laboratory investigations of SLE, especially the articular subset. Anti-CCP antibodies and RF markedly increased in SLE patients with the articular arthritis, particularly in erosive and deforming episodes [285].

For an extended period, synovitis and musculoskeletal inflammation were the most common manifestations in 69-95% of SLE patients [286, 287]. The identification of erosion or erosive arthritis in SLE was very rare. Recently, the application of more advanced radiological methods such as ultrasonography (US) has assessed the evaluation of the joint damage in SLE, and up to 25.8-71% of the investigated cases have been reported with erosive arthritis [275, 288]. Anti-CCP antibodies have been widely investigated and detected in the sera of erosive SLE patients, and they have been used as a sign of the severity of erosive arthritis in association with US findings and laboratory investigations [287, 289]. In the last few years, different studies were conducted on small and large cohorts to evaluate the presence of anti-CCP antibodies in the US-confirmed SLE with erosive arthritis. The findings of these studies showed variable levels of anti-CCP, ranging from 12% in some studies up to 50% in another [287, 288, 290].

More recently, the anticarbamylated protein (anti-CarP) antibodies have been proposed as a candidate biomarker for erosive arthritis in SLE patients. Recent studies have revealed the prevalence of the anti-CarP in SLE with joints and renal involvements [274]. The evaluation of anti-CarP antibodies in SLE patients with kidney involvements showed a significant incidence (27.5%) of anticarbamylated vimentin autoantibodies in the lupus nephritis phenotype, thus suggesting that the carbamylated proteins (vimentin) trigger autoimmunity in SLE, and anti-CarP antibodies could be a useful biomarker in SLE with renal involvement [291].

Moreover, the association between anti-CarP antibodies and SLE has recently been assessed in large cohort studies of SLE patients with and without articular involvement; the findings showed a 50% prevalence of anti-CarP antibodies in SLE with joint involvement, which is higher than in patients without joint involvement [292]. A few recent studies have been considered with the anti-CarP antibodies and their significance in SLE with erosive phenotypes. The prevalence of anti-CarP antibodies was up to 28% in SLE patients with radiographically detected erosive damage. The significant association between anti-CarP antibodies and erosive arthritis suggests that these antibodies provide a new biomarker for SLE, particularly for SLE with erosive joint damage [274].

#### 5.3.4. Immune Dysregulation Involvement in SLE

Role exploration of immune response dysregulation in the pathogenesis of early SLE was the basis to reach the ideal avenue for preventing the progression of organ damage and reducing the morbidity and mortality associated with early SLE [293]. Macrophage and dendrocyte cells are accountable for the recognition and clearance of the products of the apoptotic process by their function as phagocytic cells; this process is very complicated and driven by different factors. The defect in this function results in the deposition of dead cells and fragments which trigger the immune response. Phagocytosis is a well-organized serial process involving the recognition and opsonization of apoptotic cells and debris, secretion of chemokines, and engulfment of these apoptotic cells. Therefore, the efficiency of phagocytosis may induce (proinflammatory) or dampen (anti-inflammatory) the immune response [248]. Pathogenesis of SLE is also attributed to an abnormality of an adaptive immune system involving functional defects of T and B lymphocytes and abnormal secretion of cytokines. Moreover, aberrant secretions of cytokines were revealed to give an early prognosis in SLE; this is confirmed by detecting the early presence of these cytokines before the production of autoantibodies [293–299]. Moreover, it is presumed that the pathogenesis of early (preclinical) stages of SLE are affected by the dysregulation of CD4^+^ or T-helper lymphocytes (Th1) associated with the abnormal secretion of cytokines, especially interleukin 2 (IL-2). Likewise, the irregular secretion of interleukin 17 (IL-17) by T-helper lymphocytes (Th17) induces high inflammation response [295]. It has been shown to play an important role in autoimmune diseases such as systemic lupus, leading to dysfunction and inappropriate regulation associated with disease activity. The number of TH17 lymphocytes increased in systemic lupus patients, and in contrast, samples lacking TH17 were resistant to the disease. Because of this, its impact is known while its generation mechanism is not entirely defined [300, 301]. Recently, studies have provided further evidence that the IL-21/IL-21R pathway plays a major role in the pathogenesis of autoimmune diseases, in particular SLE. The production of IL-21 is mainly restricted to CD4^+^ T cells, Th17-, and T follicular helper (TFH) lymphocytes.

Plasma levels of IL-21 were significantly elevated in SLE patients in comparison with healthy controls. The IL-21 receptor (IL-21Ra) could be expressed in various cell types, including T-, B-, and NK lymphocytes; dendritic cells; and macrophages. IL-23 is a heterodimeric cytokine produced predominantly by activating antigen-presenting cells, such as macrophages and dendritic cells. Although IL-23 plays minor role in the differentiation of Th17 from naïve T cells, it is necessary for driving the expansion of Th17 cells and so it is involved in the pathogenesis of various autoimmune diseases. This is why the IL-23/IL-17 axis is one of main cytokine axes driving the pathogenesis of various autoimmune diseases. IL-23 and IL-17 levels as well as the number of Th17 cells were elevated in SLE patients compared to control subjects regardless of disease severity [302]. In some studies, the levels of circulating IL-17 correlated with disease activity. Also, such cytokine levels were even higher in lupus patients with nephritis than in those without nephritis [300]. Although SLE patients have increased IL-17 production, the role of this cytokine in lupus pathogenesis is yet to be defined. IL-17 can induce the production of an array of inflammatory cytokines, chemokines, and MMP from immune and nonimmune cells, leading to the recruitment and activation of inflammatory cells with tissue damage .We suggest that IL-17 could be responsible for inflammatory tissue damage in lupus nephritis because of the increased IL-17 gene expression and excretion in the urine sediments from lupus patients [302].

In addition, the dysregulation of the cytokine pathway was suggested to play a significant role in the pathogenesis of early stages of SLE. Abnormal interferon (INF) and interleukin (IL) pathways with marked elevations of IL-6, IL-5, and IFN-gamma (IFN-*γ*) have been detected in SLE patients compared to normal individuals and preceding the autoantibody production by more than 3.5 years. Similarly, different chemokines and tumor necrosis factor (TNF) superfamily proteins such as proliferation-inducing ligand (APRIL) and B lymphocyte stimulator (BLyS) were elevated lengthwise in SLE 10 months before being diagnosed [293].

It has been observed that all patients with lupus erythematosus had an increase in the expression of interferon type I (IFN-I). This may relate to TH17, where higher rates of IL-17A, IL-17F, and type 1 IFN were found in patients with lupus erythematosus when compared to patients who did not carry it and to healthy individuals. This indicates a correlation activity between IFN- and TH17-specific IL-17 cells, which release IL-21 causing SLE [181, 302]. A strong inverse correlation exists between serum IL-27 levels and SLE disease activity. This finding suggests the existence of signals that inhibit IFN-I-mediated IL-27 production to enable the generation of pathogenic Th17 cells. IFN-I-induced IL-27 will suppress the Th17 tissue-damaging response which may prevent undesirable immunopathology. This immunoregulatory pathway is disrupted in the SLE case and other IFN-I-dominated autoinflammatory conditions, where the chronic activation of C5a will suppress the IFN-I induction of IL-27 production and permit the generation of pathogenic Th17 cells. This results in the breakdown of tolerance and subsequently end-organ damage. C5a could also modulate adaptive immune responses, including T cell proliferation/differentiation, and could also modulate the balance of the Th17 cell subsets. A recent report indicated that the C5a-C5aR interaction inhibited Th17 differentiation through diminished production of TGF-*β*, IL-6, and IL-23. C5a enhanced IL-17 production by human T cells, which is dependent on IL-6 enhancement. It demonstrated the negative regulation of IFN-I-induced IL-27 production by the complement component C5a via the C5aR receptor on macrophages. Activation effects of C5aR on the macrophage blocked IFN-I-mediated IL-27 production and permitted the differentiation of Th17 cells. We found that C5a inhibited IFN-I-induced IL-27 production, and the level of serum C5a correlated with Th17 frequency in the peripheral blood [302]. Many studies suggested that a strategy designed to reduce the levels of C5a by neutralizing it or preventing its cleavage from C5 and/or strategies to downregulate C5aR expression or signaling may have beneficial effects for SLE patients. Several therapeutic agents have been developed to block the effects of C5a. Eculizumab is one such therapy which prevents C5 from being cleaved to form C5a. It is currently approved by FDA for use in paroxysmal nocturnal hemoglobinuria [302].

Production of both IL-5 (Th2) and IL-6 (Th2 and Th17) cells could be fundamental in the early pathogenicity of SLE. About 20% of preclassified SLE patients showed an elevated amount of IL-5 and IL-6 six years before classification compared to 90% of two-year postclassified patients [293]. A previous study has suggested that the TLR/ILR family and the single immunoglobulin IL-1-related receptor (SIGIRR) controls the differentiation of Th17 cells and the secretion of IL-17 [300]. The results of studies demonstrate that SIGIRR and Th17 cells are the key elements in the control of chronic autoimmune diseases, including SLE. Recent studies also have pointed out other agents that could affect the differentiation of TH17 [303], such as increasing the amount of sodium chloride in the foodstuff and thereby enhancing the response to autoimmune diseases. This is similar to leptin, which accelerates the development of red lupus autoimmune diseases and demonstrates its ability to promote cell differentiation TH17 [50].

**Figure 9:**
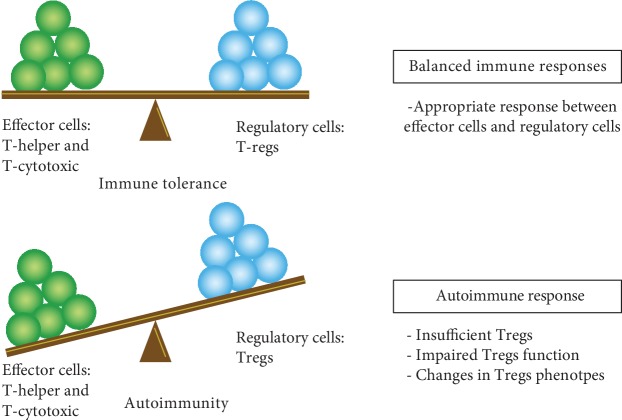
Immune dysregulation in SLE. Above: balanced immune tolerance between T-effector cells and Tregs. Below: insufficiency and deficiency of Tregs result in autoimmunity.

T suppressor or regulator cells (Tregs) are lymphocytic cells characterized by the expression of CD4^+^ and CD25^+^ and are known to have an essential function in the preservation of immune tolerance (Figure 9). The impaired function of Tregs results in the abnormalities of B and T cells and SLE progression [304]. The regulatory function of Tregs depends on the secretion of inhibitory cytokines such as IL-10, IL-35, and TGF-*β*and the secretion of cytotoxic toxins such as perforin and granzymes. IL-12 produced by dendritic cells is important for T-lymphocyte activation and Th1 lineage differentiation. This cytokine is blocked by IL-10 of Tregs, thus Tregs regulate the immune response induced by T cells. In addition, dysregulation of Tregs like in the defect of IL-10 secretion is known to cause mucosal inflammation without evoking an autoimmune response [305–308]. Recently, Treg lymphocytes revealed a reversed role in the pathogenicity of SLE via their elevated level in the active stages compared to inactive patients and normal individuals [309–312].

#### 5.3.5. SLE in Saudi Population: Prevalence and Awareness

Many studies that were performed at the Eastern and Central regions of Saudi Arabia showed that SLE is more distributed in young females of childbearing age between 20 and 30 years [313–315]. Genetic factors seem to be the major reasons for the pathogenesis of SLE. Consanguineous families with positive SLE showed of autosomal recessive inheritance. The MHC class II gene is highly associated with the occurrence of SLE in the Saudi population. The HLA-DRB1^∗^ 15-HLA-DQB1^∗^06 haplotype is considered a major risk factor for SLE in Saudis [314]. Most causes of hospital admission in Saudi Arabia are due to active SLE disease followed by renal diseases. Concerning the gender, Saudi males are associated with a poorer prognosis [316]. Regarding childhood SLE, girls are five times more susceptible than boys before adulthood. In the adolescent age, women are more affected than men at a ratio of 14 to 1, which is similar to that in adult patients with SLE. Various studies have found that the most evident symptoms were reported after puberty at the adolescent age, and these mainly involve hematological and renal manifestations with an offensive course [317, 318]. Depression is the most common psychiatric disorder in Saudi SLE patients, and this would be related to the cultural and social differences regarding the acceptance of chronic illness as well as the unique lifestyle practices by women in Saudi Arabia that can substantially increase the pressure on patients, particularly females, and thus impact their mental health. The high prevalence of depression among Saudi patients with SLE may highlight the need for routine and careful evaluation for depressive symptoms among those patients. Adequate psychiatric consultation and appropriate treatment are necessary for SLE patients [319]. Other studies were performed to assist and support the awareness of Saudis about SLE, and significant reports showed poor public knowledge of SLE among participants even among medical students [320].

### 5.4. Rheumatoid Arthritis (RA)

Rheumatoid arthritis (RA) is a systemic disorder of indefinite causes that is characterized by inflammation, chronic pain, and joint deformity (polyarthritis) which extend from distal to proximal joints. RA is manifested by serological testing of autoantibodies [321]. It is an inflammatory immune-mediated disease with a prevalent ratio of 0.5–1% in developed countries [322, 323]. Chronic synovial inflammation and hyperplasia represent the main characteristics of RA which drive the particular damage and bone thinning which leads to functional impairment and disability [324]. Several studies found that RA is globally widespread with no area or racial specification (Table 10) [321], and its annual rate for occurrence is around 40 cases per 100,000 with an obvious incidence ratio (2 : 1 to 3 : 1) between females and males [325–327]. The lifetime risks of RA patients estimated among females and males were found to be 3.6% and 1.7%, respectively [328]. Rheumatoid arthritis is one of the autoimmune disorders characterized by the production of autoantibodies such as rheumatoid factor (RF) anticitrullinated peptide antibodies (ACPAs). Noteworthy, these autoantibodies were significant markers in the diagnosis of RA, and some patients showed negative serology. This disease is complicated and could occur in response to different factors such as environmental and genetic factors. Insufficient therapy of RA can cause joint destruction and irreversible disability [329].

#### 5.4.1. Risk Factors in RA Development

RA is considered a prototypic polygenic disorder in which the combination of genetic and environmental factors results in the development and production of autoantibodies that invade and destroy self-tissues [330]. The locus HLA-DR4, particularly DRB1^∗^0401 and DRB1^∗^0404 alleles, is considered as a major genetic risk factor in RA and contributes to about 40% of genetic impact. About 70% of RA patients are identified to express HLA-DR4 (DRB1^∗^0401 and DRB1^∗^0404), compared to 30% expressed in normal individuals [331]. These alleles encode for a specific amino acid sequence known as the shared epitope (SE) [332]. The shared epitope (SE) or “susceptibility epitope” is composed of 70 to 74 amino acids located in the third hypervariable region of the DRB series. The risk of these alleles in RA development is clarified through their ability to submit an arthritogenic substance, especially deiminated or citrullinated proteins which is subsequently recognized by T lymphocytes [331]. The environmental risk factors for RA involve cigarette smoking, silica exposure, changes in the microbiota, infectious agents, vitamin D deficiency, and obesity. Although some of these factors were studied, not all are supported by clear evidences. The association between genetic and environmental factors is believed to play a crucial role in RA progression.

The production of ACPAs is highly linked to the shared epitope and cigarette smoking. Smoking is considered as the major environmental risk factor contributing to RA disease. Furthermore, it can trigger both innate and adaptive immune responses [333]. In contrast to the nonsmoking and nonexpressing (HLA-DR4 SE) individuals, the susceptibility for RA is 20 times higher in patients with associated smoking and SE [334]. In an interesting report performed on a group of first degree relatives (FDR) of the RA patients in this study, cigarette smoking was found to be a significant and potentially adjustable risk factor during preclinical stages of RA. In this FDR group, a patient less than 50 years old and smoking more than 10 packs per year appeared a high risk for RA and joint inflammation [335]. The exposure to certain chemical dusts such as silica, cement, and glass filaments leads to an increased risk of RA, particularly silica exposure. A study was performed on a firefighter and an emergency helper who participated in the rescue during the September 11 attacks; later these responders showed high susceptibility for developing autoimmune disorders, including RA [336, 337]. In addition, exposure to cloth dust contributed to a higher risk for RA between Malaysian women [338]. The variation in the composition and function of gut microbiota is tightly linked to RA. Other studies have shown a modified gut microbiota (dysbiosis) in RA cases. Stool samples obtained from RA patients were analyzed for gut microbiota; the results showed a reduction of gut microbiota in comparison to normal individuals which correlated with disease period [333, 339]. Periodontitis pathology, similar to RA, is characterized by chronic progressive inflammation that causes bone dissolution in the mouth cavity. The source of this disease is *Porphyromonas gingivalis* which targets individuals predominately expressing HLA-DRB1^∗^04 alleles [82]. Several epidemiological reports illustrate the prevalence of RA among periodontitis patients [340]. The pathogenesis of RA related to the *P. gingivalis* bacterium, which is capable of stimulating citrullination under PAD activity [333], and antibodies produced against this bacterium are correlated with ACPA titers [341].

#### 5.4.2. RA and Citrullination

The existence of citrullinated proteins is not only a unique feature of RA but its capability of eliciting the autoimmune response to this posttranslational modification is a very important diagnostic feature [330]. Although many efforts and experiments concentrate on certain modified proteins such as vimentin, enolase, antithrombin, and type II collagen, the later researches on RA patients showed reactivity against a wide variety of citrullinated proteins [342]. The presence of autoantibodies to natural citrullinated peptides has been considered as a highly distinctive and positive predictive value (PPV) for RA cases [343]. The elevated level and the translation and activation of PAD2 and PAD4 enzymes are clearly present in RA patients [3, 83]. These enzymes are implicated in the inflammation process of RA. The PAD4 enzyme is mainly located in phagocytic cells such as macrophages and granulocytes such as neutrophils and eosinophils, whereas PAD2 is highly distributed in macrophages [55]. The inflamed synovial tissues in RA also have many PAD2-presenting macrophages and occasionally PAD4-expressing granulocytes. Usually, PADs are located intracellularly, either in the cytoplasm or in the nucleus specifically for PAD4 [2].

RA patients revealed high titers of ACPA in both serum and synovium, which correlated to the intracellular citrullinated peptides independent of local disease activity. The prevalence of intracellular citrullinated proteins was accompanied with PAD2 [344], whereas the extracellular citrullinated peptides, especially fibrin, were found to be related to PAD4 [345]. Cytoplasmic PAD2 and intranuclear PAD4 are found in an inactive condition within cells due to the low level of intracellular calcium. [346]. As Ca^2+^ concentration rose, both PADs were activated and leaked out to the cells to catalyze extracellular protein citrullination [16, 55]. Citrullination or deimination has been found to affect the coagulation factors such as fibrin proteins, which can be citrullinated by PAD2 and PAD4 and can stimulate an immune response at the affected sites. RF and ACPAs then target these citrullinated fibrinogens [347]. In this context, antithrombin, which is a thrombin inhibitor, is known to be deiminated by PAD4, and citrullination of antithrombin diminishes its function to prevent thrombin formation which results in a high rate of clotting or coagulation and consequently may cause RA. Therefore, antithrombin is important in RA stimulation, mainly at the beginning of the disease [348]. Citrullinated fibrinogens were detected early in the synovium of RA patients [349] and were found to be targeted by ACPA [346]. The coagulation process is balanced by the fibrin clot formation and degradation (fibrinolysis). Fibrinogen is converted to fibrin by the action of thrombin into *α* and *β* chains of fibrin. Citrullinated fibrinogen affects the balance between coagulation and fibrinolysis and causes the accumulation (deposition) of fibrin. These fibrin deposits induce autoimmunity and are defined to be the pathological hallmark of RA. The major antigenic targets for ACPA in the rheumatoid synovium were the *α* and *β* chains of citrullinated fibrin [349]. The pathogenicity of RA was attributed to collagen, and the citrullinated collagen type II was found to increase the susceptibility for autoimmune response and inflammatory arthritis. Moreover, the risks of arthritis were linked to the amount of activated PAD4 and deiminated collagen [3].

#### 5.4.3. RA Citrullinome

Citrullination is an important physiological process when it occurs in selective patterns such as citrullination of filaggrin during keratinocyte differentiation, citrullination of MBP for CNS plasticity, and gene regulation [350, 351]. The pathological or nonselective citrullination occurs as a result of dysregulated PAD activity, which leads to the production of new citrullinated epitopes not tolerated by the immune system and subsequently drives the immunogenicity in predisposed individuals [352].

The synovial fluid of RA patients has been found to comprise a unique type of citrullination known as hypercitrullination. The poteomic analysis of the RA synovium has specified more than 150 citrullinated proteins in the cellular and extracellular components, which encompass the RA citrullinome [351–354]. This citrullinome had been identified to play an essential role in the pathogenesis of RA and the generation of ACPAs. Although a large number of citrullinated proteins have been detected in the RA synovium, specific proteins, e.g., vimentin, fibrinogen, and *α*-enolase, have been identified as the primary targets for ACPAs [352]. Recently, the investigations of the several synovial fluids from RA patients lead to the discovery of complete new citrullinated proteins that were unspecified as a part of an RA-associated citrullinome [354].

As aforementioned, PADs are catalytic enzymes promoting protein citrullination, and an abundance of PAD-expressing cells exist in RA joints, which contributes to the RA citrullinome. Neutrophils, monocytes, and fibroblast-like synoviocytes (FLS) are the most abundant cells in the synovium and have been considered as major sources of PADs involved in the intracellular and extracellular citrullinations [355, 356].

#### 5.4.4. Immune Response and Autoantibodies in RA

**Figure 10:**
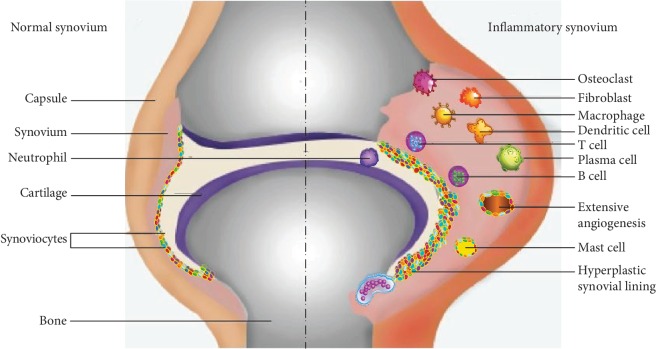
Schematic view of (a) normal joint and (b) RA joint.

The histological structures of the synovial joint in both normal and RA inflamed sites include the synovial joint surrounded by outer fibrous capsules which consist of the synovial cavity filled with synovial fluid and surrounded by synovial membrane. The synovial membrane composed of the cellular intima layer is characterized by two synoviocytes cells, the macrophage-like synoviocytic cells and fibroblast-like synoviocytes (FLS). The FLS is responsible for the secretion of the synovial fluid which has a role in the immunity of RA. The vascular subintima is a loosely connective tissue containing fibroblasts, macrophages, mast cells, and fat cells (Figure 10).

The contact between structural cells and innate immune cells within synovial joints is observed and involved during RA development. The innate immune cells are very close to synovial cells and they have an important role in RA progression. In the RA synovium, fibroblast-like synoviocytes (FLS) are also implicated in the joint damage. RA FLS was found to be activated by cytokines such as TNF-*α* and IL-6 through cell-to-cell contacts or through the activation of TLR2, TLR4, and TLR3. After the activation of the FLS, cells attack the extracellular matrix (ECM) and secrete matrix metalloproteinases (MMPs) which are implicated in destroying the cartilage and bone tissues leading to the amplification of joint destruction. During chronic inflammation, the FLS cell invades the noninflamed synovial tissue, causing an expansion of arthritis and the damage of the other joints [357]. These cells secrete MMPs and proinflammatory cytokines by the action of TGF-*β* [333].

The macrophage-like synoviocytes secrete many cytokines, such as IL-1, IL-6, and TNF which are involved in the proinflammatory response [358]. The tissue-residing mast cells play many significant roles in several processes such as inflammation, fibrosis, and angiogenesis. In preclinical arthritis, mast cells seem to play a crucial role in inflammation and RA development [359]. In addition to the mast cells and FLS, dendritic cells contributed to the onset of RA pathogenesis. These cells play an important role in both innate and adaptive immune responses when exposed to exogenous antigens, via acting as antigen-presenting cells (APC) [360, 361]. Beside dendritic cells, the macrophage as well as B lymphocytes also work as antigen-presenting cells. They can process and present the arthritic antigens to T lymphocytes [362]. Two major serological autoantibodies which are defined as diagnostic markers for RA disease are rheumatoid factor (RF-IgM) and anticitrullinated protein antibodies (ACPA) [3]. It was found that the existence of the circulating autoantibodies such as ACPAs and RF, as well as proinflammatory proteins, including cytokines and chemokines, could be detected during the preclinical stages (~10 years before) [358]. The APCs expressing the MHC II of HLA-DR4 could bind and present the citrullinated peptides or autoantigens to be recognized by the adaptive immune cells, T and B lymphocytes in the RA joints and lymphatic tissues. CD4^+^ T-helper cells, mainly the Th1 subtype are activated and release IL-2 and IFN-*γ* which cross into the synovial membrane inducing joint inflammation. Likewise, B cells are involved in the pathogenicity of RA via antigen presentation to T cells and production of autoantibodies such RF and ACCP [361]. B cells have been involved in the production of ACPAs many years before the onset of RA [363], whereas, Th1 cells have an important role in mediating rheumatoid arthritis. Recently, another subtype of T cells (Th17) are focused to play a major role in RA inflammation. As detailed above, the Th17 lymphocytes produce many proinflammatory cytokines such as IL-17A, IL-17F, IL-21, IL-22, and TNF-*α* [184, 364]. TGF-*β*, IL-1*β*, IL-6, IL-21, and IL-23 stimulate the differentiation of Th17 from native T cells and inhibit the differentiation of the regulatory T cells (Tregs), thus the hemostasis is shifting towards inflammation [365].

TNF-*α* is reflected by this imbalance between Th17 and Treg cells where it shares in blocking the Treg cell activity [366]. The Th17 cells are reduced in the established RA due to their plasticity to other T cells. This reduction resulted from the shifting to Th1 or Treg cells [333, 367]. As described before, in addition to antibody production, B cells have a significant role in the pathogenesis of RA through acting as an antigen-presenting cell and through cytokine production (e.g., IL-6, TNF-*α*, and LT-*β*) [368]. Interlukine-6 (IL-6) plays an important role in the inflammation of RA. In chronic RA inflammation, IL-6 contributed to the adaptive immunity as it stimulates the differentiation of B cells into plasma cells resulting in hypergammaglobulinemia of the autoantibody. In addition, IL-6 induces the differentiation of T cells into cytotoxic CD8^+^ T cells. On the other hand, IL-6 inhibits Treg cell differentiation which disrupted the immunological tolerance and development of autoimmunity. Furthermore, IL-6 is involved in different processes of acute inflammation by the induction of acute-phase protein such as C-reactive protein (CRP) [369]. Other immune cells such as monocytes and granulocytes also infiltrate the synovium and contribute to joint destruction. The production of ACCP will cause the continuation of joint inflammation and increase the chronicity and severity of RA [370].

#### 5.4.5. Classification Criteria of RA

RA diagnosis is performed according to clinical symptoms and diagnostic tests, including serology, radiology, and histopathology investigations [371]. The RA diagnosis could be complicated in the initial stages of disease due to the lack of the laboratory and radiology evidences [372]. The clinical symptoms of RA can be categorized as articular and extraarticular, and patients might present different symptoms such as fever, weakness, tiredness, muscle pain, and weight loss before the initial articular manifestations [373]. In the articular manifestation, the basic synovial inflammation could target any synovial joint in the body [371]. Clinically, patients are suffering pain, swelling, and impaired movement of the impacted joints associated with the physical appearance of heat, ruddiness, and effusion at the affected joints. These findings could be observed in less deeper joints such as hand joints, feet, and elbow, rather than deep joints (hip and shoulder) [373].

In the extraarticular manifestations of RA, the most common complaints include pleuropulmonary, cardiac, neurological, and dermatological conditions. These findings could be reported in advanced stages in association with rheumatoid nodules and high positive serology of RF and ACCP [374, 375]. The classification criteria for rheumatoid arthritis were established by the American College of Rheumatology (ACR) in 1987 [376]. These criteria were used to distinguish between RA and other rheumatic diseases (Table 5). These criteria also have been argued for their insensitivity in the early stage [377–379].

In 2010, the combination of the criteria of the American College of Rheumatology (ACR) and the criteria of the European League Against Rheumatism (EULAR) produced a new RA classification criteria, known as the 2010 ACR/European League Against Rheumatism (EULAR) criteria. Recently, ACPA has been more specific than RF in the diagnosis of RA, but they have comparable sensitivity. These new criteria include more specific evidences and focus on the early diagnosis of RA improved by the presence of ACPA [379]. These new criteria considered individuals susceptible for inflammation progression and erosive disease and indicated the possibility of early treatment that could help in the prevention of the disease development [380]. The new ACR/EULAR classification criteria (Table 9) can be used for the diagnosis of any patient, but it requires the presence of clinical synovial tissue inflammation of one joint at least at the time of examination which is not related or linked to another disease [371].

#### 5.4.6. Rheumatoid Arthritis in Saudi Arabia

According to the Arab Society of Rheumatic Diseases, the number of the RA cases in Saudi Arabia is about 250,000 cases for 2011, and there are around 1/3000 new cases annually (https://www.okaz.com.sa/article/385561). Different studies have been focused on the status of RA in the Kingdom of Saudi Arabia. Studies were performed in two regions; Al-Qassim and Hail regions. In patients with negative RA family history, these studies showed a prevalence of RA in females more than in males with a high incidence in the fourth and the third decade of life mainly over 35 years. A high number of the patients involved in these studies were reported to complain of morning stiffness and inflammation mostly at the knee joint. Other most common joints involved were proximal interphalangeal joints (PIJ), metacarpophalangeal joints (MCP), wrists, and elbows [381, 382]. It is necessary to know the burden of RA in the Kingdom of Saudi Arabia (KSA), because the disease has been known to put an economic load on the health sector and on the community as well. Unfortunately, the degree of RA in the whole Saudi population is not fully estimated [382]. However, the disease has been demonstrated to be the most frequent inflammatory arthritis among the hospital attendants in KSA, and seems to be less intense than those of developed countries [381].

## 6. Citrullinated Proteins Are a Target for Circulating Autoantibodies

As mentioned before, the secreted antibodies recognize the modified amino acid citrulline within the targeted protein, and these autoantibodies directed to the citrullinated peptides or proteins are known as anticitrullinated peptide antibodies (ACPA) [383]. The breakthrough of ACPA in 1960 was a very important discovery that helped in the diagnosis, organization, and treatment of the most common autoimmune disorders, especially rheumatoid arthritis (RA) [384, 385]. In the synovium of RA patients, multiple citrullinated proteins were detected to produce an antigenic peptide that can induce a humoral response and the subsequent production of autoantibodies. The most abundant modified proteins in RA synovial tissue are vimentin, fibrinogen, collagen II, and *α*-enolase [386]. Each of these proteins has been characterized to have many citrullinate sites that act as antigens [330]. It is important to note that filaggrin is not present in the synovial joint compared with the other proteins [387–391]. Indeed, autoantibodies against filaggrin cannot trigger the autoimmunity in synovium directly, but they can bind to synovial proteins such as *α* and *β* chains of fibrin and then they can produce cross-reactivity [349, 392]. Collagen II and fibrinogen are extracellular proteins, whereas vimentin and *α*-enolase are detected intracellularly, with the exception of vimentin which could be found extracellularly. It is found abundantly in monocytes, and it could be secreted by activated macrophages [393].

### 6.1. Anticitrullinated Peptide Antibodies (ACPA) Are a Marker for RA

The ACPAs could react to the citrullinated form of several proteins, including *α* and *β* chains of fibrin (fibrinogen), *α*-enolase, vimentin, collagen II, fibronectin, filaggrin, and nuclear proteins such as histones [386, 394, 395]. Different types of ACPA have been recognized and characterized for evaluating the presence of autoantibodies in RA patients' serum such as antikeratin antibodies (AKA), antiperinuclear factor antibodies (APF), anti-Sa, anti-CCP, and anti-MCV. Antikeratin antibodies (AKA) and antiperinuclear factor antibodies (APF) that were discovered in 1960 [385] are considered the oldest member of this ACPA family which are used for RA laboratory diagnosis. AKA and APF have a limited specificity because they can react with citrullinated filaggrin as antifilaggrin antibodies (AFA). Laboratory tests such as immunofluorescence assays have been used to detect the presence of AFA in RA sera. The results showed that about 50% of the cases presented positive signals for AFA [370]. Autoantibodies against placenta Sa-antigen (Anti-Sa) have been earlier used for the diagnosis of RA [396]. These antibodies, which later correspond to citrullinated vimentin (not mutated), were evaluated using serum from RA patients and were found to be detected in approximately 40% of the severe cases [397]. The results showed that about 50% of the cases presented positive signals for AFA [370]. Autoantibodies against placenta Sa-antigen (Anti-Sa) have been earlier used for the diagnosis of RA [396]. These antibodies, which later correspond to citrullinated vimentin (not mutated), were evaluated using serum from RA patients and were found to be detected in approximately 40% of the severe cases [398]. In 2010, the ACR/EULAR classification criteria involved the ACPAs as biomarkers, especially the anti-CCP beside the RF [379]. The combination of RF and anti-CCP fuel the diagnostic sensitivity and specificity and give an early prognosis for the RA development, which is improved by the positivity for anti-CCP, which could be detected in the patient's serum up to 10 years before the appearance of clinical symptoms [399]. RF is a diagnostic marker for RA autoimmune diseases. The RF-IgM is the most detectable isotype and its sensitivity can reach 80% [400]. Indeed, RF has low specificity due to its presence in healthy individuals and in other autoimmune disorders and infectious diseases [14].

### 6.2. Anticyclic Citrullinated Peptide Assay for the Detection of ACPAs

A new technical assay that uses commercially synthesized cyclic citrullinated peptides as antigens is now available for the testing and detection of ACPA and is known as the anti-CCP test [401]. There are many advantages of using cyclic citrullinated peptides as antigens for anti-CCP; the cyclic form of the synthesized peptides increases antigen stability and test accuracy [402]. In addition, the manufacturing of CCP is easy and inexpensive [370]. The first generation of anti-CCP testing (anti-CCP1) was available on the market in 2000. It used a synthesized cyclic form of citrullinated filaggrin [392]. The anti-CCP1 testing of RA patients' sera was evaluated in the laboratory using the ELISA immunoassay procedure, and it revealed a sensitivity that reaches 60-70% and a specificity of 97-98% [397, 402]. The most important features of the diagnostic markers should include the high sensitivity for detecting a large number of cases, high specificity for minimizing the false-positive results, and the early appearance to predict the early diagnosis [370]. According to these required features in the diagnostic assay, the second generation of the anti-CCP testing kit (anti-CCP2) was developed in 2002 to increase the sensitivity [403, 404]. Because the synthesized filaggrin used in anti-CCP1 is not a synovial protein, a lot of efforts have been exerted to produce inclusive peptides other than filaggrin that contain a wide range of epitopes for the detection of ACPAs [405]. Several specific synthetic peptides were obtained from a synthetic peptide library [14] which had been screened and pooled with the serum of RA patients to develop the second generation of CCP (anti-CCP2). The anti-CCP2 generation displays specificity and sensitivity of 98% and 80%, respectively, which is higher than anti-CCP1 and similar to that of RF [406]. Now, anti-CCP2 is recognized as the gold standard method using IgG for the detection of ACPAs [407, 408]. Recently, different manufacturers produced a new generation (third) of cyclic synthesized peptides designated as the anti-CCP3 test [404]. Several reports showed that anti-CCP3 has higher sensitivity than anti-CCP2, especially in a smaller patient population with early RA [409], whereas both anti-CCP2 and anti-CCP3 have a similar specificity [410]. Other reports found that the anti-CCP3 is more prevalent than anti-CCP2 in RA patients with negative RF [343]. Recently, a new CCP testing assay was manufactured and offered to the market as anti-CCP3.1; this generation has the ability to detect IgG and IgA isotypes of ACPAs. Anti-CCP3.1 is reported by companies to have a slightly increased sensitivity compared to previously released generations [410].

### 6.3. Antimutated Citrullinated Vimentin (Anti-MCV)

Vimentin is an intermediate filament present in the synovial tissues, and it can be modified by PAD enzymes and expressed in the RA synovium as Sa-antigen corresponding to citrullinated vimentin (CV). Sa-antigen could be triggered by autoimmune antibodies known as anti-Sa antibodies, which could be used as markers for RA diagnosis, but with low sensitivity (40% of RA patients) [411–414]. Another isoform of the vimentin protein was characterized and demonstrated by a mass-spectroscopy technique; this vimentin appeared to have a mutation and citrullination at the same time. The glycine residue mutated into arginine, while arginine is citrullinated to produce antigenic mutated citrullinated vimentin (MCV) peptide. Thus, MCV has the ability to trigger the production of autoantibodies against MCV protein. These ACPAs are named antimutated citrullinated vimentin (anti-MCV) antibodies and recognized as a diagnostic marker for early RA. Accordingly, vimentin was mutated and citrullinated to yield commercially the MCV peptide used for testing anti-MCV [415]. An expanded meta-analysis performed in 2010 showed that the anti-MCV assay for RA sera has an elevated sensitivity and low specificity when compared to an anti-CCP testing assay [416]. Other studies reported the presence of an anti-MCV IgG isotype in RA patients with a negative RF and anti-CCP [417]. Moreover, reports had confirmed that the anti-MCV are highly correlated with anti-CCP2 rather than RF [418].

### 6.4. Evaluation of the Significance of ACPAs in the Diagnosis of RA in Saudi Patients

In Saudi Arabia, a few studies were performed on RA patients concerning the anticitrullinated peptide antibodies (anti-CCP, anti-MCV) and investigated the presence of these autoantibodies in association with other factors and clinical evidence. One of these studies assessed the prevalence of anti-cyclic citrullinated peptide antibodies (anti-CCP) in Saudi RA patients of negative RF. Patients' sera with increased disease activity were screened for anti-CCP2, and the results revealed that anti-CCP antibodies were present in some patients but were absent in others with the same disease severity. This finding may raise a conclusion that the anti-CCP cannot be used alone to predict disease activity or to manage treatment of RA patients [419]. Another study was concerned with the association between the genetic risk factor and the development of anti-CCP antibodies in Saudi RA patients. HLA typing was performed to detect the presence of HLA-DRB1 shared epitopes (SE), which is known as a genetic risk factor contributing to RA. The results obtained indicated that there is a high association between the development of anti-CCP and the presence of HLA-DRB1 shared epitopes in Saudi patients with RA [420]. In addition, the prevalence of anti-MCV antibodies in combination with RF was evaluated in both national and international patients. The results showed that there is a considerable presence of anti-MCV antibodies in patients with positive RF. Thus, this indicated that anti-MCV antibodies correlated with RF are a valuable diagnostic marker for RA [421].

## 7. Conclusion

The citrullination process is induced by different factors and catalyzed by peptidylarginine deiminase enzymes, resulting in the chemical and physical changes of the targeted peptides or proteins. The products of this process are citrullinated proteins, which contain a nonessential amino acid, citrulline. The immune system can recognize the deiminated or citrullinated proteins as nonself-antigens and so mount an autoimmune response. This autoimmune response yielded anticitrullinated peptide autoantibodies (ACPAs) which lead to the destruction of these proteins and their surrounding tissues causing differential pathological autoimmune disorders. The pathological effects of this process include the degeneration of the central nervous system such as in Alzheimer's disease, inflammatory disease such as rheumatoid arthritis, and other autoimmune disorders. In addition to the clinical investigation of the patients, a serological examination for the presence of ACPAs is very helpful in the early diagnosis of autoimmune disorders, especially in RA patients.

## Figures and Tables

**Table 1:** Protein shape and arginine position effect on citrullination efficiency.

Arginine position	Kinetic of citrullination	Protein shape	Kinetics of citrullination
N-Arg-Glu-C	Hardly citrullinated	*α*-Helix	Difficult to be citrullinated
N-Arg-Asp-C	Highly and efficiently citrullination (100%)	*β*-Turn	The most liable form for citrullination
N-Arg-Arg-C	Arg near C-terminus excluding MBP case	Disordered	Easily and efficiently citrullinated (95%)
N-Pro-Arg-Pro-C	Never citrullinated	*β*-Sheet	No data available
Arg close to N-terminus	Difficult to be citrullinated		

**Table 2:** Percentage of similarity (homology) between PADs, isoelectric point (*pI*), and calculated molecular mass (kDa) of human PADs.

% homology	PAD1	PAD2	PAD3	PAD4	PAD6	Pl	kDa
PAD1	100	65	68	71	59	6.01	74.6
PAD2		100	67	65	59	5.4	75.3
PAD3			100	68	60	5.25	74.6
PAD4					61	6.25	74.0
PAD6					100	4.97	77.7

**Table 3:** Body distribution, target substrates, and normal physiology and pathology of PADs.

Isotype	Expression	Substrates	Biological process	Pathological process
PAD1	Epidermis and uterus	Keratin K1 and filaggrin	Cornification of epidermal tissues	Psoriasis
PAD2	Widely expressed: pituitary gland, brain, uterus, spleen, spinal cord, and skeletal muscle	MBP, GFAP, vimentin, and *β* and *γ*-actin histones (H3 and H4)	Plasticity of the CNS, transcription regulation, innate immunity, and female fertility	Multiple sclerosis, rheumatoid arthritis, Alzheimer's disease, and prion disease
PAD3	Epidermis and hair follicles	Filaggrin and trichohyalin	Regulation of epidermal functions	Unknown
PAD4	Neutrophils, monocytes, macrophages, mammary glands, epithelial cells, and tumors	Histones H2A, H3, and H4; ING4; p300/CBP; nucleophosmin; and nuclear Lamin C	Chromatin decondensation, transcription regulation, tumor formation, innate immune response, and NETosis process	Rheumatoid arthritis, multiple sclerosis, and cancers
PAD6	Eggs, ovary, early fetus, and testis tissues	Protamine	Ovocyte, sperm chromatin decondensation, female productivity, cytoskeleton formation, early fetal growth, and target for contraceptive drugs	Unknown

**Table 4:** Major characteristics of MS courses as classified by [131].

Clinical form	Disease course
Clinically isolated syndrome (CIS)	Identified by acute or subacute onset of monophasic episode suggestive of MS that does not complete the current MS criteria. The episode persists for more than 24 h and commonly impacts the optic nerve, brain stem, or spinal cord
	About 30% up to 70% of the CIS cases obtain MS
	About 10%-85% of patients with optic neuritis can develop MS
	About 50%-60% of patients with brainstem syndromes and optic neuritis can develop MS
	About 40%-60% of patients with spinal cord can develop MS
	The age of initial diagnosis is between 20 and 45 years
	Women to men ratio ranging from 2 : 1 to 5 : 1

Relapsing-remitting MS (RRMS)	Manifested by relapses persisting for days to weeks, followed by complete or partial remissions continuing for months or years
	Represents about 85% of cases
	The age of initial diagnosis between 20 and 30 years
	Women to men ratio between 2 : 1 and 3 : 1

Secondary progressive MS (SPMS)	Manifested by increasing of disability after the first relapsing period of the disease
	About 75% of RRMS cases developed into SPMS within the first 15 years of diagnosis

Primary progressive MS (PPMS)	Identified by constant functional deterioration from the beginning of the disease
	Represent about 15% of cases
	Appears after RRMS (10 years)
	Women to men ratio 1 : 1

**Table 5:** Classification criteria for rheumatoid arthritis established by the American College of Rheumatology (ACR) in 1987.

Criteria	Definition
Morning stiffness	Morning stiffness persisting at least 1 hour before maximal progression
Arthritis of 3 or more joint areas	Presence of swelling in three or more joints, including the right or left PIP, MCP, wrist, elbow, knee, ankle, and MTP joints
Arthritis of hand joints	Swelling of one or more of hand joints, including wrist, MCP, or PIP joint
Symmetric arthritis	Concomitant involvement of the same joint areas on both sides of the body
Rheumatoid nodules	Clinical observation of subcutaneous nodules over bony prominences, extensor surfaces, or in juxta-articular bones as seen by a physician
Serum rheumatoid factor	Positive or elevated level of serum rheumatoid factor measured by laboratory method
Radiographic changes	Radiographic changes involve erosions in joint on posteroanterior hand and wrist, also displaying thinning of juxta-articular region

**Table 6:** Saudi population (65 years and over) organized by age groups.

Age groups	Males	Females	Total
65-69	150,777	159,582	310,359
70-74	10,005	112,813	222,818
75-79	71,142	72,937	144,079
Above 80	85,468	91,557	177,025
Total	417,392	436,889	854,281

**Table 7:** Saudi population (65 years and over) by age groups and chronic diseases.

Age groups	Alzheimer disease	Diabetes mellitus	Hypertension	Heart disease	Kidney disease
65-69	410	108,765	105,450	23,070	6122
70-74	2802	90,796	82,160	22,779	3775
75-79	2541	64,079	65,473	15,678	3358
Above 80	7590	68,902	76,751	27,808	5076
Total	13,343	332,542	329,834	89,335	18,331

**Table 8:** The immunological parameters included in the Systemic Lupus International Collaborating Clinics (SLICC) criteria.

Serological test	Result description
Antinuclear antibody (ANA)	Positive (exceeds reference range)
Anti-double-stranded antibody (anti-ds)	Positive (exceeds reference range)
Anti-Smith antibody (anti-Sm)	Positive for anti-Sm
Antiphospholipid antibody	
(i) Lupus anticoagulant antibody (LA)	Positive for LA
(ii) Antibodies against cardiolipin (aCL)	Medium-high titer for aCL (IgG, IgA, and IgM)
(iii) Antibodies against *β*2 glycoprotein I (anti-*β*2GPI)	Positive for anti-*β*2GPI (IgG, IgA, and IgM)
(iv) Rapid plasma regain (RPR)	False positive (in the absence of hemolytic anemia)
Complements	Low level of C3, C4, or CHO50
Direct Coombs' test (DCT)	Positive (clumping of RBCs)

**Table 9:** 2010 ACR/EULAR classification criteria for rheumatoid arthritis. A score ≥ 6 is required for the diagnosis of a patient with confirmed RA.

Joint involvement	Score (0-5)	Serology	Score (0-3)	Period of symptoms	Score (0-1)	Acute-phase reactants	Score (0-1)
1 large joint	0	Negative RF and negative ACPA	0	<6 weeks	0	Normal level of CRP and ESR	0
2–10 large joints	1	Strong positive RF or week positive ACPA	2	≥6 weeks	1	Abnormal level of CRP and ESR	1
1–3 small joints (with or without accounting large joints)	2	Strong positive RF or strong positive ACPA	3				
4–10 small joints (with or without accounting large joints)	3						
>10 joints (with minimum 1 small joint)	5						

**Table 10:** Distribution and incidence of RA.

Studied group	Incidence ratio
Females vs males	2 : 1 to 3 : 1
Caucasian from North America	100 case per 100,000
Rural and urban Africans	20-90 case per 100,000
Native Americans	500 case per 100,000
Asians	20-45 case per 100,000
Caucasian from Europe	5-89 case per 100,000
Latin America	10-50 case per 100,000
Middle East countries	10-50 case per 100,000

## References

[1] Rogers G. E., Simmonds D. H. (1958). Content of citrulline and other amino-acids in a protein of hair follicles. *Nature*.

[2] Vossenaar E. R., Zendman A. J. W., van Venrooij W. J., Pruijn G. J. M. (2003). PAD, a growing family of citrullinating enzymes: genes, features and involvement in disease. *BioEssays*.

[3] György B., Tóth E., Tarcsa E., Falus A., Buzás E. I. (2006). Citrullination: a posttranslational modification in health and disease. *The International Journal of Biochemistry & Cell Biology*.

[4] van Venrooij W. J., Pruijn G. J. M. (2000). Citrullination: a small change for a protein with great consequences for rheumatoid arthritis. *Arthritis Research*.

[5] Knuckley B., Causey C. P., Jones J. E. (2010). Substrate specificity and kinetic studies of PADs 1, 3, and 4 identify potent and selective inhibitors of protein arginine deiminase 3. *Biochemistry*.

[6] Tarcsa E., Marekov L. N., Mei G., Melino G., Lee S. C., Steinert P. M. (1996). Protein unfolding by peptidylarginine deiminase. *Journal of Biological Chemistry*.

[7] Slade D. J., Subramanian V., Fuhrmann J., Thompson P. R. (2014). Chemical and biological methods to detect post‐translational modifications of arginine. *Biopolymers*.

[8] Fearon W. R. (1939). The carbamido diacetyl reaction: a test for citrulline. *Biochemical Journal*.

[9] Bennike T., Lauridsen K. B., Olesen M. K., Andersen V., Birkelund S., Stensballe A. (2013). Optimizing the identification of citrullinated peptides by mass spectrometry: utilizing the inability of trypsin to cleave after citrullinated amino acids. *Journal of Proteomics & Bioinformatics*.

[10] Curis E., Nicolis I., Moinard C. (2005). Almost all about citrulline in mammals. *Amino Acids*.

[11] RGS C., van Rosmalen J. W. G., Jenniskens G. J., Pruijn G. J., JMH R. (2013). Citrullination: a target for disease intervention in multiple sclerosis and other inflammatory diseases?. *Journal of Clinical & Cellular Immunology*.

[12] Orgovan G., Noszal B. (2011). The complete microspeciation of arginine and citrulline. *Journal of Pharmaceutical and Biomedical Analysis*.

[13] Valesini G., Gerardi M. C., Iannuccelli C., Pacucci V. A., Pendolino M., Shoenfeld Y. (2015). Citrullination and autoimmunity. *Autoimmunity Reviews*.

[14] van Venrooij W. J., van Beers J. J. B. C., Pruijn G. J. M. (2011). Anti-CCP antibodies: the past, the present and the future. *Nature Reviews Rheumatology*.

[15] Rogers G. E., Harding H. W. J., Llewellyn-Smith I. J. (1977). The origin of citrulline-containing proteins in the hair follicle and the chemical nature of trichohyalin, an intracellular precursor. *Biochimica et Biophysica Acta (BBA) - Protein Structure*.

[16] Witalison E., Thompson P., Hofseth L. (2015). Protein arginine deiminases and associated citrullination: physiological functions and diseases associated with dysregulation. *Current Drug Targets*.

[17] Jones J. E., Causey C. P., Knuckley B., Slack-Noyes J. L., Thompson P. R. (2009). Protein arginine deiminase 4 (PAD4): current understanding and future therapeutic potential. *Current Opinion in Drug Discovery & Development*.

[18] Arita K., Hashimoto H., Shimizu T., Nakashima K., Yamada M., Sato M. (2004). Structural basis for Ca^2+^-induced activation of human PAD4. *Nature Structural & Molecular Biology*.

[19] Cherrington B. D., Morency E., Struble A. M., Coonrod S. A., Wakshlag J. J. (2010). Potential role for peptidylarginine deiminase 2 (PAD2) in citrullination of canine mammary epithelial cell histones. *PLoS One*.

[20] Jang B., Shin H. Y., Choi J. K. (2011). Subcellular localization of peptidylarginine deiminase 2 and citrullinated proteins in brains of scrapie-infected mice: nuclear localization of PAD2 and membrane fraction-enriched citrullinated proteins. *Journal of Neuropathology & Experimental Neurology*.

[21] Asaga H., Nakashima K., Senshu T., Ishigami A., Yamada M. (2001). Immunocytochemical localization of peptidylarginine deiminase in human eosinophils and neutrophils. *Journal of Leukocyte Biology*.

[22] van Winkelhoff A. J., Loos B. G., van der Reijden W. A., van der Velden U. (2002). *Porphyromonas gingivalis*, *Bacteroides forsythus* and other putative periodontal pathogens in subjects with and without periodontal destruction. *Journal of Clinical Periodontology*.

[23] McGraw W. T., Potempa J., Farley D., Travis J. (1999). Purification, characterization, and sequence analysis of a potential virulence factor from *Porphyromonas gingivalis*, peptidylarginine deiminase. *Infection and Immunity*.

[24] Abdullah S. N., Farmer E. A., Spargo L., Logan R., Gully N. (2013). *Porphyromonas gingivalis* peptidylarginine deiminase substrate specificity. *Anaerobe*.

[25] Saris N. E. L., Carafoli E. (2005). A historical review of cellular calcium handling, with emphasis on mitochondria. *Biochemistry*.

[26] Méchin M. C., Enji M., Nachat R. (2005). The peptidylarginine deiminases expressed in human epidermis differ in their substrate specificities and subcellular locations. *Cellular and Molecular Life Sciences*.

[27] Alghamdi M., al Ghamdi K. A., Khan R. H., Uversky V. N., Redwan E. M. (2019). An interplay of structure and intrinsic disorder in the functionality of peptidylarginine deiminases, a family of key autoimmunity-related enzymes. *Cellular and Molecular Life Sciences*.

[28] Machold K. P., Stamm T. A., Nell V. P. (2007). Very recent onset rheumatoid arthritis: clinical and serological patient characteristics associated with radiographic progression over the first years of disease. *Rheumatology*.

[29] Ishigami A., Maruyama N. (2010). Importance of research on peptidylarginine deiminase and citrullinated proteins in age‐related disease. *Geriatrics & Gerontology International*.

[30] Méchin M.-C., Sebbag M., Arnaud J. (2007). Update on peptidylarginine deiminases and deimination in skin physiology and severe human diseases. *International Journal of Cosmetic Science*.

[31] Hensen S. M. M., Pruijn G. J. M. (2014). Methods for the detection of peptidylarginine deiminase (PAD) activity and protein citrullination. *Molecular & Cellular Proteomics*.

[32] Tsuji Y., Akiyama M., Arita K., Senshu T., Shimizu H. (2003). Changing pattern of deiminated proteins in developing human epidermis. *Journal of Investigative Dermatology*.

[33] Tarcsa E., Marekov L. N., Andreoli J. (1997). The fate of trichohyalin. Sequential post-translational modifications by peptidyl-arginine deiminase and transglutaminases. *Journal of Biological Chemistry*.

[34] Moscarello M. A., Wood D. D., Ackerley C., Boulias C. (1994). Myelin in multiple sclerosis is developmentally immature. *The Journal of Clinical Investigation*.

[35] Baeten D., Peene I., Union A. (2001). Specific presence of intracellular citrullinated proteins in rheumatoid arthritis synovium: relevance to antifilaggrin autoantibodies. *Arthritis & Rheumatism*.

[36] Chapuy-Regaud S., Sebbag M., Baeten D. (2005). Fibrin deimination in synovial tissue is not specific for rheumatoid arthritis but commonly occurs during synovitides. *The Journal of Immunology*.

[37] Vossenaar E. R., Smeets T. J. M., Kraan M. C., Raats J. M., van Venrooij W. J., Tak P. P. (2004). The presence of citrullinated proteins is not specific for rheumatoid synovial tissue. *Arthritis & Rheumatism*.

[38] Ying S., Simon M., Serre G., Takahar H. (2012). Peptidylarginine deiminases and protein deimination in skin physiopathology. *Psoriasis - A Systemic Disease*.

[39] Guerrin M., Ishigami A., Méchin M.-C. (2003). cDNA cloning, gene organization and expression analysis of human peptidylarginine deiminase type I. *Biochemical Journal*.

[40] Ying S., Dong S., Kawada A. (2009). Transcriptional regulation of peptidylarginine deiminase expression in human keratinocytes. *Journal of Dermatological Science*.

[41] Chavanas S., Méchin M. C., Takahara H. (2004). Comparative analysis of the mouse and human peptidylarginine deiminase gene clusters reveals highly conserved non-coding segments and a new human gene, *PADI6*. *Gene*.

[42] Zhang J., Dai J., Zhao E. (2004). cDNA cloning, gene organization and expression analysis of human peptidylarginine deiminase type VI. *Acta Biochimica Polonica*.

[43] Kan S., Asaga H., Senshu T. (1996). Detection of several families of deiminated proteins derived from filaggrin and keratins in guinea pig skin. *Zoological Science*.

[44] Senshu T., Akiyama K., Kan S., Asaga H., Ishigami A., Manabe M. (1995). Detection of deiminated proteins in rat skin: probing with a monospecific antibody after modification of citrulline residues. *Journal of Investigative Dermatology*.

[45] Mohanan S., Cherrington B. D., Horibata S., McElwee J. L., Thompson P. R., Coonrod S. A. (2012). Potential role of peptidylarginine deiminase enzymes and protein citrullination in cancer pathogenesis. *Biochemistry Research International*.

[46] Ishida-Yamamoto A., Senshu T., Eady R. A. J. (2002). Sequential reorganization of cornified cell keratin filaments involving filaggrin-mediated compaction and keratin 1 deimination. *Journal of Investigative Dermatology*.

[47] Vossenaar E. R., Nijenhuis S., Helsen M. M. A. (2003). Citrullination of synovial proteins in murine models of rheumatoid arthritis. *Arthritis & Rheumatism*.

[48] Chavanas S., Méchin M. C., Nachat R. (2006). Peptidylarginine deiminases and deimination in biology and pathology: relevance to skin homeostasis. *Journal of Dermatological Science*.

[49] Ishida-Yamamoto A., Takahashi H., Iizuka H., Senshu T., Akiyama K., Nomura K. (2000). Decreased deiminated keratin K1 in psoriatic hyperproliferative epidermis. *Journal of Investigative Dermatology*.

[50] Wang S., Wang Y. (2013). Peptidylarginine deiminases in citrullination, gene regulation, health and pathogenesis. *Biochimica et Biophysica Acta (BBA) - Gene Regulatory Mechanisms*.

[51] Pritzker L. B., Joshi S., Gowan J. J., Harauz G., Moscarello M. A. (2000). Deimination of myelin basic protein. 1. Effect of deimination of arginyl residues of myelin basic protein on its structure and susceptibility to digestion by cathepsin D. *Biochemistry*.

[52] Pritzker L. B., Joshi S., Harauz G., Moscarello M. A. (2000). Deimination of myelin basic protein. 2. Effect of methylation of MBP on its deimination by peptidylarginine deiminase. *Biochemistry*.

[53] Cherrington B. D., Zhang X., McElwee J. L., Morency E., Anguish L. J., Coonrod S. A. (2012). Potential role for PAD2 in gene regulation in breast cancer cells. *PLoS One*.

[54] Ishigami A., Ohsawa T., Asaga H., Akiyama K., Kuramoto M., Maruyama N. (2002). Human peptidylarginine deiminase type II: molecular cloning, gene organization, and expression in human skin. *Archives of Biochemistry and Biophysics*.

[55] Vossenaar E. R., Radstake T. R., van der Heijden A. (2004). Expression and activity of citrullinating peptidylarginine deiminase enzymes in monocytes and macrophages. *Annals of the Rheumatic Diseases*.

[56] Beniac D. R., Wood D. D., Palaniyar N., Ottensmeyer F. P., Moscarello M. A., Harauz G. (2000). Cryoelectron microscopy of protein-lipid complexes of human myelin basic protein charge isomers differing in degree of citrullination. *Journal of Structural Biology*.

[57] Asaga H., Yamada M., Senshu T. (1998). Selective deimination of vimentin in calcium ionophore-induced apoptosis of mouse peritoneal macrophages. *Biochemical and Biophysical Research Communications*.

[58] Pruitt K. D., Brown G. R., Hiatt S. M. (2014). RefSeq: an update on mammalian reference sequences. *Nucleic Acids Research*.

[59] Hsu P. C., Liao Y. F., Lin C. L., Lin W. H., Liu G. Y., Hung H. C. (2014). Vimentin is involved in peptidylarginine deiminase 2-induced apoptosis of activated Jurkat cells. *Molecules and Cells*.

[60] Hojo-Nakashima I., Sato R., Nakashima K., Hagiwara T., Yamada M. (2009). Dynamic expression of peptidylarginine deiminase 2 in human monocytic leukaemia THP-1 cells during macrophage differentiation. *The Journal of Biochemistry*.

[61] Chumanevich A. A., Causey C. P., Knuckley B. A. (2011). Suppression of colitis in mice by Cl-amidine: a novel peptidylarginine deiminase inhibitor. *American Journal of Physiology-Gastrointestinal and Liver Physiology*.

[62] Chang X., Han J., Pang L., Zhao Y., Yang Y., Shen Z. (2009). Increased PADI4 expression in blood and tissues of patients with malignant tumors. *BMC Cancer*.

[63] Bhattacharya S. K., Crabb J. S., Bonilha V. L., Gu X., Takahara H., Crabb J. W. (2006). Proteomics implicates peptidyl arginine deiminase 2 and optic nerve citrullination in glaucoma pathogenesis. *Investigative Ophthalmology & Visual Science*.

[64] Nachat R., Méchin M. C., Takahara H. (2005). Peptidylarginine deiminase isoforms 1–3 are expressed in the epidermis and involved in the deimination of K1 and filaggrin. *Journal of Investigative Dermatology*.

[65] Rus’d A. A., Ikejiri Y., Ono H. (1999). Molecular cloning of cDNAs of mouse peptidylarginine deiminase type I, type III and type IV, and the expression pattern of type I in mouse. *European Journal of Biochemistry*.

[66] Kanno T., Shiraiwa M., Takahara H. (2000). Human peptidylarginine deiminase type III: molecular cloning and nucleotide sequence of the cDNA, properties of the recombinant enzyme, and immunohistochemical localization in human skin. *Journal of Investigative Dermatology*.

[67] Lee S. C., Kim I. G., Marekov L. N., O’keefe E. J., Parry D. A., Steinert P. M. (1993). The structure of human trichohyalin. Potential multiple roles as a functional EF-hand-like calcium-binding protein, a cornified cell envelope precursor, and an intermediate filament-associated (cross-linking) protein. *Journal of Biological Chemistry*.

[68] Kin Pong U., Subramanian V., Nicholas A. P., Thompson P. R., Ferretti P. (2014). Modulation of calcium-induced cell death in human neural stem cells by the novel peptidylarginine deiminase-AIF pathway. *Biochimica et Biophysica Acta (BBA) - Molecular Cell Research*.

[69] Rogers G. E., Powell B. C. (1993). Organization and expression of hair follicle genes. *The Journal of Investigative Dermatology*.

[70] Chang X., Fang K. (2010). PADI4 and tumourigenesis. *Cancer Cell International*.

[71] Chang X., Han J. (2006). Expression of peptidylarginine deiminase type 4 (PAD4) in various tumors. *Molecular Carcinogenesis*.

[72] Sambandam T., Belousova M., Accaviti-Loper M. A. (2004). Increased peptidylarginine deiminase type II in hypoxic astrocytes. *Biochemical and Biophysical Research Communications*.

[73] Nakashima K., Hagiwara T., Yamada M. (2002). Nuclear localization of peptidylarginine deiminase V and histone deimination in granulocytes. *Journal of Biological Chemistry*.

[74] Liu Y. L., Chiang Y. H., Liu G. Y., Hung H. C. (2011). Functional role of dimerization of human peptidylarginine deiminase 4 (PAD4). *PLoS One*.

[75] Shirai H., Blundell T. L., Mizuguchi K. (2001). A novel superfamily of enzymes that catalyze the modification of guanidino groups. *Trends in Biochemical Sciences*.

[76] Kearney P. L., Bhatia M., Jones N. G. (2005). Kinetic characterization of protein arginine deiminase 4: a transcriptional corepressor implicated in the onset and progression of rheumatoid arthritis. *Biochemistry*.

[77] Wang Y., Wysocka J., Sayegh J. (2004). Human PAD4 regulates histone arginine methylation levels via demethylimination. *Science*.

[78] Lee Y. H., Coonrod S. A., Kraus W. L., Jelinek M. A., Stallcup M. R. (2005). Regulation of coactivator complex assembly and function by protein arginine methylation and demethylimination. *Proceedings of the National Academy of Sciences of the United States of America*.

[79] Cuthbert G. L., Daujat S., Snowden A. W. (2004). Histone deimination antagonizes arginine methylation. *Cell*.

[80] Wang Y., Li M., Stadler S. (2009). Histone hypercitrullination mediates chromatin decondensation and neutrophil extracellular trap formation. *Journal of Cell Biology*.

[81] Luo Y., Arita K., Bhatia M. (2006). Inhibitors and inactivators of protein arginine deiminase 4: functional and structural characterization. *Biochemistry*.

[82] Baka Z., György B., Géher P., Buzás E. I., Falus A., Nagy G. (2012). Citrullination under physiological and pathological conditions. *Joint Bone Spine*.

[83] Suzuki A., Yamada R., Chang X. (2003). Functional haplotypes of *PADI4*, encoding citrullinating enzyme peptidylarginine deiminase 4, are associated with rheumatoid arthritis. *Nature Genetics*.

[84] Yurttas P., Vitale A. M., Fitzhenry R. J. (2008). Role for PADI6 and the cytoplasmic lattices in ribosomal storage in oocytes and translational control in the early mouse embryo. *Development*.

[85] Wright P. W., Bolling L. C., Calvert M. E. (2003). ePAD, an oocyte and early embryo-abundant peptidylarginine deiminase-like protein that localizes to egg cytoplasmic sheets. *Developmental Biology*.

[86] Gossen J., Van Den Boogaart P. Peptidylarginine deiminase 6.

[87] Slack J. L., Jones L. E., Bhatia M. M., Thompson P. R. (2011). Autodeimination of protein arginine deiminase 4 alters protein-protein interactions but not activity. *Biochemistry*.

[88] Mastronardi F. G., Wood D. D., Mei J. (2006). Increased citrullination of histone H3 in multiple sclerosis brain and animal models of demyelination: a role for tumor necrosis factor-induced peptidylarginine deiminase 4 translocation. *The Journal of Neuroscience*.

[89] Moelants E. A. V., Mortier A., Grauwen K., Ronsse I., van Damme J., Proost P. (2013). Citrullination of TNF-*α* by peptidylarginine deiminases reduces its capacity to stimulate the production of inflammatory chemokines. *Cytokine*.

[90] Ali S., Nazir A., Shabbir S., Ashraf S. (2017). Understanding the role of peptidylarginine deiminases (PADs) in diseases and their inhibitors as potential therapeutic agents. *European Journal of Pharmaceutical and Medical Research*.

[91] Kaore S. N., Amane H. S., Kaore N. M. (2013). Citrulline: pharmacological perspectives and its role as an emerging biomarker in future. *Fundamental & Clinical Pharmacology*.

[92] Makrygiannakis D., af Klint E., Lundberg I. E. (2006). Citrullination is an inflammation-dependent process. *Annals of the Rheumatic Diseases*.

[93] Browne P., Chandraratna D., Angood C. (2014). Atlas of multiple sclerosis 2013: a growing global problem with widespread inequity. *Neurology*.

[94] Moscarello M. A., Mastronardi F. G., Wood D. D. (2007). The role of citrullinated proteins suggests a novel mechanism in the pathogenesis of multiple sclerosis. *Neurochemical Research*.

[95] Wood D. D., Moscarello M. A., Bilbao J. M., O’Connors P. (1996). Acute multiple sclerosis (Marburg type) is associated with developmentally immature myelin basic protein. *Annals of Neurology*.

[96] Pace S., Sautebin L., Werz O. (2017). Sex-biased eicosanoid biology: impact for sex differences in inflammation and consequences for pharmacotherapy. *Biochemical Pharmacology*.

[97] Susuki K. (2010). Myelin: a specialized membrane for cell communication. *Nature Education*.

[98] Brady G. W., Murthy N. S., Fein D. B., Wood D. D., Moscarello M. A. (1981). The effect of basic myelin protein on multilayer membrane formation. *Biophysical Journal*.

[99] Taylor C. M., Marta C. B., Bansal R., Pfeiffer S. E. (2004). *Myelin Biology and Disorders*.

[100] Quarles R. H., Macklin W. B., Morell P. (2006). Myelin formation, structure and biochemistry. *Basic Neurochemistry: Molecular, Cellular and Medical Aspects*.

[101] Patzig J., Jahn O., Tenzer S. (2011). Quantitative and integrative proteome analysis of peripheral nerve myelin identifies novel myelin proteins and candidate neuropathy loci. *The Journal of Neuroscience*.

[102] Jahn O., Tenzer S., Werner H. B. (2009). Myelin proteomics: molecular anatomy of an insulating sheath. *Molecular Neurobiology*.

[103] Greenfield S., Brostoff S., Eylar E. H., Morell P. (1973). Protein composition of myelin of the peripheral nervous system. *Journal of Neurochemistry*.

[104] Benjamins J. A., Morell P. (1978). Proteins of myelin and their metabolism. *Neurochemical Research*.

[105] Baumann N., Pham-Dinh D. (2001). Biology of oligodendrocyte and myelin in the mammalian central nervous system. *Physiological Reviews*.

[106] Riccio P., Masotti L., Cavatorta P. (1986). Myelin basic protein ability to organize lipid bilayers: structural transition in bilayers of lisophosphatidylcholine micelles. *Biochemical and Biophysical Research Communications*.

[107] Readhead C., Popko B., Takahashi N. (1987). Expression of a myelin basic protein gene in transgenic shiverer mice: correction of the dysmyelinating phenotype. *Cell*.

[108] Harauz G., Ladizhansky V., Boggs J. M. (2009). Structural polymorphism and multifunctionality of myelin basic protein. *Biochemistry*.

[109] Knoll W., Peters J., Kursula P. (2014). Structural and dynamical properties of reconstituted myelin sheaths in the presence of myelin proteins MBP and P2 studied by neutron scattering. *Soft Matter*.

[110] Baron W., Hoekstra D. (2010). On the biogenesis of myelin membranes: sorting, trafficking and cell polarity. *FEBS Letters*.

[111] Aggarwal S., Yurlova L., Simons M. (2011). Central nervous system myelin: structure, synthesis and assembly. *Trends in Cell Biology*.

[112] Musse A. A., Li Z., Ackerley C. A. (2008). Peptidylarginine deiminase 2 (PAD2) overexpression in transgenic mice leads to myelin loss in the central nervous system. *Disease Models & Mechanisms*.

[113] Wood D. D., Ackerley C. A., Brand B. . . (2008). Myelin localization of peptidylarginine deiminases 2 and 4: comparison of PAD2 and PAD4 activities. *Laboratory Investigation*.

[114] Carnegie P. R. (1971). Amino acid sequence of the encephalitogenic basic protein from human myelin. *Biochemical Journal*.

[115] Mendz G. L., Moore W., Carnegie P. (1982). N.M.R. studies of myelin basic protein. VI. Proton spectra in aqueous solutions of proteins from mammalian and avian species. *Australian Journal of Chemistry*.

[116] Warren K. G., Catz I., Steinman L. (1995). Fine specificity of the antibody response to myelin basic protein in the central nervous system in multiple sclerosis: the minimal B-cell epitope and a model of its features. *Proceedings of the National Academy of Sciences of the United States of America*.

[117] Bradford C., Nicholas A. P., Woodroofe N., Cross A. K. (2014). Deimination in multiple sclerosis and experimental autoimmune encephalomyelitis. *Protein Deimination in Human Health and Disease*.

[118] Raijmakers R., Vogelzangs J., Raats J. (2006). Experimental autoimmune encephalomyelitis induction in peptidylarginine deiminase 2 knockout mice. *The Journal of Comparative Neurology*.

[119] Moscarello M. A., Pritzker L., Mastronardi F. G., Wood D. D. (2002). Peptidylarginine deiminase: a candidate factor in demyelinating disease. *Journal of Neurochemistry*.

[120] Smith K. J. (2007). Sodium channels and multiple sclerosis: roles in symptom production, damage and therapy. *Brain Pathology*.

[121] Shideman C. R., Hu S., Peterson P. K., Thayer S. A. (2006). CCL5 evokes calcium signals in microglia through a kinase‐, phosphoinositide‐, and nucleotide-dependent mechanism. *Journal of Neuroscience Research*.

[122] Cao, Goodin R., Wood D., Moscarello M. A., Whitaker J. N. (1999). Rapid release and unusual stability of immunodominant peptide 45–89 from citrullinated myelin basic protein. *Biochemistry*.

[123] Boggs J. M., Rangaraj G., Koshy K. M., Ackerley C., Wood D. D., Moscarello M. A. (1999). Highly deiminated isoform of myelin basic protein from multiple sclerosis brain causes fragmentation of lipid vesicles. *Journal of Neuroscience Research*.

[124] Whitaker J. N., Bashir R. M., Chou C. H., Kibler R. F. (1980). Antigenic features of myelin basic protein-like material in cerebrospinal fluid. *The Journal of Immunology*.

[125] Bahrun U., Wijaya C. (2018). Immunology of multiple sclerosis. *Indonesian Journal of Clinical Pathology and Medical Laboratory*.

[126] Lublin F. D., Reingold S. C., National Multiple Sclerosis Society (USA) Advisory Committee on Clinical Trials of New Agents in Multiple Sclerosis (1996). Defining the clinical course of multiple sclerosis: results of an international survey. *Neurology*.

[127] Miller D., Barkhof F., Montalban X., Thompson A., Filippi M. (2005). Clinically isolated syndromes suggestive of multiple sclerosis, part I: natural history, pathogenesis, diagnosis, and prognosis. *The Lancet Neurology*.

[128] Miller D., Barkhof F., Montalban X., Thompson A., Filippi M. (2005). Clinically isolated syndromes suggestive of multiple sclerosis, part 2: non-conventional MRI, recovery processes, and management. *The Lancet Neurology*.

[129] Confavreux C., Aimard G., Devic M. (1980). Course and prognosis of multiple sclerosis assessed by the computerized data processing of 349 patients. *Brain*.

[130] Scalfari A., Neuhaus A., Daumer M., Ebers G. C., Muraro P. A. (2011). Age and disability accumulation in multiple sclerosis. *Neurology*.

[131] Lublin F. D., Reingold S. C., Cohen J. A. (2014). Defining the clinical course of multiple sclerosis: the 2013 revisions. *Neurology*.

[132] Scalfari A., Neuhaus A., Daumer M., Muraro P. A., Ebers G. C. (2013). Onset of secondary progressive phase and long-term evolution of multiple sclerosis. *Journal of Neurology, Neurosurgery & Psychiatry*.

[133] Fischer M. T., Sharma R., Lim J. L. (2012). NADPH oxidase expression in active multiple sclerosis lesions in relation to oxidative tissue damage and mitochondrial injury. *Brain*.

[134] Alliot F., Godin I., Pessac B. (1999). Microglia derive from progenitors, originating from the yolk sac, and which proliferate in the brain. *Developmental Brain Research*.

[135] Ginhoux F., Greter M., Leboeuf M. (2010). Fate mapping analysis reveals that adult microglia derive from primitive macrophages. *Science*.

[136] Nimmerjahn A., Kirchhoff F., Helmchen F. (2005). Resting microglial cells are highly dynamic surveillants of brain parenchyma in vivo. *Science*.

[137] van Horssen J., Singh S., van der Pol S. (2012). Clusters of activated microglia in normal-appearing white matter show signs of innate immune activation. *Journal of Neuroinflammation*.

[138] Lassmann H., Bruck W., Lucchinetti C. F. (2007). The immunopathology of multiple sclerosis: an overview. *Brain Pathology*.

[139] Marik C., Felts P. A., Bauer J., Lassmann H., Smith K. J. (2007). Lesion genesis in a subset of patients with multiple sclerosis: a role for innate immunity?. *Brain*.

[140] Barnett M. H., Prineas J. W. (2004). Relapsing and remitting multiple sclerosis: pathology of the newly forming lesion. *Annals of Neurology*.

[141] Bø L., Vedeler C. A., Nyland H., Trapp B. D., Mørk S. J. (2003). Intracortical multiple sclerosis lesions are not associated with increased lymphocyte infiltration. *Multiple Sclerosis Journal*.

[142] De Groot C. J. A., Bergers E., Kamphorst W. (2001). Post-mortem MRI-guided sampling of multiple sclerosis brain lesions: increased yield of active demyelinating and (p)reactive lesions. *Brain*.

[143] Kutzelnigg A., Lassmann H. (2014). Pathology of multiple sclerosis and related inflammatory demyelinating diseases. *Handbook of Clinical Neurology*.

[144] Stys P. K., Zamponi G. W., van Minnen J., Geurts J. J. G. (2012). Will the real multiple sclerosis please stand up?. *Nature Reviews Neuroscience*.

[145] van Noort J. M., van den Elsen P. J., van Horssen J., Geurts J. J. G., van der Valk P., Amor S. (2011). Preactive multiple sclerosis lesions offer novel clues for neuroprotective therapeutic strategies. *CNS & Neurological Disorders - Drug Targets*.

[146] Mallucci G., Peruzzotti-Jametti L., Bernstock J. D., Pluchino S. (2015). The role of immune cells, glia and neurons in white and gray matter pathology in multiple sclerosis. *Progress in Neurobiology*.

[147] Krogsgaard M., Wucherpfennig K. W., Canella B. (2000). Visualization of myelin basic protein (MBP) T cell epitopes in multiple sclerosis lesions using a monoclonal antibody specific for the human histocompatibility leukocyte antigen (HLA)-DR2-MBP 85–99 complex. *Journal of Experimental Medicine*.

[148] Li H., Cuzner M. L., Newcombe J. (1996). Microglia‐derived macrophages in early multiple sclerosis plaques. *Neuropathology and Applied Neurobiology*.

[149] Yang L., Tan D., Piao H. (2016). Myelin basic protein citrullination in multiple sclerosis: a potential therapeutic target for the pathology. *Neurochemical Research*.

[150] Napoli I., Neumann H. (2009). Microglial clearance function in health and disease. *Neuroscience*.

[151] Napoli I., Neumann H. (2010). Protective effects of microglia in multiple sclerosis. *Experimental Neurology*.

[152] Trebst C., Lykke Sørensen T., Kivisäkk P. (2001). CCR1+/CCR5+ mononuclear phagocytes accumulate in the central nervous system of patients with multiple sclerosis. *The American Journal of Pathology*.

[153] Dargahi N., Katsara M., Tselios T. (2017). Multiple sclerosis: immunopathology and treatment update. *Brain Sciences*.

[154] Peferoen L. A. N., Vogel D. Y. S., Ummenthum K. (2015). Activation status of human microglia is dependent on lesion formation stage and remyelination in multiple sclerosis. *Journal of Neuropathology & Experimental Neurology*.

[155] Strachan-Whaley M., Rivest S., Yong V. W. (2014). Interactions between microglia and T cells in multiple sclerosis pathobiology. *Journal of Interferon & Cytokine Research*.

[156] Weiner H. L. (2008). A shift from adaptive to innate immunity: a potential mechanism of disease progression in multiple sclerosis. *Journal of Neurology*.

[157] Monney L., Sabatos C. A., Gaglia J. L. (2002). Th1-specific cell surface protein Tim-3 regulates macrophage activation and severity of an autoimmune disease. *Nature*.

[158] Musse A. A., Harauz G. (2007). Molecular “negativity” may underlie multiple sclerosis: role of the myelin basic protein family in the pathogenesis of MS. *International Review of Neurobiology*.

[159] Lang H. L. E., Jacobsen H., Ikemizu S. (2002). A functional and structural basis for TCR cross-reactivity in multiple sclerosis. *Nature Immunology*.

[160] Brimnes M. K., Hansen B. E., Nielsen L. K., Dziegiel M. H., Nielsen C. H. (2014). Uptake and presentation of myelin basic protein by normal human B cells. *PLoS One*.

[161] Wucherpfennig K. W., Catz I., Hausmann S., Strominger J. L., Steinman L., Warren K. G. (1997). Recognition of the immunodominant myelin basic protein peptide by autoantibodies and HLA-DR2-restricted T cell clones from multiple sclerosis patients. Identity of key contact residues in the B-cell and T-cell epitopes. *The Journal of Clinical Investigation*.

[162] Wucherpfennig K. W., Sette A., Southwood S. (1994). Structural requirements for binding of an immunodominant myelin basic protein peptide to DR2 isotypes and for its recognition by human T cell clones. *Journal of Experimental Medicine*.

[163] Huseby E. S., Liggitt D., Brabb T., Schnabel B., Öhlén C., Goverman J. (2001). A pathogenic role for myelin-specific CD8^+^ T cells in a model for multiple sclerosis. *Journal of Experimental Medicine*.

[164] Sun D., Whitaker J. N., Huang Z. (2001). Myelin antigen-specific CD8^+^ T cells are encephalitogenic and produce severe disease in C57BL/6 mice. *The Journal of Immunology*.

[165] Luchtman D. W., Ellwardt E., Larochelle C., Zipp F. (2014). IL-17 and related cytokines involved in the pathology and immunotherapy of multiple sclerosis: current and future developments. *Cytokine & Growth Factor Reviews*.

[166] Rostami A., Ciric B. (2013). Role of Th17 cells in the pathogenesis of CNS inflammatory demyelination. *Journal of the Neurological Sciences*.

[167] Koning N., Ilarregui J. M., García-Vallejo J. J., van Kooyk Y. (2013). Antigen-presenting cells in the central nervous system. *Multiple Sclerosis Immunology*.

[168] Stockinger B., Veldhoen M. (2007). Differentiation and function of Th17 T cells. *Current Opinion in Immunology*.

[169] Galea I., Palin K., Newman T. A., van Rooijen N., Perry V. H., Boche D. (2005). Mannose receptor expression specifically reveals perivascular macrophages in normal, injured, and diseased mouse brain. *Glia*.

[170] Idoyaga J., Lubkin A., Fiorese C. (2011). Comparable T helper 1 (Th1) and CD8 T-cell immunity by targeting HIV gag p24 to CD8 dendritic cells within antibodies to Langerin, DEC205, and Clec9A. *Proceedings of the National Academy of Sciences of the United States of America*.

[171] Frischer J. M., Bramow S., Dal-Bianco A. (2009). The relation between inflammation and neurodegeneration in multiple sclerosis brains. *Brain*.

[172] Skulina C., Schmidt S., Dornmair K. (2004). Multiple sclerosis: brain-infiltrating CD8^+^ T cells persist as clonal expansions in the cerebrospinal fluid and blood. *Proceedings of the National Academy of Sciences of the United States of America*.

[173] Obermeier B., Lovato L., Mentele R. (2011). Related B cell clones that populate the CSF and CNS of patients with multiple sclerosis produce CSF immunoglobulin. *Journal of Neuroimmunology*.

[174] Fraussen J., de Bock L., Somers V. (2016). B cells and antibodies in progressive multiple sclerosis: contribution to neurodegeneration and progression. *Autoimmunity Reviews*.

[175] Comabella M., Cantó E., Nurtdinov R. (2016). MRI phenotypes with high neurodegeneration are associated with peripheral blood B-cell changes. *Human Molecular Genetics*.

[176] Martino G., Olsson T., Fredrikson S. (1991). Cells producing antibodies specific for myelin basic protein region 70–89 are predominant in cerebrospinal fluid from patients with multiple sclerosis. *European Journal of Immunology*.

[177] Soderstrom M., Link H., Xu Z., Fredriksson S. (1993). Optic neuritis and multiple sclerosis: anti-MBP and anti-MBP peptide antibody-secreting cells are accumulated in CSF. *Neurology*.

[178] Bodil Roth E., Theander E., Londos E. (2008). Pathogenesis of autoimmune diseases: antibodies against transglutaminase, peptidylarginine deiminase and protein‐bound citrulline in primary Sjögren’s syndrome, multiple sclerosis and Alzheimer’s disease. *Scandinavian Journal of Immunology*.

[179] de Seze J., Dubucquoi S., Lefranc D. (2001). IgG reactivity against citrullinated myelin basic protein in multiple sclerosis. *Journal of Neuroimmunology*.

[180] Reindl M., Linington C., Brehm U. (1999). Antibodies against the myelin oligodendrocyte glycoprotein and the myelin basic protein in multiple sclerosis and other neurological diseases: a comparative study. *Brain*.

[181] Kleinewietfeld M., Hafler D. A. (2013). The plasticity of human Treg and Th17 cells and its role in autoimmunity. *Seminars in Immunology*.

[182] Korn T., Bettelli E., Oukka M., Kuchroo V. K. (2009). IL-17 and Th17 cells. *Annual Review of Immunology*.

[183] Tahmasebinia F., Pourgholaminejad A. (2017). The role of Th17 cells in auto-inflammatory neurological disorders. *Progress in Neuro-Psychopharmacology and Biological Psychiatry*.

[184] Miossec P., Korn T., Kuchroo V. K. (2009). Interleukin-17 and type 17 helper T cells. *The New England Journal of Medicine*.

[185] Korn T., Bettelli E., Gao W. (2007). IL-21 initiates an alternative pathway to induce proinflammatory T_H_17 cells. *Nature*.

[186] Heydarpour P., Khoshkish S., Abtahi S., Moradi-Lakeh M., Sahraian M. A. (2015). Multiple sclerosis epidemiology in Middle East and North Africa: a systematic review and meta-analysis. *Neuroepidemiology*.

[187] Babaloo Z., Aliparasti M. R., Babaiea F., Almasi S., Baradaran B., Farhoudi M. (2015). The role of Th17 cells in patients with relapsing-remitting multiple sclerosis: interleukin-17A and interleukin-17F serum levels. *Immunology Letters*.

[188] Al Wutayd O., Mohamed A. G., Saeedi J., al Otaibi H., al Jumah M. (2018). Environmental exposures and the risk of multiple sclerosis in Saudi Arabia. *BMC Neurology*.

[189] Daif A. K., al-Rajeh S., Awada A. (1998). Pattern of presentation of multiple sclerosis in Saudi Arabia: analysis based on clinical and paraclinical features. *European Neurology*.

[190] Bohlega S., Inshasi J., Tahan A. R., Madani A. B., Qahtani H., Rieckmann P. (2013). Multiple sclerosis in the Arabian Gulf countries: a consensus statement. *Journal of Neurology*.

[191] Al Deeb S. (2009). Epidemiology of MS in Saudi Arabia. *Multiple Sclerosis*.

[192] Sundstrom P., Juto P., Wadell G. (2004). An altered immune response to Epstein-Barr virus in multiple sclerosis: a prospective study. *Neurology*.

[193] Kleinewietfeld M., Manzel A., Titze J. (2013). Sodium chloride drives autoimmune disease by the induction of pathogenic T_H_17 cells. *Nature*.

[194] Alhazzani A. A., Alqahtani M. S., Ogran H. (2018). Depression severity and its predictors among multiple sclerosis patients in Saudi Arabia: a cross-sectional study. *Neuroimmunology and Neuroinflammation*.

[195] Van de Velde S., Bracke P., Levecque K. (2010). Gender differences in depression in 23 European countries. Cross-national variation in the gender gap in depression. *Social Science & Medicine*.

[196] Andrade L., Caraveo-anduaga J. J., Berglund P. (2003). The epidemiology of major depressive episodes: results from the International Consortium of Psychiatric Epidemiology (ICPE) surveys. *International Journal of Methods in Psychiatric Research*.

[197] Salloway S., Correia S. (2009). Alzheimer disease: time to improve its diagnosis and treatment. *Cleveland Clinic Journal of Medicine*.

[198] Imbimbo B. P., Lombard J., Pomara N. (2005). Pathophysiology of Alzheimer’s disease. *Neuroimaging Clinics of North America*.

[199] Ishigami A., Ohsawa T., Hiratsuka M. (2005). Abnormal accumulation of citrullinated proteins catalyzed by peptidylarginine deiminase in hippocampal extracts from patients with Alzheimer’s disease. *Journal of Neuroscience Research*.

[200] Armstrong R. A. (2006). Plaques and tangles and the pathogenesis of Alzheimer’s disease. *Folia Neuropathologica*.

[201] Khachaturian Z. S. (1985). Diagnosis of Alzheimer’s disease. *Archives of Neurology*.

[202] Mirra S. S., Heyman A., McKeel D. (1991). The Consortium to Establish a Registry for Alzheimer’s Disease (CERAD): part II. Standardization of the neuropathologic assessment of Alzheimer’s disease. *Neurology*.

[203] Armstrong R. A. (2013). What causes Alzheimer’s disease?. *Folia Neuropathologica*.

[204] Chen Y., Swanson R. A. (2003). Astrocytes and brain injury. *Journal of Cerebral Blood Flow & Metabolism*.

[205] Céspedes Á. E., Arango C. A., Cardona G. P. (2013). Análisis comparativo de marcadores de lesión en modelos de isquemia cerebral focal y global en ratas. *Biomédica*.

[206] Hamby M. E., Sofroniew M. V. (2010). Reactive astrocytes as therapeutic targets for CNS disorders. *Neurotherapeutics*.

[207] Volterra A., Meldolesi J. (2005). Astrocytes, from brain glue to communication elements: the revolution continues. *Nature Reviews Neuroscience*.

[208] Alvarez J. I., Katayama T., Prat A. (2013). Glial influence on the blood brain barrier. *Glia*.

[209] Arif M., Kato T. (2009). Increased expression of PAD2 after repeated intracerebroventricular infusions of soluble A*β*25–35 in the Alzheimer’s disease model rat brain: effect of memantine. *Cellular and Molecular Biology Letters*.

[210] Szeverenyi I., Cassidy A. J., Chung C. W. (2008). The human intermediate filament database: comprehensive information on a gene family involved in many human diseases. *Human Mutation*.

[211] Middeldorp J., Hol E. M. (2011). GFAP in health and disease. *Progress in Neurobiology*.

[212] Masutomi H., Kawashima S., Kondo Y. (2017). Induction of peptidylarginine deiminase 2 and 3 by dibutyryl cAMP via cAMP‐PKA signaling in human astrocytoma U‐251MG cells. *Journal of Neuroscience Research*.

[213] Nicholas A. P., Lu L., Heaven M. (2014). Ongoing studies of deimination in neurodegenerative diseases using the F95 antibody. *Protein Deimination in Human Health and Disease*.

[214] Ishigami A., Choi E. K., Kim Y. S., Maruyama N. (2014). Deimination in Alzheimer’s disease. *Protein Deimination in Human Health and Disease*.

[215] Xu K., Malouf A. T., Messing A., Silver J. (1999). Glial fibrillary acidic protein is necessary for mature astrocytes to react to *β*-amyloid. *Glia*.

[216] Nicholas A. P. (2013). Dual immunofluorescence study of citrullinated proteins in Alzheimer diseased frontal cortex. *Neuroscience Letters*.

[217] Jin Z., Fu Z., Yang J., Troncosco J., Everett A. D., van Eyk J. E. (2013). Identification and characterization of citrulline‐modified brain proteins by combining HCD and CID fragmentation. *Proteomics*.

[218] Mouser P. E., Head E., Ha K. H., Rohn T. T. (2006). Caspase-mediated cleavage of glial fibrillary acidic protein within degenerating astrocytes of the Alzheimer’s disease brain. *The American Journal of Pathology*.

[219] Olsen I., Singhrao S. K., Potempa J. (2018). Citrullination as a plausible link to periodontitis, rheumatoid arthritis, atherosclerosis and Alzheimer’s disease. *Journal of Oral Microbiology*.

[220] Liu C., Cui G., Zhu M., Kang X., Guo H. (2014). Neuroinflammation in Alzheimer’s disease: chemokines produced by astrocytes and chemokine receptors. *International Journal of Clinical and Experimental Pathology*.

[221] Acharya N. K., Nagele E. P., Han M. (2012). Neuronal PAD4 expression and protein citrullination: possible role in production of autoantibodies associated with neurodegenerative disease. *Journal of Autoimmunity*.

[222] Nagele R. G., D’Andrea M. R., Lee H., Venkataraman V., Wang H. Y. (2003). Astrocytes accumulate A*β*42 and give rise to astrocytic amyloid plaques in Alzheimer disease brains. *Brain Research*.

[223] Wyss-Coray T., Loike J. D., Brionne T. C. (2003). Adult mouse astrocytes degrade amyloid-*β* in vitro and in situ. *Nature Medicine*.

[224] Wegiel J., Wang K. . C., Tarnawski M., Lach B. (2000). Microglial cells are the driving force in fibrillar plaque formation, whereas astrocytes are a leading factor in plaque degradation. *Acta Neuropathologica*.

[225] Browne T. C., McQuillan K., McManus R. M., O’Reilly J. A., Mills K. H. G., Lynch M. A. (2013). IFN-*γ* production by amyloid *β*-specific Th1 cells promotes microglial activation and increases plaque burden in a mouse model of Alzheimer’s disease. *The Journal of Immunology*.

[226] Fisher Y., Nemirovsky A., Baron R., Monsonego A. (2010). T cells specifically targeted to amyloid plaques enhance plaque clearance in a mouse model of Alzheimer’s disease. *PLoS One*.

[227] Lambracht-Washington D., Qu B. X., Fu M. (2011). DNA immunization against amyloid beta 42 has high potential as safe therapy for Alzheimer’s disease as it diminishes antigen-specific Th1 and Th17 cell proliferation. *Cellular and Molecular Neurobiology*.

[228] Mosmann T. R., Coffman R. L. (1989). TH1 and TH2 cells: different patterns of lymphokine secretion lead to different functional properties. *Annual Review of Immunology*.

[229] Yin Y., Wen S. R., Li G. Z., Wang D. S. (2009). Hypoxia enhances stimulating effect of amyloid beta peptide (25–35) for interleukin 17 and T helper lymphocyte subtype 17 upregulation in cultured peripheral blood mononuclear cells. *Microbiology and Immunology*.

[230] Villa C., Fenoglio C., de Riz M. (2011). Role of *hnRNP-A1* and miR-590-3p in neuronal death: genetics and expression analysis in patients with Alzheimer disease and frontotemporal lobar degeneration. *Rejuvenation Research*.

[231] Saresella M., Calabrese E., Marventano I. (2010). PD1 negative and PD1 positive CD4^+^ T regulatory cells in mild cognitive impairment and Alzheimer’s disease. *Journal of Alzheimer’s Disease*.

[232] Saresella M., Calabrese E., Marventano I. (2011). Increased activity of Th-17 and Th-9 lymphocytes and a skewing of the post-thymic differentiation pathway are seen in Alzheimer’s disease. *Brain, Behavior, and Immunity*.

[233] Rosenkranz D., Weyer S., Tolosa E. (2007). Higher frequency of regulatory T cells in the elderly and increased suppressive activity in neurodegeneration. *Journal of Neuroimmunology*.

[234] Pellicanò M., Larbi A., Goldeck D. (2012). Immune profiling of Alzheimer patients. *Journal of Neuroimmunology*.

[235] Szabo S. J., Kim S. T., Costa G. L., Zhang X., Fathman C. G., Glimcher L. H. (2000). A novel transcription factor, T-bet, directs Th1 lineage commitment. *Cell*.

[236] Langrish C. L., Chen Y., Blumenschein W. M. (2005). IL-23 drives a pathogenic T cell population that induces autoimmune inflammation. *Journal of Experimental Medicine*.

[237] Zhang J., Ke K. F., Liu Z., Qiu Y. H., Peng Y. P. (2013). Th17 cell-mediated neuroinflammation is involved in neurodegeneration of A*β*_1-42_-induced Alzheimer’s disease model rats. *PLoS One*.

[238] Oppmann B., Lesley R., Blom B. (2000). Novel p19 protein engages IL-12p40 to form a cytokine, IL-23, with biological activities similar as well as distinct from IL-12. *Immunity*.

[239] O’Connor R. A., Anderton S. M. (2008). Foxp3^+^ regulatory T cells in the control of experimental CNS autoimmune disease. *Journal of Neuroimmunology*.

[240] Evans D. A., Funkenstein H. H., Albert M. S. (1989). Prevalence of Alzheimer’s disease in a community population of older persons. Higher than previously reported. *JAMA*.

[241] Obulesu M., Venu R., Somashekhar R. (2011). Lipid peroxidation in Alzheimer’s disease: emphasis on metal‐mediated neurotoxicity. *Acta Neurologica Scandinavica*.

[242] Alhazzani A. A., Alqahtani M., Alshbriqe A. (2017). Prevalence of complications associated with Alzheimer disease. *Journal of the Neurological Sciences*.

[243] Li Q., Wu H., Liao W. (2018). A comprehensive review of immune-mediated dermatopathology in systemic lupus erythematosus. *Journal of Autoimmunity*.

[244] Sherer Y., Gorstein A., Fritzler M. J., Shoenfeld Y. (2004). Autoantibody explosion in systemic lupus erythematosus: more than 100 different antibodies found in SLE patients. *Seminars in Arthritis and Rheumatism*.

[245] Tsokos G. C. (2011). Systemic lupus erythematosus. *The New England Journal of Medicine*.

[246] Hanly J. G., Su L., Urowitz M. B. (2015). Mood disorders in systemic lupus erythematosus: results from an international inception cohort study. *Arthritis & Rhematology*.

[247] Alarcón-Segovia D., Alarcón-Riquelme M. E., Cardiel M. H. (2005). Familial aggregation of systemic lupus erythematosus, rheumatoid arthritis, and other autoimmune diseases in 1,177 lupus patients from the GLADEL cohort. *Arthritis & Rheumatism*.

[248] Munoz L. E., van Bavel C., Franz S., Berden J., Herrmann M., van der Vlag J. (2008). Apoptosis in the pathogenesis of systemic lupus erythematosus. *Lupus*.

[249] He Y., Yang F. Y., Sun E. W. (2018). Neutrophil extracellular traps in autoimmune diseases. *Chinese Medical Journal*.

[250] Saraste A., Pulkki K. (2000). Morphologic and biochemical hallmarks of apoptosis. *Cardiovascular Research*.

[251] Rosen A., Casciola-Rosen L., Ahearn J. (1995). Novel packages of viral and self-antigens are generated during apoptosis. *Journal of Experimental Medicine*.

[252] Casciola-Rosen L. A., Anhalt G., Rosen A. (1994). Autoantigens targeted in systemic lupus erythematosus are clustered in two populations of surface structures on apoptotic keratinocytes. *Journal of Experimental Medicine*.

[253] Knight J. S., Carmona-Rivera C., Kaplan M. J. (2012). Proteins derived from neutrophil extracellular traps may serve as self-antigens and mediate organ damage in autoimmune diseases. *Frontiers in Immunology*.

[254] Jenne C. N., Wong C. H. Y., Zemp F. J. (2013). Neutrophils recruited to sites of infection protect from virus challenge by releasing neutrophil extracellular traps. *Cell Host & Microbe*.

[255] Urban C. F., Reichard U., Brinkmann V., Zychlinsky A. (2006). Neutrophil extracellular traps capture and kill *Candida albicans* yeast and hyphal forms. *Cellular Microbiology*.

[256] Abi Abdallah D. S., Denkers E. Y. (2012). Neutrophils cast extracellular traps in response to protozoan parasites. *Frontiers in Immunology*.

[257] Rahman S., Sagar D., Hanna R. N. (2019). Low-density granulocytes activate T cells and demonstrate a non-suppressive role in systemic lupus erythematosus. *Annals of the Rheumatic Diseases*.

[258] Pieterse E., van der Vlag J. (2014). Breaking immunological tolerance in systemic lupus erythematosus. *Frontiers in Immunology*.

[259] Neeli I., Radic M. (2012). Knotting the NETs: analyzing histone modifications in neutrophil extracellular traps. *Arthritis Research & Therapy*.

[260] Cheng O. Z., Palaniyar N. (2013). NET balancing: a problem in inflammatory lung diseases. *Frontiers in Immunology*.

[261] Liu C., Tangsombatvisit S., Rosenberg J. M. (2012). Specific post-translational histone modifications of neutrophil extracellular traps as immunogens and potential targets of lupus autoantibodies. *Arthritis Research & Therapy*.

[262] Peng Z., Mizianty M. J., Xue B., Kurgan L., Uversky V. N. (2012). More than just tails: intrinsic disorder in histone proteins. *Molecular BioSystems*.

[263] Hoppenbrouwers T., Autar A. S. A., Sultan A. R. (2017). In vitro induction of NETosis: comprehensive live imaging comparison and systematic review. *PLoS One*.

[264] Berthelot J. M., le Goff B., Neel A., Maugars Y., Hamidou M. (2017). NETosis: at the crossroads of rheumatoid arthritis, lupus, and vasculitis. *Joint Bone Spine*.

[265] Lee K. H., Kronbichler A., Park D. D. Y. (2017). Neutrophil extracellular traps (NETs) in autoimmune diseases: a comprehensive review. *Autoimmunity Reviews*.

[266] Pruchniak M. P., Demkow U. (2019). Potent NETosis inducers do not show synergistic effects in vitro. *Central European Journal of Immunology*.

[267] Ravindran M., Khan M. A., Palaniyar N. (2019). Neutrophil extracellular trap formation: physiology, pathology, and pharmacology. *Biomolecules*.

[268] Hamam H. J., Khan M. A., Palaniyar N. (2019). Histone acetylation promotes neutrophil extracellular trap formation. *Biomolecules*.

[269] Hamam H. J., Palaniyar N. (2019). Post-translational modifications in NETosis and NETs-mediated diseases. *Biomolecules*.

[270] Petretto A., Bruschi M., Pratesi F. (2019). Neutrophil extracellular traps (NET) induced by different stimuli: a comparative proteomic analysis. *PLoS One*.

[271] Delanghe S., Delanghe J. R., Speeckaert R., van Biesen W., Speeckaert M. M. (2017). Mechanisms and consequences of carbamoylation. *Nature Reviews Nephrology*.

[272] Delporte C., Zouaoui Boudjeltia K., Furtmüller P. G. (2018). Myeloperoxidase-catalyzed oxidation of cyanide to cyanate: a potential carbamylation route involved in the formation of atherosclerotic plaques?. *Journal of Biological Chemistry*.

[273] Spinelli F. R., Truglia S., Colasanti T. (2017). SAT0283 antibodies to carbamylated vimentin in patients with systemic lupus erythematosus are associated with renal involvement. *Annals of the Rheumatic Diseases*.

[274] Ceccarelli F., Perricone C., Colasanti T. (2018). Anti-carbamylated protein antibodies as a new biomarker of erosive joint damage in systemic lupus erythematosus. *Arthritis Research & Therapy*.

[275] Ceccarelli F., Sciandrone M., Perricone C. (2018). Biomarkers of erosive arthritis in systemic lupus erythematosus: application of machine learning models. *PLoS One*.

[276] Suurmond J., Diamond B. (2015). Autoantibodies in systemic autoimmune diseases: specificity and pathogenicity. *The Journal of Clinical Investigation*.

[277] Tan E. M., Cohen A. S., Fries J. F. (1982). The 1982 revised criteria for the classification of systemic lupus erythematosus. *Arthritis & Rheumatism*.

[278] Vas J., Grönwall C., Marshak-Rothstein A., Silverman G. J. (2012). Natural antibody to apoptotic cell membranes inhibits the proinflammatory properties of lupus autoantibody immune complexes. *Arthritis & Rheumatism*.

[279] Mannoor K., Matejuk A., Xu Y., Beardall M., Chen C. (2012). Expression of natural autoantibodies in MRL-lpr mice protects from lupus nephritis and improves survival. *The Journal of Immunology*.

[280] Zhen Q. L., Xie C., Wu T. (2005). Identification of autoantibody clusters that best predict lupus disease activity using glomerular proteome arrays. *The Journal of Clinical Investigation*.

[281] Malkiel S., Barlev A. N., Atisha-Fregoso Y., Suurmond J., Diamond B. (2018). Plasma cell differentiation pathways in systemic lupus erythematosus. *Frontiers in Immunology*.

[282] Mok C. C., Lau C. S. (2003). Pathogenesis of systemic lupus erythematosus. *Journal of Clinical Pathology*.

[283] Suzuki K., Sawada T., Murakami A. (2003). High diagnostic performance of ELISA detection of antibodies to citrullinated antigens in rheumatoid arthritis. *Scandinavian Journal of Rheumatology*.

[284] Vannini A., Cheung K., Fusconi M. (2007). Anti-cyclic citrullinated peptide positivity in non-rheumatoid arthritis disease samples: citrulline-dependent or not?. *Annals of the Rheumatic Diseases*.

[285] Taraborelli M., Inverardi F., Fredi M. (2012). Anti-cyclic citrullinated peptide antibodies in systemic lupus erythematosus patients with articular involvement: a predictive marker for erosive disease?. *Reumatismo*.

[286] Fava A., Petri M. (2019). Systemic lupus erythematosus: diagnosis and clinical management. *Journal of Autoimmunity*.

[287] Abdel-Magied R. A., AbuOmar H. A. S., Ali L. H., Talaat H., Mohamed F. I. (2019). Diagnostic potential of ultrasound in systemic lupus erythematosus patients with joint involvement: relation to anticyclic citrullinated peptide (anti-CCP), disease activity and functional status. *The Egyptian Rheumatologist*.

[288] Murugesan H., Mohanasundaram K. (2019). Anti-CCP antibodies and erosive arthropathy in systemic lupus erythematous. *International Journal of Scientific Research*.

[289] Martinez J. B., Valero J. S., Bautista A. J. (2007). Erosive arthropathy: clinical variance in lupus erythematosus and association with anti-CCP case series and review of the literature. *Clinical and Experimental Rheumatology*.

[290] Budhram A., Chu R., Rusta-Sallehy S. (2014). Anti-cyclic citrullinated peptide antibody as a marker of erosive arthritis in patients with systemic lupus erythematosus: a systematic review and meta-analysis. *Lupus*.

[291] Spinelli F. R., Colasanti T., Truglia S. (2018). PS2:27 antibodies to carbamylated vimentin in patients with systemic lupus erythematosus are associated with renal involvenment. *Lupus*.

[292] Massaro L., Ceccarelli F., Colasanti T. (2018). Anti-carbamylated protein antibodies in systemic lupus erythematosus patients with articular involvement. *Lupus*.

[293] Lu R., Munroe M. E., Guthridge J. M. (2016). Dysregulation of innate and adaptive serum mediators precedes systemic lupus erythematosus classification and improves prognostic accuracy of autoantibodies. *Journal of Autoimmunity*.

[294] Perl A. (2016). Activation of mTOR (mechanistic target of rapamycin) in rheumatic diseases. *Nature Reviews Rheumatology*.

[295] Moulton V. R., Tsokos G. C. (2015). T cell signaling abnormalities contribute to aberrant immune cell function and autoimmunity. *The Journal of Clinical Investigation*.

[296] Mohan C., Putterman C. (2015). Genetics and pathogenesis of systemic lupus erythematosus and lupus nephritis. *Nature Reviews Nephrology*.

[297] Tsokos G. C., Lo M. S., Reis P. C., Sullivan K. E. (2016). New insights into the immunopathogenesis of systemic lupus erythematosus. *Nature Reviews Rheumatology*.

[298] Munroe M. E., Lu R., Zhao Y. D. (2016). Altered type II interferon precedes autoantibody accrual and elevated type I interferon activity prior to systemic lupus erythematosus classification. *Annals of the Rheumatic Diseases*.

[299] Slight-Webb S., Lu R., Ritterhouse L. L. (2016). Autoantibody‐positive healthy individuals display unique immune profiles that may regulate autoimmunity. *Arthritis & Rhematology*.

[300] Xiao J. P., Wang D. Y., Wang X. R., Yuan L., Hao L., Wang D. G. (2018). Increased ratio of Th17 cells to SIGIRR^+^CD4^+^ T cells in peripheral blood of patients with SLE is associated with disease activity. *Biomedical Reports*.

[301] Dolff S., Abdulahad W. H., Westra J. (2011). Increase in IL-21 producing T-cells in patients with systemic lupus erythematosus. *Arthritis Research & Therapy*.

[302] Puwipirom H., Hirankarn N., Sodsai P., Avihingsanon Y., Wongpiyabovorn J., Palaga T. (2010). Increased interleukin-23 receptor^+^ T cells in peripheral blood mononuclear cells of patients with systemic lupus erythematosus. *Arthritis Research & Therapy*.

[303] Wang L., Wang F. S., Gershwin M. E. (2015). Human autoimmune diseases: a comprehensive update. *Journal of Internal Medicine*.

[304] Chavele K.-M., Ehrenstein M. R. (2011). Regulatory T‐cells in systemic lupus erythematosus and rheumatoid arthritis. *FEBS Letters*.

[305] Nakamura K., Kitani A., Strober W. (2001). Cell contact-dependent immunosuppression by CD4^+^CD25^+^ regulatory T cells is mediated by cell surface-bound transforming growth factor *β*. *Journal of Experimental Medicine*.

[306] Collison L. W., Workman C. J., Kuo T. T. (2007). The inhibitory cytokine IL-35 contributes to regulatory T-cell function. *Nature*.

[307] Rubtsov Y. P., Rasmussen J. P., Chi E. Y. (2008). Regulatory T cell-derived interleukin-10 limits inflammation at environmental interfaces. *Immunity*.

[308] de Smedt T., van Mechelen M., de Becker G., Urbain J., Leo O., Moser M. (1997). Effect of interleukin‐10 on dendritic cell maturation and function. *European Journal of Immunology*.

[309] Lee H. Y., Hong Y. K., Yun H. J., Kim Y. M., Kim J. R., Yoo W. H. (2008). Altered frequency and migration capacity of CD4^+^CD25^+^ regulatory T cells in systemic lupus erythematosus. *Rheumatology*.

[310] Valencia X., Yarboro C., Illei G., Lipsky P. E. (2007). Deficient CD4^+^CD25^high^ T regulatory cell function in patients with active systemic lupus erythematosus. *The Journal of Immunology*.

[311] Miyara M., Amoura Z., Parizot C. (2005). Global natural regulatory T cell depletion in active systemic lupus erythematosus. *The Journal of Immunology*.

[312] Bonelli M., von Dalwigk K., Savitskaya A., Smolen J. S., Scheinecker C. (2008). Foxp3 expression in CD4^+^ T cells of patients with systemic lupus erythematosus: a comparative phenotypic analysis. *Annals of the Rheumatic Diseases*.

[313] Abid N., Khan A. S., Otaibi F. H. A. (2013). Systemic lupus erythematosus (SLE) in the eastern region of Saudi Arabia. A comparative study. *Lupus*.

[314] Al-Motwee S., Jawdat D., Jehani G. S. (2013). Association of HLA-DRB1^∗^15 and HLADQB1^∗^06 with SLE in Saudis. *Annals of Saudi Medicine*.

[315] Qari F. A. (2002). Clinical pattern of systemic lupus erythematosus in Western Saudi Arabia. *Saudi Medical Journal*.

[316] Heller T., Ahmed M., Siddiqqi A., Wallrauch C., Bahlas S. (2007). Systemic lupus erythematosus in Saudi Arabia: morbidity and mortality in a multiethnic population. *Lupus*.

[317] Platt J., Burke B. A., Fish A. J., Kim Y., Michael A. F. (1982). Systemic lupus erythematosus in the first two decades of life. *American Journal of Kidney Diseases*.

[318] Brunner H. I., Gladman D. D., Ibañez D., Urowitz M. D., Silverman E. D. (2008). Difference in disease features between childhood‐onset and adult‐onset systemic lupus erythematosus. *Arthritis & Rheumatism*.

[319] Al-Homood I. A., Omran N. E., Alwahibi A. S., Aldosoghy M., Alharthy A., Aljohani G. S. (2017). Depression in patients with systemic lupus erythematosus: a multicenter study. *Saudi Journal of Medicine and Medical Sciences*.

[320] Alharbi M. D., Aljohani B. O., Alaithan Z. N. (2018). Public awareness of systemic lupus erythematosus in Al-Dammam City in Saudi Arabia. *The Egyptian Journal of Hospital Medicine*.

[321] Kourilovitch M., Galarza-Maldonado C., Ortiz-Prado E. (2014). Diagnosis and classification of rheumatoid arthritis. *Journal of Autoimmunity*.

[322] Symmons D., Turner G., Webb R. (2002). The prevalence of rheumatoid arthritis in the United Kingdom: new estimates for a new century. *Rheumatology*.

[323] Lawrence R. C., Felson D. T., Helmick C. G. (2008). Estimates of the prevalence of arthritis and other rheumatic conditions in the United States: part II. *Arthritis & Rheumatism*.

[324] McInnes I. B., Schett G. (2011). The pathogenesis of rheumatoid arthritis. *The New England Journal of Medicine*.

[325] Gabriel S. E. (2001). The epidemiology of rheumatoid arthritis. *Rheumatic Diseases Clinics of North America*.

[326] Viatte S., Plant D., Raychaudhuri S. (2013). Genetics and epigenetics of rheumatoid arthritis. *Nature Reviews Rheumatology*.

[327] Ospelt C., Gay S. (2012). Epigenetic epidemiology of inflammation and rheumatoid arthritis. *Epigenetic Epidemiology*.

[328] Lajas C., Abasolo L., Bellajdel B. (2003). Costs and predictors of costs in rheumatoid arthritis: a prevalence‐based study. *Arthritis & Rheumatism*.

[329] Malmstrom V., Catrina A. I., Klareskog L. (2017). The immunopathogenesis of seropositive rheumatoid arthritis: from triggering to targeting. *Nature Reviews Immunology*.

[330] Klareskog L., Rönnelid J., Lundberg K., Padyukov L., Alfredsson L. (2008). Immunity to citrullinated proteins in rheumatoid arthritis. *Annual Review of Immunology*.

[331] Firestein G. S., McInnes I. B. (2017). Immunopathogenesis of rheumatoid arthritis. *Immunity*.

[332] Gregersen P. K., Silver J., Winchester R. J. (1987). The shared epitope hypothesis. An approach to understanding the molecular genetics of susceptibility to rheumatoid arthritis. *Arthritis & Rheumatism*.

[333] Angelotti F., Parma A., Cafaro G., Capecchi R., Alunno A., Puxeddu I. (2017). One year in review 2017: pathogenesis of rheumatoid arthritis. *Clinical and Experimental Rheumatology*.

[334] Kallberg H., Ding B., Padyukov L. (2011). Smoking is a major preventable risk factor for rheumatoid arthritis: estimations of risks after various exposures to cigarette smoke. *Annals of the Rheumatic Diseases*.

[335] Sparks J. A., Chang S. C., Deane K. D. (2016). Associations of smoking and age with inflammatory joint signs among unaffected first‐degree relatives of rheumatoid arthritis patients: results from studies of the etiology of rheumatoid arthritis. *Arthritis & Rhematology*.

[336] Webber M. P., Berman J., Qayyum B., Jaber N., Prezant D. J. (2015). Reply. *Arthritis & Rhematology*.

[337] Stolt P., Källberg H., Lundberg I. (2005). Silica exposure is associated with increased risk of developing rheumatoid arthritis: results from the Swedish EIRA study. *Annals of the Rheumatic Diseases*.

[338] Too C. L., Muhamad N. A., Ilar A. (2016). Occupational exposure to textile dust increases the risk of rheumatoid arthritis: results from a Malaysian population-based case-control study. *Annals of the Rheumatic Diseases*.

[339] Chen J., Wright K., Davis J. M. (2016). An expansion of rare lineage intestinal microbes characterizes rheumatoid arthritis. *Genome Medicine*.

[340] Berthelot J. M., Le Goff B. (2010). Rheumatoid arthritis and periodontal disease. *Joint Bone Spine*.

[341] Mikuls T. R., Payne J. B., Reinhardt R. A. (2009). Antibody responses to *Porphyromonas gingivalis* (*P. gingivalis*) in subjects with rheumatoid arthritis and periodontitis. *International Immunopharmacology*.

[342] Hueber W., Kidd B. A., Tomooka B. H. (2005). Antigen microarray profiling of autoantibodies in rheumatoid arthritis. *Arthritis & Rheumatism*.

[343] Jaskowski T. D., Hill H. R., Russo K. L., Lakos G., Szekanecz Z., Teodorescu M. (2010). Relationship between rheumatoid factor isotypes and IgG anti-cyclic citrullinated peptide antibodies. *The Journal of Rheumatology*.

[344] De Rycke L., Nicholas A. P., Cantaert T. (2005). Synovial intracellular citrullinated proteins colocalizing with peptidyl arginine deiminase as pathophysiologically relevant antigenic determinants of rheumatoid arthritis-specific humoral autoimmunity. *Arthritis & Rheumatism*.

[345] Chang X., Yamada R., Suzuki A. (2005). Localization of peptidylarginine deiminase 4 (PADI4) and citrullinated protein in synovial tissue of rheumatoid arthritis. *Rheumatology*.

[346] Yamada R., Suzuki A., Yamamoto K. (2006). Citrulline and anti-cyclic citrullinated peptide antibodies in rheumatoid arthritis. *Future Rheumatology*.

[347] Trela M., Perera S., Sheeran T., Rylance P., Nelson P. N., Attridge K. (2019). Citrullination facilitates cross-reactivity of rheumatoid factor with non-IgG1 Fc epitopes in rheumatoid arthritis. *Scientific Reports*.

[348] Chang X., Yamada R., Sawada T., Suzuki A., Kochi Y., Yamamoto K. (2005). The inhibition of antithrombin by peptidylarginine deiminase 4 may contribute to pathogenesis of rheumatoid arthritis. *Rheumatology*.

[349] Masson-Bessière C., Sebbag M., Girbal-Neuhauser E. (2001). The major synovial targets of the rheumatoid arthritis-specific antifilaggrin autoantibodies are deiminated forms of the *α*- and *β*-chains of fibrin. *The Journal of Immunology*.

[350] Cau L., Mechin M. C., Simon M. (2018). Peptidylarginine deiminases and deiminated proteins at the epidermal barrier. *Experimental Dermatology*.

[351] Aliko A., Kamińska M., Falkowski K. (2019). Discovery of novel potential reversible peptidyl arginine deiminase inhibitor. *International Journal of Molecular Sciences*.

[352] Darrah E., Andrade F. (2018). Rheumatoid arthritis and citrullination. *Current Opinion in Rheumatology*.

[353] Carubbi F., Alunno A., Gerli R., Giacomelli R. (2019). Post-translational modifications of proteins: novel insights in the autoimmune response in rheumatoid arthritis. *Cells*.

[354] Tilvawala R., Nguyen S. H., Maurais A. J. (2018). The rheumatoid arthritis-associated citrullinome. *Cell Chemical Biology*.

[355] Spengler J., Lugonja B., Jimmy Ytterberg A. (2015). Release of active peptidyl arginine deiminases by neutrophils can explain production of extracellular citrullinated autoantigens in rheumatoid arthritis synovial fluid. *Arthritis & Rhematology*.

[356] Carmona-Rivera C., Bicker K. L., Thompson P. R. (2018). Response to comment on “Synovial fibroblast-neutrophil interactions promote pathogenic adaptive immunity in rheumatoid arthritis”. *Science Immunology*.

[357] Stanford S. M., Aleman Muench G. R., Bartok B. (2016). TGF*β* responsive tyrosine phosphatase promotes rheumatoid synovial fibroblast invasiveness. *Annals of the Rheumatic Diseases*.

[358] Smolen J. S., Aletaha D., Barton A. (2018). Rheumatoid arthritis. *Nature Reviews Disease Primers*.

[359] van der Velden D., Lagraauw H. M., Wezel A. (2016). Mast cell depletion in the preclinical phase of collagen-induced arthritis reduces clinical outcome by lowering the inflammatory cytokine profile. *Arthritis Research & Therapy*.

[360] Smolen J. S., Steiner G. (2003). Therapeutic strategies for rheumatoid arthritis. *Nature Reviews Drug Discovery*.

[361] Smolen J. S., Aletaha D., Koeller M., Weisman M. H., Emery P. (2007). New therapies for treatment of rheumatoid arthritis. *The Lancet*.

[362] Choy E. (2012). Understanding the dynamics: pathways involved in the pathogenesis of rheumatoid arthritis. *Rheumatology*.

[363] Scherer H. U., Huizinga T. W. J., Krönke G., Schett G., Toes R. E. M. (2018). The B cell response to citrullinated antigens in the development of rheumatoid arthritis. *Nature Reviews Rheumatology*.

[364] Chabaud M., Fossiez F., Taupin J. L., Miossec P. (1998). Enhancing effect of IL-17 on IL-1-induced IL-6 and leukemia inhibitory factor production by rheumatoid arthritis synoviocytes and its regulation by Th2 cytokines. *The Journal of Immunology*.

[365] Yap H.-Y., Tee S., Wong M., Chow S.-K., Peh S.-C., Teow S.-Y. (2018). Pathogenic role of immune cells in rheumatoid arthritis: implications in clinical treatment and biomarker development. *Cells*.

[366] Nadkarni S., Mauri C., Ehrenstein M. R. (2007). Anti-TNF-*α* therapy induces a distinct regulatory T cell population in patients with rheumatoid arthritis via TGF-*β*. *Journal of Experimental Medicine*.

[367] Kotake S., Nanke Y., Yago T., Kawamoto M., Kobashigawa T., Yamanaka H. (2016). Elevated ratio of Th17 cell-derived Th1 cells (CD161^+^Th1 cells) to CD161^+^Th17 cells in peripheral blood of early-onset rheumatoid arthritis patients. *BioMed Research International*.

[368] Neidhart S., Neidhart M. (2019). Rheumatoid arthritis and the concept of autoimmune disease. *International Journal of Clinical Rheumatology*.

[369] Tanaka T., Narazaki M., Kishimoto T. (2014). IL-6 in inflammation, immunity, and disease. *Cold Spring Harbor Perspectives in Biology*.

[370] Zendman A. J. W., van Venrooij W. J., Pruijn G. J. M. (2006). Use and significance of anti-CCP autoantibodies in rheumatoid arthritis. *Rheumatology*.

[371] Mota L. M., Cruz B. A., Brenola C. V. (2013). Guidelines for the diagnosis of rheumatoid arthritis. *Revista Brasileira de Reumatologia*.

[372] Dixon W., Symmons D. (2005). Does early rheumatoid arthritis exist?. *Best Practice & Research Clinical Rheumatology*.

[373] Woolf A. D. (2003). History and physical examination. *Best Practice & Research. Clinical Rheumatology*.

[374] Goeldner I., Skare T. L., de Messias Reason I. T., Nisihara R. M., Silva M. B., da Rosa Utiyama S. R. (2011). Association of anticyclic citrullinated peptide antibodies with extra-articular manifestations, gender, and tabagism in rheumatoid arthritis patients from Southern Brazil. *Clinical Rheumatology*.

[375] Turesson C., Eberhardt K., Jacobsson L. T. H., Lindqvist E. (2007). Incidence and predictors of severe extra-articular disease manifestations in an early rheumatoid arthritis inception cohort. *Annals of the Rheumatic Diseases*.

[376] Arnett F. C., Edworthy S. M., Bloch D. A. (1988). The American Rheumatism Association 1987 revised criteria for the classification of rheumatoid arthritis. *Arthritis & Rheumatism*.

[377] Kaarela K., Kauppi M. J., Lehtinen K. E. S. (1995). The value of the ACR 1987 criteria in very early rheumatoid arthritis. *Scandinavian Journal of Rheumatology*.

[378] Harrison B. J., Symmons D. P., Barrett E. M., Silman A. J. (1998). The performance of the 1987 ARA classification criteria for rheumatoid arthritis in a population based cohort of patients with early inflammatory polyarthritis. American Rheumatism Association. *The Journal of Rheumatology*.

[379] Aletaha D., Neogi T., Silman A. J. (2010). 2010 Rheumatoid arthritis classification criteria: an American College of Rheumatology/European League Against Rheumatism collaborative initiative. *Arthritis & Rheumatism*.

[380] Berglin E., Dahlqvist S. R. (2013). Comparison of the 1987 ACR and 2010 ACR/EULAR classification criteria for rheumatoid arthritis in clinical practice: a prospective cohort study. *Scandinavian Journal of Rheumatology*.

[381] Alamri S. Z. S., Alali M. A. E. (2016). Rheumatoid arthritis in Hail Region, Saudi Arabia. *International Journal of Innovative Research in Medical Science*.

[382] Al-Dalaan A., al Ballaa S., Bahabri S., Biyari T., al Sukait M., Mousa M. (1998). The prevalence of rheumatoid arthritis in the Qassim region of Saudi Arabia. *Annals of Saudi Medicine*.

[383] Trouw L. A., Huizinga T. W. J., Toes R. E. M. (2013). Autoimmunity in rheumatoid arthritis: different antigens—common principles. *Annals of the Rheumatic Diseases*.

[384] Fox D. A. (2015). Citrullination: a specific target for the autoimmune response in rheumatoid arthritis. *The Journal of Immunology*.

[385] Pratesi F., Panza F., Paolini I. (2015). Fingerprinting of anti-citrullinated protein antibodies (ACPA): specificity, isotypes and subclasses. *Lupus*.

[386] Holers V. M. (2013). Autoimmunity to citrullinated proteins and the initiation of rheumatoid arthritis. *Current Opinion in Immunology*.

[387] Matsuo K., Xiang Y., Nakamura H. (2006). Identification of novel citrullinated autoantigens of synovium in rheumatoid arthritis using a proteomic approach. *Arthritis Research & Therapy*.

[388] Tilleman K., van Steendam K., Cantaert T., de Keyser F., Elewaut D., Deforce D. (2008). Synovial detection and autoantibody reactivity of processed citrullinated isoforms of vimentin in inflammatory arthritides. *Rheumatology*.

[389] Kinloch A., Tatzer V., Wait R. (2005). Identification of citrullinated *α*-enolase as a candidate autoantigen in rheumatoid arthritis. *Arthritis Research & Therapy*.

[390] Kinloch A., Lundberg K., Wait R. (2008). Synovial fluid is a site of citrullination of autoantigens in inflammatory arthritis. *Arthritis & Rheumatism*.

[391] Yoshida M., Tsuji M., Kurosaka D. (2006). Autoimmunity to citrullinated type II collagen in rheumatoid arthritis. *Modern Rheumatology*.

[392] Schellekens G. A., de Jong B. A., van den Hoogen F. H., van de Putte L. B., van Venrooij W. J. (1998). Citrulline is an essential constituent of antigenic determinants recognized by rheumatoid arthritis-specific autoantibodies. *The Journal of Clinical Investigation*.

[393] Mor-Vaknin N., Punturieri A., Sitwala K., Markovitz D. M. (2003). Vimentin is secreted by activated macrophages. *Nature Cell Biology*.

[394] Wegner N., Lundberg K., Kinloch A. (2010). Autoimmunity to specific citrullinated proteins gives the first clues to the etiology of rheumatoid arthritis. *Immunological Reviews*.

[395] Tesija-Kuna A., Grazio S., Miler M., Vukasovic I., Peric P., Vrkic N. (2010). Antibodies targeting mutated citrullinated vimentin in patients with psoriatic arthritis. *Clinical Rheumatology*.

[396] Borders C. L., Broadwater J. A., Bekeny P. A. (1994). A structural role for arginine in proteins: multiple hydrogen bonds to backbone carbonyl oxygens. *Protein Science*.

[397] Goldbach-Mansky R., Lee J., McCoy A. (2000). Rheumatoid arthritis associated autoantibodies in patients with synovitis of recent onset. *Arthritis Research*.

[398] Liu X., Jia R., Zhao J., Li Z. (2009). The role of anti-mutated citrullinated vimentin antibodies in the diagnosis of early rheumatoid arthritis. *The Journal of Rheumatology*.

[399] Arkema E. V., Goldstein B. L., Robinson W. (2013). Anti-citrullinated peptide autoantibodies, human leukocyte antigen shared epitope and risk of future rheumatoid arthritis: a nested case-ontrol study. *Arthritis Research & Therapy*.

[400] Westwood O. M. R., Nelson P. N., Hay F. C. (2006). Rheumatoid factors: what’s new?. *Rheumatology*.

[401] Nijenhuis S., Zendman A. J. W., Vossenaar E. R., Pruijn G. J. M., vanVenrooij W. J. (2004). Autoantibodies to citrullinated proteins in rheumatoid arthritis: clinical performance and biochemical aspects of an RA-specific marker. *Clinica Chimica Acta*.

[402] Schellekens G. A., Visser H., de Jong B. A. W. (2000). The diagnostic properties of rheumatoid arthritis antibodies recognizing a cyclic citrullinated peptide. *Arthritis & Rheumatism*.

[403] Pruijn G. J., Wiik A., van Venrooij W. J. (2010). The use of citrullinated peptides and proteins for the diagnosis of rheumatoid arthritis. *Arthritis Research & Therapy*.

[404] Wiik A. S., van Venrooij W. J., Pruijn G. J. M. (2010). All you wanted to know about anti-CCP but were afraid to ask. *Autoimmunity Reviews*.

[405] van Venrooij W. J., Zendman A. J. W. (2008). Anti-CCP2 antibodies: an overview and perspective of the diagnostic abilities of this serological marker for early rheumatoid arthritis. *Clinical Reviews in Allergy & Immunology*.

[406] van Venrooij W. J., Hazes J. M., Visser H. (2002). Anticitrullinated protein/peptide antibody and its role in the diagnosis and prognosis of early rheumatoid arthritis. *The Netherlands Journal of Medicine*.

[407] Landewé R. B. M., Boers M., Verhoeven A. C. (2002). COBRA combination therapy in patients with early rheumatoid arthritis: long‐term structural benefits of a brief intervention. *Arthritis & Rheumatism*.

[408] Sebbag M., Simon M., Vincent C. (1995). The antiperinuclear factor and the so-called antikeratin antibodies are the same rheumatoid arthritis-specific autoantibodies. *The Journal of Clinical Investigation*.

[409] Mahdi H., Fisher B. A., Källberg H. (2009). Specific interaction between genotype, smoking and autoimmunity to citrullinated *α*-enolase in the etiology of rheumatoid arthritis. *Nature Genetics*.

[410] INOVA Diagnostics QUANTA Lite CCP 3.1 IgG/IgA ELISA package insert, revision 2. https://www.inovadx.com/pdf/di/704550_en.pdf.

[411] von Budingen H. C., Hauser S. L., Fuhrmann A., Nabavi C. B., Lee J. I., Genain C. P. (2002). Molecular characterization of antibody specificities against myelin/oligodendrocyte glycoprotein in autoimmune demyelination. *Proceedings of the National Academy of Sciences of the United States of America*.

[412] van Gaalen F. A., van Aken J., Huizinga T. W. J. (2004). Association between HLA class II genes and autoantibodies to cyclic citrullinated peptides (CCPs) influences the severity of rheumatoid arthritis. *Arthritis & Rheumatism*.

[413] Senshu T., Sato T., Inoue T., Akiyama K., Asaga H. (1992). Detection of citrulline residues in deiminated proteins on polyvinylidene difluoride membrane. *Analytical Biochemistry*.

[414] Mermod N., Williams T. J., Tjian R. (1988). Enhancer binding factors AP-4 and AP-1 act in concert to activate SV40 late transcription in vitro. *Nature*.

[415] Bang H., Egerer K., Gauliard A. (2007). Mutation and citrullination modifies vimentin to a novel autoantigen for rheumatoid arthritis. *Arthritis & Rheumatism*.

[416] Lee Y. H., Bae S. C., Song G. G. (2015). Diagnostic accuracy of anti-MCV and anti-CCP antibodies in rheumatoid arthritis. *Zeitschrift für Rheumatologie*.

[417] Luime J. J., Colin E. M., Hazes J. M. W., Lubberts E. (2010). Does anti-mutated citrullinated vimentin have additional value as a serological marker in the diagnostic and prognostic investigation of patients with rheumatoid arthritis? A systematic review. *Annals of the Rheumatic Diseases*.

[418] el Shazly R. I., Hussein S. A., Raslan H. Z., Elgogary A. A. (2014). Anti-mutated citrullinated vimentin antibodies in rheumatoid arthritis patients: relation to disease activity and manifestations. *The Egyptian Rheumatologist*.

[419] Safi M., Houssien D., Scott D. (2012). Disease activity and anti-cyclic citrullinated peptide (anti-CCP) antibody in Saudi RF-negative rheumatoid arthritis patients. *Journal of King Abdulaziz University-Medical Sciences*.

[420] Alrogy A., Dirar A., Alrogy W., Fakhoury H., Hajeer A. (2017). Association of human leukocyte antigen-DRB1 with anti-cyclic citrullinated peptide autoantibodies in Saudi patients with rheumatoid arthritis. *Annals of Saudi Medicine*.

[421] Safi M. A., Attar S. M., Fathaldin O. A., Safi O. M. (2015). Anti-mutated citrullinated vimentin antibody and rheumatoid factor (prevalence and association) in rheumatoid arthritis patients; Saudi and non-Saudi. *Clinical Laboratory*.

